# In-matrix library preparation for metagenomic sequencing of microbial cell-free DNA

**DOI:** 10.1128/jcm.00944-25

**Published:** 2025-11-28

**Authors:** Paul L. Babb, Jamilla Akhund-Zade, Damek Spacek, Kevin Brick, Fred C. Christians, Victoria Portnoy, Ming-Shian Tsai, Kristin H. Jarman, Sivan Bercovici, Igor D. Vilfan, Timothy A. Blauwkamp

**Affiliations:** 1 Karius, Inc. 597799 , Redwood City, California, USA; Vanderbilt University Medical Center, Nashville, Tennessee, USA

**Keywords:** liquid biopsy, DNA library protocol, extraction-free, microbial cell-free DNA, in-matrix, metagenomics

## Abstract

Metagenomic sequencing of microbial cell-free DNA (mcfDNA) enables comprehensive identification and quantification of diverse pathogens from blood and other biofluids. This approach enables minimally invasive diagnosis of deep-seated infectious disease, provides culture-free identification of antimicrobial resistance, and powers the discovery of novel microbial biomarkers for disease. However, widespread implementation of this approach is limited by lengthy and complex workflows, high host background cfDNA leading to high sequencing costs, and prevalent environmental DNA contamination risks. Addressing these barriers is critical for scalable deployment in both centralized and decentralized settings. To overcome these limitations, we developed Karius Helion-4 Chemistry (Helion-4), an in-matrix (DNA extraction-free) sample-to-DNA sequencing library workflow, to serve as a platform for mcfDNA sequencing applications in infectious disease, microbiome analyses, and disease biomarker discovery. We compared Helion-4 to two widely used metagenomic extraction-based sequencing workflows, as well as to the prior Karius chemistry platform (Digital Culture-3), using 36 clinical plasma specimens. Helion-4 enables end-to-end sequencing library construction for up to 96 samples in 5.25–6.1 h, including setup and final quality control evaluation, with 2.25 h of hands-on time when using automated liquid handling robots. Compared to the other methods, Helion-4 recovered 58-fold to 817-fold more endogenous mcfDNA per volume of plasma, while simultaneously demonstrating 1.8-fold to 6-fold lower exogenous background DNA contamination, likely due to the absence of DNA extraction. The fraction of mcfDNA reads among total reads was enriched by 60-fold to 164-fold for Helion-4 compared to current state-of-the-art methods, significantly lowering sequencing costs required for applications built on the Helion-4 platform vs other platforms. Collectively, these advances enable routine processing of small specimen volumes and provide a simple, efficient, and scalable approach for mcfDNA sequencing applications.

**IMPORTANCE:**

Metagenomic sequencing of microbial cell-free DNA (mcfDNA) enables the identification and quantification of diverse pathogens from blood and other biofluids, providing minimally invasive and rapid diagnosis of deep-seated infectious disease. However, widespread implementation of this approach is limited by complex workflows, high sequencing costs, and prevalent contamination risks. Karius Helion-4 Chemistry, the first in-matrix (DNA extraction-free) sample-to-DNA sequencing library workflow, overcomes these limitations. Compared to the other methods, Helion-4 is faster, cleaner, and more sensitive. Helion-4 recovered up to 817-fold more endogenous mcfDNA per volume of plasma, while simultaneously demonstrating up to sixfold lower exogenous background DNA contamination. The fraction of mcfDNA reads among total reads was enriched by up to 164-fold for Helion-4, lowering sequencing costs. These advances by Helion-4 technology enable a simple, efficient, and scalable approach for mcfDNA sequencing applications and bring us closer to widespread, high-resolution, and real-time microbial profiling across diverse healthcare settings.

## INTRODUCTION

Cell-free DNA (cfDNA) sequencing has revolutionized disease diagnostics and management by offering a minimally invasive method for accessing biological information from various tissues throughout the body (1–3). This advancement has facilitated the development of several US Food and Drug Administration-authorized diagnostic tests for diverse clinical applications, including fetal health assessment, solid organ transplant rejection, and oncologic evaluations based on cfDNA analysis in plasma (2, 4–7). Similar to human cfDNA (hcfDNA), microbial cell-free DNA (mcfDNA) is also shed into circulation by the replicating and dying microbes in humans (8, 9), providing a unique opportunity to leverage mcfDNA sequencing for minimally invasive diagnostics in infectious disease, gut health monitoring, autoimmune disorders, and other microbiome-associated conditions (10, 11).

Several studies have shown that plasma mcfDNA sequencing enables detection of pathogens in deep tissue and bloodstream infections (8, 12, 13), often with greater sensitivity than conventional methods (13–15). Adding mcfDNA sequencing to standard diagnostics has increased etiological detection rates by 10% to >100% (15–18). Currently offered via centralized reference labs, a real-world study of over 18,000 tests reported a median sample receipt-to-result time of 26 h, faster than many culture-based or send-out tests. However, shipping adds delays, extending total turnaround to an average of 2.6 days (19) and hindering the processing of international samples in a centralized US laboratory. Distributed testing could eliminate shipping, improve access and equity, and accelerate clinical decision-making.

Several technical challenges must be addressed to enable widespread clinical implementation of mcfDNA sequencing. Current library workflows are labor-intensive, typically requiring multiple days for completion, and available sample preparation kits are highly inefficient for fragmented and degraded DNA fragments such as mcfDNA (20). Furthermore, exogenous microbial DNA contamination from reagents and consumables poses a significant barrier to accurate microbial profiling (21–24), while the inherently low abundance of mcfDNA relative to host-derived cfDNA necessitates highly effective enrichment strategies to enable reasonable sequencing depths.

To address these challenges, we developed the Karius Helion-4 in-matrix library preparation platform (Helion-4). Helion-4 simplifies the testing workflow by eliminating the need for DNA extraction altogether, providing an end-to-end laboratory workflow that is easily completed in a single standard 8 h work shift. In this study, we compare the performance of Helion-4 on plasma specimens to two commonly employed alternative laboratory methods for mcfDNA metagenomic library generation, and to the prior Karius platform: Digital Culture-3 (DC3, provided by the Karius Laboratory until November 2024). We compare these approaches on workflow complexity, mcfDNA recovery, environmental contamination, and mcfDNA enrichment to determine the most effective approach for implementing metagenomic sequencing of mcfDNA applications.

## MATERIALS AND METHODS

### Samples

Residual de-identified plasma samples from 36 patients with suspected infections were selected as the testing set to evaluate and compare library preparation workflows. To enrich for a diverse representation of endogenous mcfDNA, samples associated with evidence of polymicrobial infections were included. Selection criteria further prioritized diversity in microbial phylogenetic lineages, genomic GC content, and mcfDNA concentrations in plasma (Fig. S1). Each library preparation method was accompanied by eight no-template controls, denoted herein as environmental contamination control samples, to quantify method-specific background signals in real time. For Karius DC3 and Helion-4, a biochemically matched EC buffer was utilized to mimic the physicochemical properties of plasma. In contrast, the external comparative workflows (described below) incorporated 1× IDTE (Integrated DNA Technologies) as the EC buffer, consistent with the published methods for these approaches (Table S1).

Prior to library preparation, all plasma and environmental control (EC) samples were thawed and spiked using a patented process with a defined concentration of synthetic internal control DNA molecules to enable downstream normalization and quality control (13). All samples were de-identified and unlinked from any personal identifiers. Accordingly, the study was determined not to involve human subjects as defined by applicable regulatory standards.

### Selection of comparative sequencing library generation processes

Alternative workflows were identified by searching PubMed for studies describing metagenomic sequencing of mcfDNA from low-biomass, noninvasive sample types (e.g., plasma, urine). Studies were included if they explicitly described their workflows, including DNA extraction and library preparation methods, or referenced a standardized workflow. We excluded studies that employed targeted sequencing approaches (e.g., 16S rRNA gene sequencing), analyzed intact microbial cells rather than cfDNA, focused on high-biomass sample types (e.g., saliva, stool), or insufficiently described protocols lacking defined reporting thresholds and quality control procedures. Of the initial 291 eligible publications, 276 focused on the earlier version of the Karius Test (DC3), resulting in 15 studies qualifying for comparative performance assessment (Table S1) (2, 13, 20, 25–36). This search identified two protocols, denoted as Ext+dsDNA and Ext+ssDNA, that are described in detail below.

**Fig 1:**
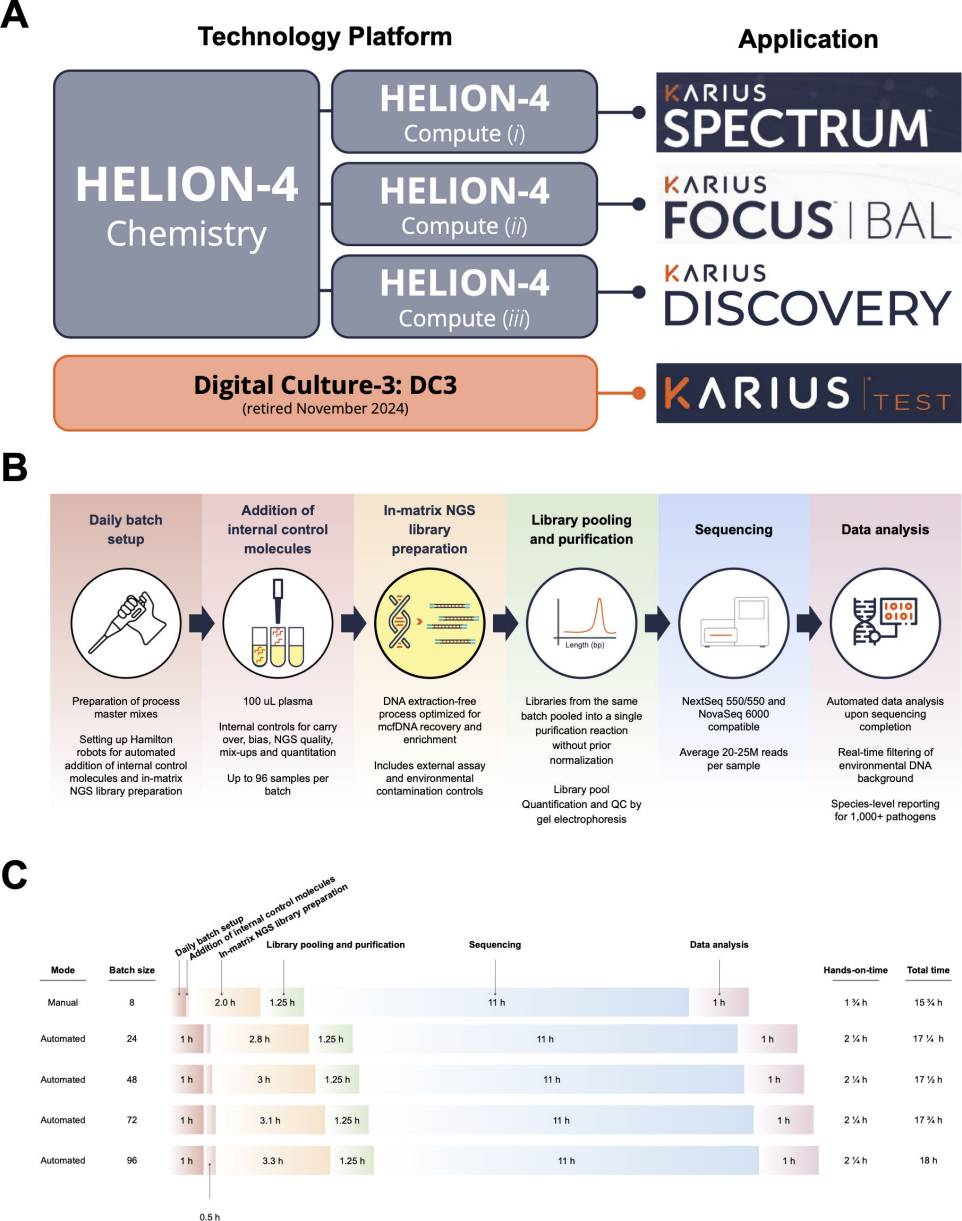
The Karius Helion-4 technology platform overview and operational performance. (**A**) The relationship of different technology platforms at Karius (DC3 and Helion-4) to distinct clinical and discovery assays (Karius Spectrum, Focus, and Discovery) where different processing specifications, computational filters, and clinical pathogen databases are applied to distinct reporting needs. (**B**) The Karius Helion-4 technology platform combines multiple key steps for the optimized recovery of mcfDNAs. (**C**) Real-world timing of the Karius Helion-4 workflow as executed by clinical laboratory scientists in a CLIA/CAP/NYSDOH reference lab across multiple batches of varying size. A manual mode to process eight samples is included for reference.

In addition, we evaluated the performance of Karius Helion-4 alongside the previous Karius DC3 technology platform. The Karius DC3 platform powered the earlier generation of the Karius Test clinical assay until November 2024, when Helion-4 was introduced as the platform for the Karius Spectrum infectious disease test (Fig. 1A).

### Sequencing library generation

Aliquots from each pre-spiked plasma sample were processed using four distinct library preparation protocols: Karius Helion-4, Karius DC3, Ext+dsDNA, and Ext+ssDNA. The Karius Helion-4 protocol, developed by Karius, Inc. (Redwood City, CA, USA), utilizes 25 µL of plasma and performs library construction directly within the plasma itself (in-matrix). This process eliminates the need for prior DNA extraction or purification steps. Plasma is spiked using a mixture of control molecules, as described in reference 13. Plasma proteins are digested in-matrix, and adapters are ligated to single-stranded DNA fragments. The ligated fragments are then PCR amplified using dual-indexed Illumina-ready primers.

In contrast, Karius DC3, Ext+dsDNA, and Ext+ssDNA all employ cfDNA extraction prior to library preparation. For Karius DC3, cfDNA was extracted from 250 µL of spiked plasma using a modified Mag-Bind cfDNA Kit (Omega Biotek) protocols, and libraries were constructed using Ovation Ultralow System V2 library preparation kits (Tecan) (13). Both Ext+dsDNA and Ext+ssDNA workflows employed the QIAamp Circulating Nucleic Acid Kit (Qiagen) for cfDNA extraction. For these protocols, 250 µL of spiked plasma was mixed with 750 µL of 1× phosphate-buffered saline, followed by extraction and elution into 60 µL of AVE buffer, as per the manufacturer’s instructions. For the Ext+dsDNA workflow, 24 µL of the extracted cfDNA was processed using the NEBNext Ultra II DNA Library Prep Kit (New England Biolabs), incorporating 10 cycles of PCR during final library amplification. For the Ext+ssDNA workflow, 24 µL of the same extracted cfDNA was processed using the SRSLY PicoPlus NGS Library Prep Kit (Claret Bioscience), also with 10 PCR cycles during the final amplification step. All comparisons account for the differences in plasma volumes input into the various methods.

### Purification, quantification, and sequencing of libraries

For each library preparation method, pools were generated from amplified libraries prior to purification. Each pool consisted of 18 specimen libraries and 4 EC libraries, yielding two purified library pools per method. Both Karius Helion-4 and Karius DC3 library pools underwent purification using two distinct proprietary protocols specifically designed to enrich mcfDNA. For the Ext+dsDNA protocol, purification was performed using Agencourt RNAClean XP beads (Beckman Coulter), following the manufacturer’s recommendations. Ext+ssDNA library pools were purified using Clarefy beads (Claret Bioscience), following the manufacturer’s recommendations. The molar concentrations of the purified library pools were quantified using the Agilent 4150 TapeStation system with High Sensitivity D1000 ScreenTape. Final libraries were denatured and sequenced on the Illumina NextSeq 500/550 platform, following the manufacturer’s instructions, utilizing NextSeq High Output v2 flow cells and reagents. All libraries yielded sufficient read quality and diversity in their respective sequencing runs, ensuring that comparisons reflect true differences in library preparation performance rather than sequencing depth (Table S2).

### Sequence data processing and alignment

To ensure consistency and eliminate variability introduced by differing computational workflows, all sequencing data, regardless of the library preparation method, were processed using the Karius computational pipeline as previously described (13). Briefly, raw base call files were demultiplexed using *bcl2fastq* v2.20 with default settings. Read alignment to the human genome and synthetic control molecule references was performed using Bowtie 2 v2.2.4 (37). Microbial read classification was conducted using BLASTN v2.15.0 (38) against the proprietary Karius microbial genome reference database, followed by taxon abundance estimation using an expectation-maximization algorithm over the joint probability distribution of reads by taxa.

### Endogenous microbe detection and mcfDNA quantifications

For each plasma specimen, a list of detected microorganisms and their corresponding mcfDNA concentrations was generated for each of the four library preparation methods, as described previously (13). Briefly, microorganism calls were defined as microorganisms that are known human pathogens and commensal taxa present at statistically significant levels above background signals observed in EC samples. Additional quality filters were applied to account for read location uniformity, percent identity, and potential cross-reactivity from high-abundance taxa (13).

To determine whether each called microorganism originated from endogenous mcfDNA in the plasma rather than from environmental contamination (including reagents), an analytical framework was applied to distinguish endogenous from exogenous sources (Supplementary Text A; Fig. S2A and B). Microorganisms detected in the same plasma specimen by at least two distinct library preparation methods were classified as endogenously derived. For microorganisms detected by only a single method, the origin was resolved via a dilution-confirmation protocol: the plasma sample was serially diluted and reprocessed using the same library preparation method responsible for the initial call. The resulting mcfDNA concentrations were then assessed for dilution correlation. Microorganisms exhibiting a decrease in mcfDNA abundance proportional to plasma dilution were classified as endogenously derived; absence of such a trend indicated a likely exogenous origin.

### Measurement of mcfDNA yield

Total mcfDNA yield was quantified by sequencing close to saturation and counting unique reads and measuring their duplication rates (Supplementary Text B). To reduce costs, new libraries were generated by following the standard protocols for the pre-amplification steps. For amplification, however, 3% of the adapter ligation reaction was diluted to full volume with a freshly prepared 1× ligation buffer, then amplified with five additional PCR cycles. Libraries were pooled, sequenced, and analyzed as described. Final mcfDNA recovery was normalized to a fixed plasma volume (100 nL).

### Quantification of environmental contamination levels in Karius Helion-4

Libraries from 20 Karius Helion-4 EC buffer aliquots were prepared using standard Karius Helion-4 Chemistry and compared to 20 libraries generated without the proprietary contamination-reduction measures. All libraries were pooled, purified, sequenced, and analyzed to quantify microbial DNA from environmental contamination. Total contamination per library was calculated by summing species-level DNA concentrations, and contamination level was defined as the median total microbial DNA concentration across each set of 20 EC libraries.

### Interference assessment in Karius Helion-4

Interference testing followed CLSI EP07 and EP37 guidelines (Supplementary Text C) to assess assay performance in the presence of plasma interferents (proteins, hemolysate, lipids, and conjugated/unconjugated bilirubin) and K_2_EDTA. Briefly, pooled K_2_EDTA plasma from healthy donors (ZenBio, SER-PLP-1) was spiked with mcfDNA standard at 600 or 10,000 molecules/100 nL. Interference samples were prepared by adding each substance above its reference range to mcfDNA-containing plasma; matched controls used only the substance diluent. Samples were processed using Karius Helion-4, and libraries were generated, pooled, purified, and sequenced as described on the Illumina NextSeq 500/550 platform.

### Impact of pre-analytical sample handling on DNA concentration in plasma samples

Plasma samples collected in PPT and purple-top K_2_EDTA tubes from a healthy donor were spiked with the same mixture of control molecules as the test plasmas described above and in reference 13, then exposed to a daily shipping simulation cycle of 2 h at 37°C, 6 h at 4°C, and 16 h at room temperature that included 5 h of shaking at 150 RPM to simulate transit (Supplementary Text D). Sample aliquots were collected at time 0, 2 days, 4 days, 6 days, 8 days, and 10 days and frozen at −80°C before processing with Karius Helion-4. Libraries were then generated, pooled, purified, and sequenced as described on the Illumina NextSeq 500/550 platform.

### Statistical analysis

All statistical analyses were implemented in Python 3.10.6 using Scipy 1.15.3 library. Comparisons of clinical plasma performance for yield and enrichment across methods were done using paired Wilcoxon signed-rank tests on log-transformed data, which were designed for comparisons involving repeated measures on the same subject. Comparisons of methods for the ECs used an unpaired Mann-Whitney *U*-test, as the EC replicates were independently created for each method. Comparisons between the test and control replicates for the interference study were performed individually for each interferent using an unpaired Mann-Whitney *U*-test. Non-parametric tests were chosen to be robust to non-normal distributions (even after log-transformation) and to evaluate median fold-change differences. *P* values for all pairwise comparisons across the four methods were corrected with the Holm multiple comparisons correction.

## RESULTS

### Karius Helion-4 technology platform

The Helion-4 technology platform consists of two elements: Helion-4 Chemistry, an mcfDNA sequencing library preparation workflow common to multiple applications, including Karius Spectrum and Karius Focus | BAL infectious disease tests; and Helion-4 Compute, a suite of compute pipelines with each bioinformatics pipeline customized for a specific diagnostic or discovery application (Fig. 1A). Helion-4 Chemistry introduces several innovations that simplify library prep, including (i) ability to perform on automated liquid handling robots or manually in similar time; all experiments here used automation; (ii) internal control molecules that enable monitoring of process efficiency, unique sample tracking, AT/GC bias, and cross-contamination; (iii) an in-matrix chemistry that eliminates DNA extraction step and enables library prep directly in plasma and other body fluids, reducing time and minimizing mcfDNA losses and biases; and (iv) rigorous quality controls for fragment size and concentration prior to sequencing (Fig. 1B).

To assess the real-world performance of Helion-4 Chemistry, we measured the time required for each step as executed by clinical laboratory scientists in a CLIA/CAP/NYSDOH reference lab across multiple batch sizes (Fig. 1C). Timing began at the start of liquid handler setup, even prior to sample handling, and included all steps of the Helion-4 Chemistry protocol through completion of quality control assessments of final library pools. For a 24-sample batch, total processing time was 5.25 h with 2.25 h of hands-on time. For 96 samples, total time increased to 6.05 h, with hands-on time unchanged: only a 28% time increase despite a fourfold increase in batch size. Manual processing is faster for small batches but requires more hands-on effort. Including sequencing and Karius Helion-4 Compute, total end-to-end time ranged from 17.25 to 18.0 h. The protocol had a failure rate below 0.14% across 12,997 samples. The ability to complete library prep within one shift, integrated quality controls, and <24 h turnaround supports its utility for decentralized infectious disease applications.

### Defining a truth set for evaluation of comparator protocols

To assess the performance of the library preparation workflows in a clinically relevant context, 36 residual plasma samples from patients with suspected infectious disease were selected. Polymicrobial infections were prioritized to maximize the diversity of microbes contributing mcfDNA to the plasma (Fig. S1). Across all methods, 201 microbial species were detected, including 96 unique bacterial, viral, and fungal taxa (Table S3).

A key challenge in comparing clinical metagenomic protocols lies in the absence of a definitive ground truth in clinical specimens. Lacking a clinical ground truth, we developed an approach to classify each detected species as endogenous (plasma-derived) or exogenous (contaminant from reagents, surfaces, handling, etc.) (Supplementary Text A). Species detected by two or more methods were deemed endogenous, while those detected by one method were assessed via a replicate library and/or plasma dilution series (Fig. S2A and B; Supplementary Text E). Only species showing reproducible detection and proportional dilution response were considered endogenous. Of 201 total species, 198 were confirmed endogenous. Karius Helion-4 and Ext+dsDNA identified only endogenous microbes, while Ext+ssDNA reported one exogenous species, and Karius DC3 reported two exogenous species (Supplementary Text A; Table S3).

### Karius Helion-4 libraries exhibit higher recovery of endogenous mcfDNA

**Fig 2:**
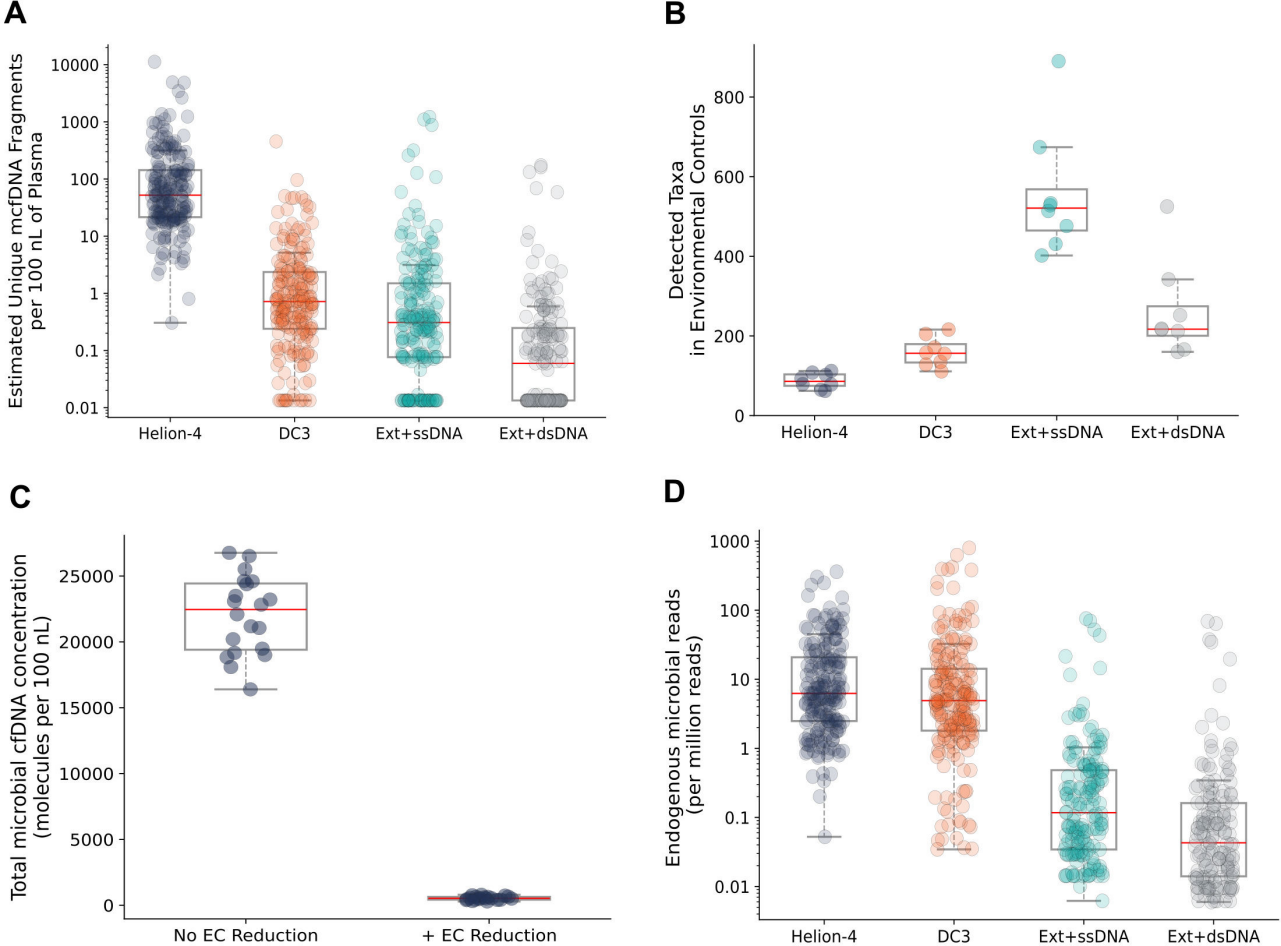
Assessment of Karius Helion-4 technical performance. (**A**) The number of unique mcfDNA fragments recovered per 100 nL of plasma by the Karius Helion-4, Karius DC3, and the two comparator methods. (**B**) The number of detected microbial taxa in the EC samples by the four methods. (**C**) Total concentration of the microbial DNA fragments in the ECs after processing with Karius Helion-4 or the version of Karius Helion-4 that lacked the contamination-reduction measures. (**D**) mcfDNA sequencing read frequency in the sequencing data obtained with the four methods.

mcfDNA is challenging to sequence due to low abundance and degraded nature (14, 26). To compare recovery efficiency across workflows, we quantified unique endogenous mcfDNA molecules in the final libraries using a volume-normalized, subsampled fraction of each adapter ligation reaction (Supplementary Text B). This conserved sequencing resources while enabling fair comparison of library complexity. Analyses were limited to endogenous mcfDNA reads and normalized to 100 nL plasma input volume. Karius Helion-4 yielded a median of 817-fold (*P* = 9.5E−7), 236-fold (*P* = 1.5E−7), and 58-fold (*P* = 2.2E−8) more endogenous mcfDNA than Ext+dsDNA, Ext+ssDNA, and Karius DC3, respectively (Fig. 2A).

### Karius Helion-4 minimizes signal from environmental contamination

In low-biomass samples like plasma, exogenous DNA can account for up to 99% of microbial reads (39, 40). To assess environmental contamination, we compared microbial taxa detected in ECs across methods (Fig. 2B). Helion-4 ECs contained a median of 86 taxa, compared to 217 in Ext+dsDNA (*P* = 0.006), 521 in Ext+ssDNA (*P* = 0.006), 2.5-fold and 6.0-fold higher, respectively, despite Helion-4’s superior microbial DNA recovery. Karius DC3 had a median of 156 taxa in the ECs (*P* = 0.006 vs Helion-4). Since Helion-4 and DC3 methods employ an EC matrix that recapitulates plasma properties more closely than IDTE, we also confirmed that Helion-4 showed similar decreases in IDTE-based ECs (Fig. S3A and B). Karius Helion-4 incorporates multiple layers of contaminant-reduction measures, the effectiveness of which was confirmed by comparing workflows with and without these measures. ECs processed with these measures showed a 42-fold lower total microbial DNA concentration than without them (*P* = 6.8E−8, Fig. 2C), underscoring Helion-4’s robust contamination reduction.

### Karius Helion-4 enrichment for mcfDNA boosts sensitivity

Because mcfDNA typically constitutes <0.001% of total cfDNA (13, 41), enrichment is essential for scalable clinical metagenomics. Helion-4 integrates mcfDNA enrichment steps directly into the library workflow without added time, cost, or complexity. In 36 plasma libraries, Helion-4 yielded a median of 164-fold (*P* = 4.4E−8) and 60-fold (*P* = 4.4E−8) higher fraction of endogenous mcfDNA reads compared to Ext+dsDNA and Ext+ssDNA, respectively (Fig. 2D), demonstrating highly effective enrichment. Helion-4 did not show significantly higher enrichment than Karius DC3 (1.02-fold, *P* = 0.93).

### Absolute mcfDNA quantification

**Fig 3:**
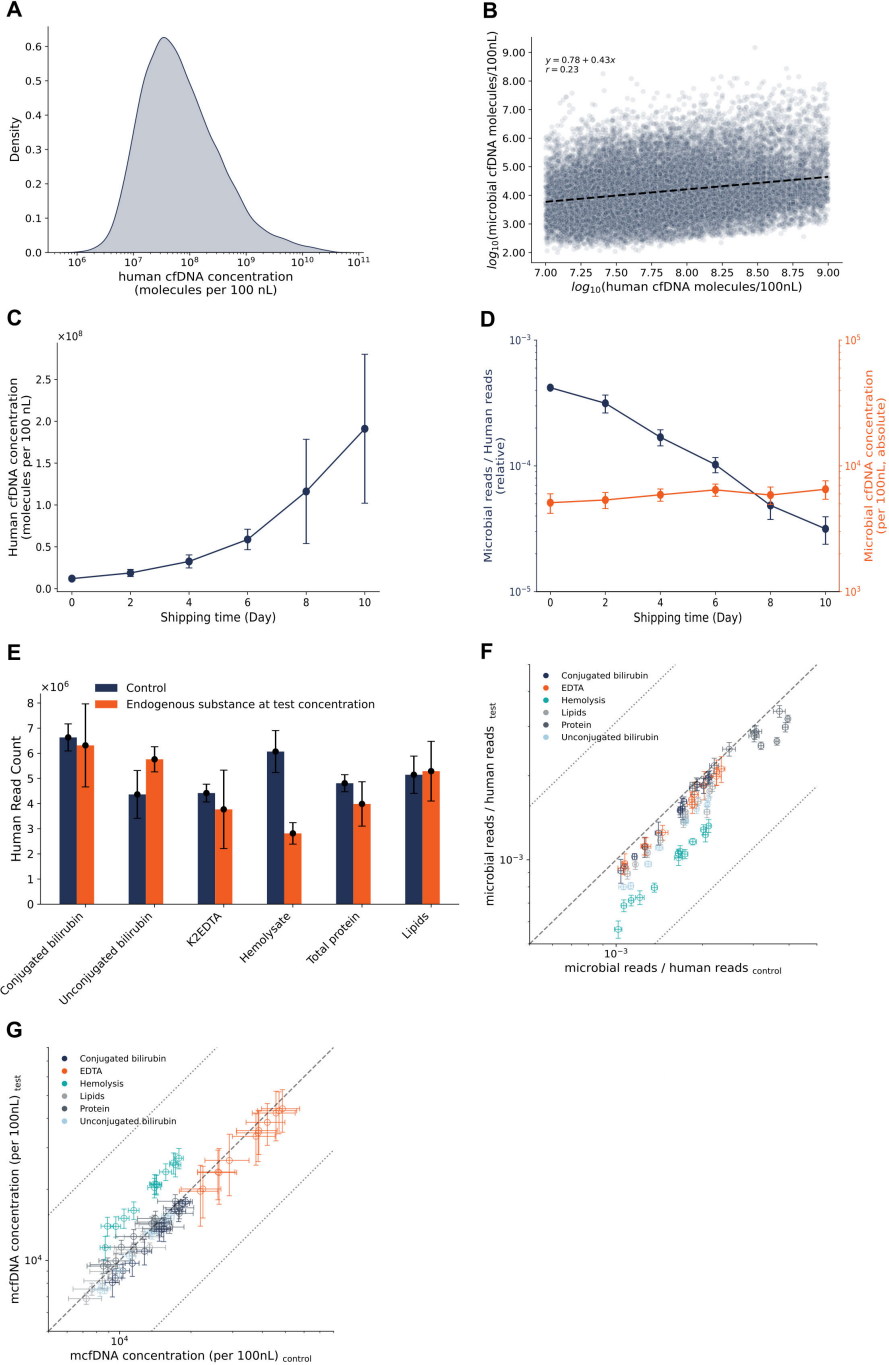
Karius Helion-4 technical performance as compared to the comparative methods. (**A**) The distribution of hcfDNA concentration in plasma as measured in the commercial plasma samples processed by Karius Helion-4 in this study (*N* = 19,075). (**B**) mcfDNA concentration correlation with hcfDNA concentration in the plasma sample set in panel **A**. (**C**) hcfDNA concentration in a plasma sample during a shipping simulation study (Supplementary Text D). (**D**) A comparison of the absolute mcfDNA concentration by Karius Helion-4 and relative quantification with the hcfDNA background in the plasma sample during a shipping simulation (Supplementary Text D). (**E**) The effect of the common plasma endogenous substances and K_2_EDTA anticoagulant on the recovery of human reads of Karius Helion-4 as measured by the interference study (Supplementary Text C). (**F**) The effect of common plasma endogenous substances and K_2_EDTA as an anticoagulant on the frequency of mcfDNA-derived reads in the sequencing data as measured by the interference study (Supplementary Text C). (**G**) The effect of the common plasma endogenous substances and K_2_EDTA anticoagulant on the Karius Helion-4 absolute mcfDNA concentration as measured by the interference study (Supplementary Text C).

Accurate cfDNA quantification is important for clinical interpretation and monitoring. While mcfDNA is often reported relative to hcfDNA, this may be unreliable if hcfDNA varies independently. In over 2,500 clinical specimens, hcfDNA spanned >4 log total range (Fig. 3A), with poor correlation to mcfDNA levels (Pearson’s *ρ* = 0.23, Fig. 3B). In addition, pre-analytical handling conditions can also influence hcfDNA concentration independent of mcfDNA. For example, a simulated 10-day shipping increased hcfDNA up to 17-fold, without corresponding mcfDNA change (Fig. 3C; Supplementary Text D). To mitigate the imprecision caused by independently variable hcfDNA variations, Helion-4 uses an internal-control-based absolute quantification method, independent of hcfDNA and sequencing depth, enabling reproducible measurement (13). The difference in approaches is readily observed by noting that the extended shipping conditions resulted in a 13-fold decrease in relative mcfDNA concentration when normalized to hcfDNA, while the absolute mcfDNA concentration itself changed only 1.27-fold (Fig. 3D).

### Karius Helion-4 is not significantly impacted by common interferents in plasma

The elimination of the DNA extraction step in the Helion-4 technology platform provides several advantages; however, it also raises the potential risk of interference from endogenous plasma components such as proteins, lipids, conjugated and unconjugated bilirubin, and hemolysate, and exogenous substances like anticoagulants used during sample collection (e.g., K₂EDTA). To assess the potential interference of these substances with Helion-4, we systematically measured their effect on the yield and mcfDNA concentrations (Fig. 3E through G; Supplementary Text C). All but hemolysate (*P* = 0.0021) did not show statistically different human read count and mcfDNA concentrations between the control and the test sample, though the test sample with unconjugated bilirubin showed a marginally significant increase in human read count (*P* = 0.055). All of the tested substances did decrease the frequency of mcfDNA reads by 10.9%–36.3% (*P* < 0.0032). Meanwhile, tested substances met the acceptance criteria related to mcfDNA concentration for all microbial species at both 2× and 30× LoD of microbial detection (Supplementary Text C). For qPCR assays in microbiology laboratories, concentration changes <0.5 log are not considered medically significant. In line with this, previous sequencing studies (42–45) have reported that log changes in mcfDNA concentration are considered significant. In this context, the mcfDNA concentration results from the interference examination study (Fig. S4A through F) demonstrated that none of the tested substances interfered with Karius Helion-4 performance to a significant degree.

### Karius Helion-4 reveals more mcfDNA than comparative approaches

Out of 36 plasma samples obtained from the patients with suspected infection, endogenous microbes were called in 28 plasma samples when the Karius Helion-4 was applied, compared to 4 samples in the case of Ext+dsDNA, 8 in Ext+ssDNA, and 25 in Karius DC3. In total, 198 unique endogenous microbe calls were detected in 36 plasma samples cumulatively by all four approaches (Table S3). Karius Helion-4 called 83.3% (165/198) of all the unique endogenous microbes. In contrast, only 6 (3.0%) of these were detected by Ext+dsDNA and 12 (6.0%) by Ext+ssDNA. By comparison, Karius DC3 called 78.3% (155/198) of the unique endogenous microbes, sharing 61.6% (122/198) with Karius Helion-4. No unique endogenous microbes were detected exclusively by either of the extraction-based methods.

## DISCUSSION

This study introduces the Karius Helion-4 in-matrix library preparation technology, a novel solution designed to overcome barriers to widespread clinical adoption of mcfDNA sequencing, particularly in decentralized settings where operational simplicity and minimal infrastructure are critical. A key challenge in such contexts is the complexity and duration of sequencing preparation workflows, especially for urgent clinical applications like infectious disease diagnostics. Karius Helion-4 addresses this by enabling single-shift library preparation with just 2.25 h hands-on time and ~6 h total processing, regardless of batch size. This encompasses all steps from setup to library QC, allowing true end-to-end sample preparation within a standard workday.

By eliminating DNA extraction, Helion-4 Chemistry reduces complexity and infrastructure demands, thus lowering barriers for institutions without high-throughput capabilities and shortening turnaround time for time-sensitive cases like febrile neutropenia or sepsis. Performing all reactions directly in plasma preserves short and fragmented microbial DNA typically lost during extraction, yielding >100-fold more endogenous mcfDNA than common workflows (Fig. 2A) and >50-fold higher mcfDNA read fraction (Fig. 2D). This improves analytical sensitivity and reduces sequencing costs. Remarkably, despite the higher microbial yield, Karius Helion-4 showed the lowest environmental contamination, attributable to the reduced number of reagents, proprietary contamination-reduction methods, and real-time contamination monitoring. This is vital for low-biomass samples like plasma, where contaminants can obscure biological signals and lead to false positive signals. Additionally, the use of internal controls for absolute mcfDNA quantification avoids pitfalls of normalization to variable host cfDNA, enabling accurate, reproducible quantitative microbial measurements across time points and specimens.

While high sensitivity enables robust detection of endogenous microbial DNA, not all endogenous microbes are relevant for a particular assay application. For example, an assay focusing on the detection of infectious disease targets known pathogens, whereas an assay that reports biomarkers of gut health would target known gut commensal microbes. To enhance the interpretability of the Helion-4 technology platform, Helion-4 Compute pipelines are modified with application-specific filters. In particular, Karius Spectrum includes a literature-based filter for pathogens with established disease associations and a reference interval filter derived from 463 asymptomatic donors to exclude organisms present within the bounds of normal variation. When applied to this data set, Karius Spectrum filters reduced the total endogenous reported taxa of the Helion-4 processed samples from 165 to 88 and median reportable taxa per sample from 6 to 3 (Table S3), providing a focused view into potential infectious pathogens in this cohort enriched for suspected polymicrobial infection. Thus, different applications can be built on the foundation of the Helion-4 technology platform, leveraging computational filters on top of high sensitivity for mcfDNA to tune for distinct clinical needs.

While promising results are described in the current study, limitations exist. The comparative evaluation here used a relatively small sample set (*n* = 36) and focused only on plasma. A broader investigation is needed across sample types (e.g., urine, CSF, BAL), diverse clinical contexts, and larger cohorts. Moreover, without a gold-standard reference, we cannot definitively assess false-positive or false-negative rates for any method tested.

Karius Helion-4 technology represents a pivotal advancement for mcfDNA metagenomics. By reducing sample preparation complexity, time, and infrastructure requirements of library preparation, Helion-4 brings us closer to widespread, high-resolution, and real-time microbial profiling across diverse healthcare settings. Its combination of quantitative precision, reduced environmental contamination, and data-tuned frameworks for interpretability directly addresses persistent challenges in clinical metagenomics. As validation efforts expand and the exploration of applications across varied sample types and disease contexts widens, Helion-4 holds promise not only as a diagnostic tool but also as a foundation for the next generation of precision medicine, where microbial insights are integral to the care of every patient, everywhere.

## SUPPLEMENTAL MATERIAL

Supplemental materials jcm.00944-25-s0001.docx Supplemental text, Tables S1 to S6, and Figures S1 to S7.

ASM does not own the copyrights to Supplemental Material that may be linked to, or accessed through, an article. The authors have granted ASM a non-exclusive, world-wide license to publish the Supplemental Material files. Please contact the corresponding author directly for reuse.
