# Supplementary material for: In-matrix library preparation for metagenomic sequencing of microbial cell-free DNA
Source: J Clin Microbiol. 2025 Nov 28;63(12):e00944-25. doi: 10.1128/jcm.00944-25 (PMC12710328; doi:10.1128/jcm.00944-25)
Supplement: Supplemental materials — Supplemental text, Tables S1 to S6, and Figures S1 to S7. [file jcm.00944-25-s0001.docx]

**In-Matrix Library Preparation for Metagenomic Sequencing of Microbial Cell-Free DNA**

**SUPPLEMENTARY MATERIAL**

Paul L. Babb^a^, Jamilla Akhund-Zade^a^, Damek Spacek^a^, Kevin Brick^a^, Fred C. Christians^a^, Victoria Portnoy^a^, Ming-Shian Tsai^a^, Kristin H. Jarman^a^, Sivan Bercovici^a^, Igor D. Vilfan^a^, Timothy A. Blauwkamp^a*^

^a^ Karius, Inc.

*Address correspondence to this author at 975 Island Drive, Redwood City, CA 94065, USA. Email [tim.blauwkamp@kariusdx.com](mailto:tim.blauwkamp@karius.dx.com)

# Supplementary Text

## **Supplementary Text A.**

*IDENTIFICATION OF THE ENDOGENOUS MICROBIAL SPECIES IN THE LIST OF INITIALLY CALLED MICROBES*

The initial list of microbial species was generated after applying uniformity and pathogen cross-reactivity filters, as described in **Materials and Methods**. This list included microbial species that were endogenously present in the original plasma sample but may have also contained those introduced via sporadic contamination events during sample processing prior to adapter ligation. Such contaminants could arise from environmental sources, including dust particles or randomly contaminated laboratory consumables (e.g., pipette tips, plate wells). Given the stochastic nature of such contamination events, external environmental control samples, as used in this and similar studies, are insufficient for their reliable detection, since the probability of both plasma and control samples acquiring the same sporadic contaminant is low.

To accurately evaluate the performance of the four sequencing library preparation methods, it was essential to distinguish endogenous microbial species from those introduced via sporadic environmental contamination. The origin of each initially called microbe was determined using an algorithm schematically outlined in **Supplementary Figure 2A**. A comprehensive list of initially called microbial species, categorized by their originating plasma sample across all four laboratory processes, is provided in **Supplementary Table 3**. Microbial species detected independently by at least two laboratory processes are highlighted in bold, while those identified by a single process are presented in regular font.

Microbial species detected in the same plasma sample by at least two independent methods were classified as endogenous. This assumption is based on the low probability that two independently performed sequencing protocols using distinct reagents would generate identical sporadic contaminations that were undetectable by the environmental control samples. For microbial species initially detected by only a single method, endogenous status was adjudicated based on sequencing results from a dilution series of the corresponding plasma sample (**Supplementary Figure 2A, Supplementary Text E**).

To label a uniquely detected microbial call as endogenous, it had to be detected in a replicate of the originally processed plasma sample that was not diluted (e.g. the original commercial sample or the undiluted library in the dilution series), provided that sequencing depths of the replicate libraries and the original plasma aliquot were comparable. The probability of failing to detect an endogenous microbe in the undiluted replicate was required to be <5%. Sequencing depth analysis revealed that libraries from the undiluted members of the dilution series had, on average, a two-fold higher sequencing depth than those derived from the original replicate (**Supplementary Figure 5**). Under such conditions, if a microbial species were endogenous, the probability of its complete absence in the undiluted replicate was approximately zero (<2E-9).

Additionally, the absolute abundance of an endogenous microbial species in plasma is expected to be inversely proportional to the dilution factor **(Supplementary Figure 2B)**. This expectation was used to compute the log-likelihood ratio comparing the probability of the microbial species being endogenous versus originating from the diluent or the reagents.

Of the 201 initially called microbial species, 12 (6.0%) were shared between Karius Helion-4 and either the Ext+dsDNA or Ext+ssDNA approaches. 122 (60.7%) were shared between Karius Helion-4 and DC3. No unique microbial species were identified by Ext+dsDNA. 42 were unique to Helion-4, 35 were unique to DC3, and 1 was unique to Ext+ssDNA. The microbes exclusively called in just one chemistry were, based on the algorithm in **Supplementary Figure 2A**, classified as endogenous to their respective plasma samples.

A unique microbial species, *Bacillus pumilus*, was identified exclusively by the Ext+ssDNA approach in plasma_026. However, when subjected to the dilution series analysis, the undiluted plasma_026 sample failed to replicate the *Bacillus pumilus* call, nor was it observed in diluted samples. The probability of an endogenous microbe not being observed in the undiluted replicate remained approximately zero (<2E-9) given the sequencing depth conditions between the original sequencing and sequencing of the undiluted replicate (**Supplementary Figure 5**). Furthermore, the expected inverse proportionality between microbial cell-free DNA (mcfDNA) concentration and the dilution factor was used to compute the log-likelihood ratio of *Bacillus pumilus* being endogenous versus a sporadic contaminant. This analysis indicated an 11-fold higher likelihood of *Bacillus pumilus* originating from reagent background, leading to its exclusion from further analysis.

Thirty-five (35, 17.4%) microbial species were called exclusively by Karius DC3. Of these microbial species, only two found in plasma_026, *Kytococcus sedentarius* and *Micrococcus lylae,* failed to replicate in the original commercial sample processed with DC3. The probability of these microbes not being observed in the more deeply sequenced commercial sample is approximately zero (<2E-9). In addition, they were not observed in the paired Karius Helion-4 samples. Therefore, we excluded these microbes as potential sporadic contaminants. The other plasma samples with unique microbial calls underwent a dilution series analysis as outlined in **Supplementary Figure 2.** 33 microbial species were classified as endogenous based on the criteria of the algorithm in **Supplementary Figure 2A**: (1) their detection in an undiluted replicate library and (2) their mcfDNA concentration is inversely proportional to the dilution factor.

A total of 42 (20.8%) microbial species were called exclusively by Karius Helion-4. Their respective plasma samples also underwent a dilution series analysis as outlined in **Supplementary Figure 2**. All microbial species uniquely reported by Karius Helion-4 were classified as endogenous based on the criteria of the algorithm in **Supplementary Figure 2A**, mentioned above.

## **Supplementary Text B.**

*ESTIMATING UNIQUE MCFDNA CONTENT OF A LIBRARY FOR YIELD CALCULATIONS*

For the following yield calculations, we used “saturation PCR” libraries, where 3% of the original ligation volume was amplified using 5 additional PCR cycles. The “saturation PCR” libraries were chosen for yield calculations so that the taxa would have a low unique fraction of the detected sequencing reads in order to generate reliable yield estimates with the method outlined below. The yield estimates reported in the manuscript reflect the “saturation PCR” libraries.

To estimate the unique mcfDNA content of a library per taxon (N), we input the observed count of unique reads (n) and observed count of total reads (m) attributed to a specific species into a Poisson-Gamma model to get the maximum a posteriori for the Poisson parameter λ_MAP_, or rate of being sequenced e.g., λ_MAP_ = m/N.

$$P(\lambda\vee n,m) \Gamma(\alpha+m,\beta+n)$$

$$\frac{d}{d\lambda}P(\lambda\vee n,m)=e^{-\lambda}+\frac{\beta+n}{\alpha+m-1}\lambda-1$$

$0=e^{-\lambda_{MAP}}+\frac{\beta+n}{\alpha+m-1}\lambda_{MAP}-1$

Where 𝛼 = 1 and 𝛽 = 0 are the parameters of the Gamma prior distribution.

Using the known relationship between n, λ_MAP_ and the “unobserved” fraction of the taxon mcfDNA molecules (n_0_), i.e., the fraction of mcfDNA molecules that was not sequenced, we can calculate the maximum a posteriori for the unique mcfDNA content of the taxon or N_MAP_.

$n=N-n_{0}$

$$n=N-Ne^{-\lambda}$$

$$N_{MAP}=\frac{n}{1-e^{-\lambda_{MAP}}}$$

If n = m, which was often the case when a taxon was in low abundance, this estimation procedure would fail. In those cases, we estimated m from the overall microbial unique fraction of the sample (f), m = n/f. The overall microbial unique fraction is calculated as f = n_total_/m_total_, where n_total_ is the count of all unique microbial reads in the sample and m_total_ is the count of all microbial reads in the sample.

The above method assumes that each unique mcfDNA molecule has an equal probability of being sampled (1/N), which does not take into account potential PCR biases during indexing PCR that could preferentially amplify certain molecules more than others. Heterogeneity in sampling probability could decrease the final estimate of the unique mcfDNA content, but the effect should be consistent across the different protocols as they all share the same indexing PCR reaction.

## **Supplementary Text C.**

*ASSESSING INTERFERENCE EFFECTS OF THE ENDOGENOUS AND EXOGENOUS PLASMA SUBSTANCES IN KARIUS HELION-4*

**Study Design**

To assess the impact of relevant endogenous human plasma substances and the anticoagulant K₂EDTA on the performance of Karius Helion-4, we conducted an interference study following Clinical and Laboratory Standards Institute (CLSI) guidelines EP07 (*Interference Testing in Clinical Chemistry*) and EP37 (*Supplemental Tables for Interference Testing in Clinical Chemistry*). The substances evaluated for potential interference included total plasma proteins, hemolysate, lipids, conjugated bilirubin, and unconjugated bilirubin. Additionally, K₂EDTA, an anticoagulant commonly used in plasma collection tubes (e.g., PPT and purple-top tubes), was tested for its potential effects on assay performance.

**Plasma Matrix Selection**

In accordance with CLSI EP07 recommendations, pooled healthy K₂EDTA plasma (ZenBio, Cat. SER-PLP-1) was used as the background matrix for assessing interference effects. To evaluate the impact of K₂EDTA, blood from a healthy donor was collected into three plasma preparation tubes (PPTs; BD Vacutainer™, Cat. 362788) and transported to Karius headquarters within 6 hours of collection. Upon arrival, plasma was separated by centrifugation at 1,100 x g for 10 minutes at 25°C. Before preparing interference test samples, baseline concentrations of selected interferents in the pooled K₂EDTA plasma were measured to ensure that interference was assessed at concentrations appropriate for clinical relevance, as specified in CLSI EP37.

**Measurand Preparation**

Following CLSI EP07 guidelines, interference was evaluated using contrived samples prepared by spiking mcfDNA standards into the plasma matrix (1). A mixture of 13 microbial species (P13 mix, **Supplementary Table 4**) was spiked into pooled K₂EDTA plasma at 2X and 30X the limit of detection (LoD), with each concentration prepared in sufficient volume to generate 14 replicates per tested interferent.

**Interfering Substances**

To ensure comprehensive interference testing, we reviewed endogenous interfering substances previously evaluated in FDA 510(k)-cleared devices utilizing plasma-based input samples and PCR/sequencing-based DNA detection for cancer and infectious disease diagnostics (**Supplementary Table 5**). Based on this review, we selected total plasma proteins, triglyceride-rich lipoproteins, hemolysate, conjugated bilirubin, and unconjugated bilirubin as endogenous substances for evaluation. Additionally, K₂EDTA was included as an exogenous interferent, given its use as the anticoagulant in Karius Spectrum assays employing Karius Helion-4. Standards for all interfering substances were obtained from Sun Diagnostics (ME, USA), and test concentrations were established in accordance with CLSI EP37 recommendations (Table 2 in CLSI EP37). The recommended K₂EDTA test concentration was set at three times the expected additive concentration (CLSI EP07, Section 3.4.3). Assuming complete separation of K₂EDTA (9 mg per 5 mL PPT tube) into the plasma fraction, and given that plasma constitutes approximately 55% of total blood volume, the estimated plasma K₂EDTA concentration from 5 mL of whole blood was 8.1 mM. Thus, the recommended test concentration was 24.3 mM (~25 mM). To simulate the most stringent conditions, the K₂EDTA concentration in this study was set to at least 25 mM.

**Sample Preparation**

The P13 mix (*see Measurand section*) was spiked into pooled healthy K₂EDTA plasma at 2X and 30X LoD, with sufficient volume prepared for 14 replicates per tested interferent. Test samples were prepared by adding an interferent stock solution to aliquots of the base pool containing the measurand to achieve the target interferent concentrations. Control samples were prepared by adding an equivalent volume of the solvent used to dissolve the interferent. Additionally, interferent standard blanks (two replicates per interferent) were prepared by adding interferent stock solutions to Karius EC buffer to screen for potential microbial contamination in the interferent stock that could affect the mcfDNA concentration of P13 microbial species.

**Sample Processing**

All prepared samples were processed using Karius Helion-4 as described in the **Materials and Methods** section.

**Allowable Total Error, Acceptance Criteria for mcfDNA concentration measurements, and Sample Size**

Acceptance criteria were based on allowable total error (ATE) goals for the assay, following CLSI EP21 guidelines. ATE is defined as the analytical quality goal that sets limits for both imprecision (random error) and bias (systematic error) in a single measurement. The ATE for clinical samples was determined by reviewing the Karius Spectrum literature for medical decision points (**Supplementary Table 1**). For qPCR assays in microbiology laboratories, changes less than 0.5 log are not considered medically significant. In line with this, previous studies (2-5) have reported that log changes in mcfDNA concentration are considered significant. Therefore, we set the ATE for this study at a 0.5-log (3.16-fold) change in measurand concentration. Considering the all-sources coefficient of variation (CV) of 23% and repeatability CV of 14.4% for Karius Helion-4, and adjusting for uncertainty in concentration estimates of both test and control samples, the acceptance criterion for the 95% confidence interval around paired differences was set to be fully contained within the range (-63.3, 211).

**Results**

All tested substances met the acceptance criteria related to mcfDNA concentration for all microbial species at both 2X and 30X LoD. Thus, none of the evaluated endogenous or exogenous substances were determined to interfere with Karius Helion-4 (**Supplementary Table 6**). **Supplementary Figure 4** displays confidence intervals for relative differences associated with each tested interfering substance, including:

- Total plasma proteins (**Supplementary Figure 4A**),
- Hemolysate (**Supplementary Figure 4B**),
- Lipids (**Supplementary Figure 4C**),
- Conjugated bilirubin (**Supplementary Figure 4D**),
- Unconjugated bilirubin (**Supplementary Figure 4E**),
- K₂EDTA (**Supplementary Figure 4F**).

The acceptance criterion range (-63.3, 211) is indicated by horizontal black lines on each plot. During testing, certain microbial species from the P13 mix were detected in the interferent stock solutions at statistically significant levels. These taxa, listed in **Supplementary Table 7**, were removed from the final analysis to avoid confounding effects.

**Supplementary Text C References**

1. Blauwkamp TA, Thair S, Rosen MJ, Blair L, Lindner MS, Vilfan ID, Kawli T, Christians FC, Venkatasubrahmanyam S, Wall GD, Cheung A, Rogers ZN, Meshulam-Simon G, Huijse L, Balakrishnan S, Quinn JV, Hollemon D, Hong DK, Vaughn ML, Kertesz M, Bercovici S, Wilber JC, Yang S. 2019. Analytical and clinical validation of a microbial cell-free DNA sequencing test for infectious disease. Nat Microbiol 4:663–674.
2. Yang H, Haidar G, Al-Yousif NS, Zia H, Kotok D, Ahmed AA, Blair L, Dalai S, Bercovici S, Ho C, McVerry BJH, Morris A, Kitsios GD. 2021. Circulating microbial cell-free DNA is associated with inflammatory host-responses in severe pneumonia. Thorax 76:1231-1235.
3. Kitsios GD, Bain W, Al-Yousif N, Duttagupta R, Ahmed AA, McVerry BJ, Morris A. 2021. Respir Res 22:24.
4. To RK, Ramchandar N, Gupta A, Pong A, Cannavino C, Foley J, Farnaes L, Coufal NG. 2021. Use of Plasma Metagenomic Next-generation Sequencing for Pathogen Identification in Pediatric Endocarditis. Pediatr Infect Dis J 40:486–488.
5. Lee RA, Dhaheri FA, Pollock NR, Sharma TS. 2020. Assessment of the Clinical Utility of Plasma Metagenomic Next-Generation Sequencing in a Pediatric Hospital Population. J Clin Microbiol 58:10.

## **Supplementary Text D.**

*THE EFFECT OF PRE-ANALYTICAL HANDLING OF PLASMA SAMPLES ON THE DNA CONTENT OF THE SAMPLES*

To assess the impact of pre-analytical handling conditions commonly encountered during plasma sample transport prior to assay processing, we conducted a study to evaluate the effects of typical shipping conditions on the DNA content of plasma samples and mcfDNA quantification. These conditions included temperature fluctuations, agitation, and shipping duration.

**Sample Collection and Plasma Preparation**

Blood draws were performed by Cureline, Inc. at 7:10 AM into 5 mL plasma preparation tubes (PPTs) and 4 mL K₂EDTA blood collection tubes from a single healthy donor. Samples were immediately transported at ambient temperature to Karius headquarters, where the mcfDNA standard was added at 8:30 AM. Plasma separation was performed at 1:00 PM on the day of sample receipt according to manufacturer recommendations. For PPTs, centrifugation was conducted at 1,100 x g for 10 minutes, yielding approximately 2.5 mL of plasma per tube, as estimated by the height of the plasma column above the gel. Plasma remained in the PPT tubes throughout the study duration. K₂EDTA blood collection tubes were centrifuged at 1,200 x g for 10 minutes, producing approximately 2 mL of plasma per tube. Plasma from K₂EDTA tubes was pooled into a 50 mL conical tube, and 1 mL aliquots were distributed into sterile polypropylene tubes.

**Storage and Shipping Simulation Conditions**

Baseline control samples (time-zero controls) were immediately frozen at -80°C until processing with Karius Helion-4 and sequencing, as described in the **Materials and Methods** section. The remaining plasma samples were subjected to a simulated shipping cycle that included: 2 hours at 37°C, 6 hours at 4°C, and 16 hours at room temperature, including 5 hours of shaking at 150 RPM. Plasma aliquots were collected from each sample tube at 48-hour intervals and stored at -80°C until processing with Karius Helion-4, sequencing and analysis, following the procedures detailed in the **Materials and Methods** section.

## **Supplementary Text E.**

*DILUTION SERIES METHOD, LIBRARY PREPARATION AND DATA ANALYSIS*

A serial dilution approach can be employed to differentiate microbial species endogenous to a plasma sample, i.e. originating from the mcfDNA signal, from those introduced by processing reagents or sporadic environmental contamination. This method relies on the correlation between microbial DNA fragment abundance and the plasma dilution factor. Specifically, the abundance of microbial species that are endogenous to plasma is expected to be inversely proportional to the dilution factor, following the equation (Supplementary Figure 2B):

$$c_{diluted}=\frac{c_{undiluted}}{DF}$$

where *c_diluted_* is mcfDNA concentration in the diluted plasma samples, *c_undiluted_* is the mcfDNA concentration in the undiluted plasma samples, and *DF* is the dilution factor defined as:

$$DF=\frac{V_{plasma}+V_{diluent}}{V_{plasma}}$$

where *V_plasma_* and *V_diluent_* are the volume of undiluted plasma and diluent used to prepare the diluted plasma aliquot, respectively. Conversely, the abundance of the microbial species that is contributed by the diluent will increase with the dilution factor (Supplementary Figure 2B):

$$c_{diluted}=c_{undiluted}\cdot\frac{(DF-1)}{DF}$$

where *c_undiluted_* is now the microbial fragment concentration in the pure diluent. Furthermore, the abundance of the microbial species that is contributed by the environmental contamination associated with the library preparation process (e.g. extraction and library reagents, and consumables) will be independent of the dilution factor (Supplementary Figure 2B):

$$c_{diluted}=c_{undiluted}$$

where *c_undiluted_* is mcfDNA concentration in the undiluted plasma samples. Finally, microbial species can be classified as sporadic contaminants if they are absent in all undiluted sample library replicates while sequencing was performed at equal depths. Similarly, a microbial species can be considered a sporadic contaminant if it is not detected in the diluted sample library, provided that sequencing depths are equivalent between the diluted and undiluted samples and that the microbial abundance in the undiluted sample is above the limit of detection (LoD) for the method.

**Experimental Procedure for Serial Dilution Analysis**

Plasma serial dilutions in this study were prepared by generating a dilution series consisting of both undiluted and diluted plasma aliquots. Diluted plasma aliquots were prepared through sequential 2-fold dilutions to generate 2X, 4X, 8X, and 16X diluted samples using 1X TET buffer. Each dilution series member was spiked with control molecules and processed according to the respective method described in **Materials and Methods**, with the exception that no splitting of the ligation reaction into separate fractions was performed prior to amplification. The resulting sequencing pools were purified and sequenced following the standard procedures outlined in **Materials and Methods**.

**Data Analysis and Microbial Classification**

Sequencing data processing, read alignment, microbial species detection, and quantification were conducted as detailed in **Materials and Methods**. Given that the absolute abundance of an endogenous microbial species is expected to decrease proportionally with increasing dilution, we computed the log-likelihood ratio to determine whether a detected microbe originated endogenously from plasma or was introduced through environmental contamination during the library preparation process. This analysis generated an initial list of microbial species likely to be endogenous to the sample. In addition, we confirmed via a log-log plot that the abundance of the microbe is decreasing with increasing dilution.

# Supplementary Tables

**Supplementary Table 1.**

Literature survey of peer-reviewed metagenomic sequencing studies utilizing the mcfDNA analyte.

| **Assay**  **Type** | **First**  **Author** | **Year** | **Journal** | **Title** | **DNA**  **Extraction** | **Library**  **Preparation** |
| --- | --- | --- | --- | --- | --- | --- |
| **RUO** | Barsan | 2022 | *Science Advances* | Simultaneous monitoring of disease and microbe dynamics through plasma DNA sequencing in pediatric patients with acute lymphoblastic leukemia | Promega Maxwell RSC cfDNA Plasma Kit (bead-S) | NEBNext UltraII DNA Library Prep Kit (ds) |
| **RUO** | Burnham | 2016 | *Scientific Reports* | Single-stranded DNA library preparation uncovers the origin and diversity of ultrashort cell-free DNA in plasma | Qiagen QIAamp Circulating Nucleic Acid Kit (col) | Homebrew (ss/ds) |
| **RUO** | Burnham | 2018 | *Nature Communications* | Urinary cell-free DNA is a versatile analyte for monitoring infections of the urinary tract | Qiagen QIAamp Circulating Nucleic Acid Kit (col) | Homebrew (ss/ds) |
| **RUO** | Burnham | 2020 | *Microbiome* | Separating the signal from the noise in metagenomic cell-free DNA sequencing | Qiagen QIAamp Circulating Nucleic Acid Kit (col) | Homebrew (ss/ds) |
| **RUO** | Chang * | 2021 | *Clinical Chemistry* | Measurement Biases Distort Cell-Free DNA Fragmentation Profiles and Define the Sensitivity of Metagenomic Cell-Free DNA Sequencing Assays | Qiagen QIAamp Circulating Nucleic Acid Kit (x3) (col);  Norgen Urine Cell-Free Circulating DNA Purification Kit (col);  Qiagen QIAquick Nucleotide Removal Kit (col);  Thermo Fisher MagMAX Cell-Free DNA Isolation Kit (x2) (bead) | Homebrew (x4) (ss/ds);  NEBNext UltraII DNA Library Prep Kit (ds)  Claret Bio SRSLY (ss/ds) |
| **RUO** | Cheng | 2019 | *Systems Biology* | A cell-free DNA metagenomic sequencing assay that integrates the host injury response to infection | Qiagen QIAamp Circulating Nucleic Acid Kit (col) | Homebrew (ss/ds) |
| **RUO** | De Vlaminck | 2015 | *PNAS* | Noninvasive monitoring of infection and rejection after lung transplantation | Qiagen QIAamp Circulating Nucleic Acid Kit (col) | NEBNext UltraII DNA Library Prep Kit (ds) |
| **RUO** | Fan ​​^†^ | 2008 | *PNAS* | Noninvasive diagnosis of fetal aneuploidy by shotgun sequencing DNA from maternal blood | Qiagen QIAamp DNA Micro Kit (col);  Macherey–Nagel NucleoSpin Plasma Kit (col) | Solexa/Illumina-based blunt end ligation (ds) |
| **RUO** | Kowarsky ^‡^ | 2017 | *PNAS* | Numerous uncharacterized and highly divergent microbes which colonize humans are revealed by circulating cell-free DNA | Qiagen QIAamp DNA Micro Kit (col);  Macherey–Nagel NucleoSpin Plasma Kit (col) | NEBNext UltraII DNA Library Prep Kit (ds) |
| **RUO** | Li | 2022 | *Frontiers in Microbiology* | Metagenomic Next-Generation Sequencing for the Microbiological Diagnosis of Abdominal Sepsis Patients | PathoXtract Plasma Nucleic Acid Kit (col) | KAPA DNA HyperPrep Kit (ds) |
| **RUO** | Sam | 2021 | *The Journal of Molecular Diagnostics* | Evaluation of a Next-Generation Sequencing Metagenomics Assay to Detect and Quantify DNA Viruses in Plasma from Transplant Recipients | Qiagen EZ1 Advanced XL system + EZ1 DSP Virus Kit (bead-S) | Arc Bio xGen DNA Library Prep Kit (ds) |
| **RUO** | Sun | 2022 | *Infection and Drug Resistance* | A Paired Comparison of Plasma and Bronchoalveolar  Lavage Fluid for Metagenomic Next-Generation  Sequencing in Critically Ill Patients with Suspected  Severe Pneumonia | Qiagen QIAamp Circulating Nucleic Acid Kit (col) | Illumina Nextera XT DNA Library Prep Kit (ds) |
| **RUO** | Wang | 2021 | *Frontiers in Molecular Biosciences* | Plasma Microbial Cell-Free DNA Sequencing Technology for the Diagnosis of Sepsis in the ICU | TIANamp Micro DNA Kit (col) | Homebrew (ds) |
| **RUO** | Zhang | 2015 | *Genome Biology* | Identification of low abundance microbiome in clinical samples using whole genome sequencing | Qiagen AllPrep Micro Kit (col) | Illumina TruSeq DNA-seq Library Prep Kit (ds) |
| **VAL-S** | Hong | 2018 | *Diagn Microbiol Infect Dis* | Liquid biopsy for infectious diseases: Sequencing of cell-free plasma to detect pathogen DNA in patients with invasive fungal disease | Karius DC3; modified Omega BioTek Mag-Bind cfDNA (bead) | Karius DC3; modified Tecan Ovation Ultralow System V2 (ds) |
| **VAL-S** | Blauwkamp | 2019 | *Nat Microbiol* | Analytical and clinical validation of a microbial cell-free DNA sequencing test for infectious disease | Karius DC3; modified Omega BioTek Mag-Bind cfDNA (bead) | Karius DC3; modified Tecan Ovation Ultralow System V2 (ds) |
| **VAL-S** | Farnaes | 2019 | *Diagn Microbiol Infect Dis* | Community-acquired pneumonia in children: Cell free plasma sequencing for diagnosis and management | Karius DC3; modified Omega BioTek Mag-Bind cfDNA (bead) | Karius DC3; modified Tecan Ovation Ultralow System V2 (ds) |
| **VAL-S** | Armstrong | 2019 | *Pediatr Blood Cancer* | Cell-free DNA next-generation sequencing successfully detects infectious pathogens in pediatric oncology and hematopoietic stem cell transplant patients at risk for invasive fungal disease | Karius DC3; modified Omega BioTek Mag-Bind cfDNA (bead) | Karius DC3; modified Tecan Ovation Ultralow System V2 (ds) |
| **VAL-S** | Nomura | 2019 | *BMC Infect Dis* | Rapid detection of invasive Mycobacterium chimaera disease via a novel plasma-based next-generation sequencing test | Karius DC3; modified Omega BioTek Mag-Bind cfDNA (bead) | Karius DC3; modified Tecan Ovation Ultralow System V2 (ds) |
| **VAL-S** | Rossoff | 2019 | *Open Forum Infect Dis* | Non-invasive diagnosis of infection using plasma next-generation sequencing: A single center experience | Karius DC3; modified Omega BioTek Mag-Bind cfDNA (bead) | Karius DC3; modified Tecan Ovation Ultralow System V2 (ds) |
| **VAL-S** | Camargo | 2019 | *F1000Res* | Next-generation sequencing of microbial cell-free DNA for rapid noninvasive diagnosis of infectious diseases in immunocompromised hosts | Karius DC3; modified Omega BioTek Mag-Bind cfDNA (bead) | Karius DC3; modified Tecan Ovation Ultralow System V2 (ds) |
| **VAL-S** | Goggin | 2019 | *JAMA Oncol* | Evaluation of Plasma Microbial Cell-Free DNA Sequencing to Predict Bloodstream Infection in Pediatric Patients with Relapsed or Refractory Cancer | Karius DC3; modified Omega BioTek Mag-Bind cfDNA (bead) | Karius DC3; modified Tecan Ovation Ultralow System V2 (ds) |
| **VAL-S** | Hogan | 2020 | *Clin Infect Dis* | Clinical Impact of Metagenomic Next-Generation Sequencing of Plasma Cell-Free DNA for the Diagnosis of Infectious Diseases: A Multicenter Retrospective Cohort Study | Karius DC3; modified Omega BioTek Mag-Bind cfDNA (bead) | Karius DC3; modified Tecan Ovation Ultralow System V2 (ds) |
| **VAL-S** | Witt | 2020 | *PLoS One* | Detection of microbial cell-free DNA in maternal and umbilical cord plasma in patients with chorioamnionitis using next generation sequencing | Karius DC3; modified Omega BioTek Mag-Bind cfDNA (bead) | Karius DC3; modified Tecan Ovation Ultralow System V2 (ds) |
| **VAL-S** | Lee | 2020 | *J Clin Microbiol* | Assessment of the Clinical Utility of Plasma Metagenomic Next-Generation Sequencing in a Pediatric Hospital Population | Karius DC3; modified Omega BioTek Mag-Bind cfDNA (bead) | Karius DC3; modified Tecan Ovation Ultralow System V2 (ds) |
| **VAL-S** | Branda | 2020 | *Clin Infect Dis* | Detection of Borrelia burgdorferi Cell-free DNA in Human Plasma Samples for Improved Diagnosis of Early Lyme Borreliosis | Karius DC3; modified Omega BioTek Mag-Bind cfDNA (bead) | Karius DC3; modified Tecan Ovation Ultralow System V2 (ds) |
| **VAL-S** | Niles | 2020 | *J Clin Microbiol* | Plasma Metagenomic Next Generation Sequencing Assay for Identifying Pathogens: A Retrospective Review of Test Utilization in a Large Children's Hospital | Karius DC3; modified Omega BioTek Mag-Bind cfDNA (bead) | Karius DC3; modified Tecan Ovation Ultralow System V2 (ds) |
| **VAL-S** | MacIntyre | 2020 | *Appl Health Econ Health Policy* | Budget Impact of Microbial Cell‑Free DNA Testing Using the Karius® Test as an Alternative to Invasive Procedures in Immunocompromised Patients with Suspected Invasive Fungal Infections | Karius DC3; modified Omega BioTek Mag-Bind cfDNA (bead) | Karius DC3; modified Tecan Ovation Ultralow System V2 (ds) |
| **VAL-S** | Hill | 2020 | *Clin Infect Dis* | Liquid biopsy for invasive mold infections in hematopoietic cell transplant recipients with pneumonia through next-generation sequencing of microbial cell-free DNA in plasma | Karius DC3; modified Omega BioTek Mag-Bind cfDNA (bead) | Karius DC3; modified Tecan Ovation Ultralow System V2 (ds) |
| **VAL-S** | To | 2020 | *Pediatr Infect Dis J* | Use of Plasma Metagenomic Next-generation Sequencing for Pathogen Identification in Pediatric Endocarditis | Karius DC3; modified Omega BioTek Mag-Bind cfDNA (bead) | Karius DC3; modified Tecan Ovation Ultralow System V2 (ds) |
| **VAL-S** | Kitsios | 2021 | *Respir Res* | Plasma microbial cell-free DNA load is associated with mortality in patients with COVID-19 | Karius DC3; modified Omega BioTek Mag-Bind cfDNA (bead) | Karius DC3; modified Tecan Ovation Ultralow System V2 (ds) |
| **VAL-S** | Pollock | 2021 | *Int J Tuberc Lung Dis* | Detection of Mycobacterium tuberculosis cell-free DNA to diagnose TB in pediatric and adult patients | Karius DC3; modified Omega BioTek Mag-Bind cfDNA (bead) | Karius DC3; modified Tecan Ovation Ultralow System V2 (ds) |
| **VAL-S** | Yu | 2021 | *Transplant Cell Ther* | Impact of Next-Generation Sequencing Cell-free Pathogen DNA Test on Anti-Microbial Management in Adults with Hematological Malignancies and Transplant Recipients with Suspected Infections | Karius DC3; modified Omega BioTek Mag-Bind cfDNA (bead) | Karius DC3; modified Tecan Ovation Ultralow System V2 (ds) |
| **VAL-S** | Benamu | 2021 | *Clin Infect Dis* | Plasma Microbial Cell-free DNA Next Generation Sequencing in the Diagnosis and Management of Febrile Neutropenia | Karius DC3; modified Omega BioTek Mag-Bind cfDNA (bead) | Karius DC3; modified Tecan Ovation Ultralow System V2 (ds) |
| **VAL-S** | Yang | 2021 | *Thorax* | Circulating microbial cell-free DNA is associated with inflammatory host-responses in severe pneumonia | Karius DC3; modified Omega BioTek Mag-Bind cfDNA (bead) | Karius DC3; modified Tecan Ovation Ultralow System V2 (ds) |
| **VAL-S** | Govender | 2021 | *J Clin Microbiol* | Metagenomic sequencing as a pathogen-agnostic clinical diagnostic tool for infectious diseases: a systematic review and meta-analysis of diagnostic test accuracy studies | Karius DC3; modified Omega BioTek Mag-Bind cfDNA (bead) | Karius DC3; modified Tecan Ovation Ultralow System V2 (ds) |
| **VAL-S** | Echeverria | 2021 | *J Bone Joint Surg Am* | Sequencing of circulating microbial cell-free DNA can identify pathogens in periprosthetic joint infections | Karius DC3; modified Omega BioTek Mag-Bind cfDNA (bead) | Karius DC3; modified Tecan Ovation Ultralow System V2 (ds) |
| **VAL-S** | Wilke | 2021 | *BMC Infect Dis* | Clinical application of cell-free next-generation sequencing for infectious diseases at a tertiary children's hospital | Karius DC3; modified Omega BioTek Mag-Bind cfDNA (bead) | Karius DC3; modified Tecan Ovation Ultralow System V2 (ds) |
| **VAL-S** | Tan | 2021 | *OFID* | Updated Experience of Mycobacterium Chimaera Infection: Diagnosis and Management in a Tertiary Care Center | Karius DC3; modified Omega BioTek Mag-Bind cfDNA (bead) | Karius DC3; modified Tecan Ovation Ultralow System V2 (ds) |
| **VAL-S** | Eichenberger | 2021 | *Clin Infect Dis* | Microbial Cell-Free DNA Identifies Etiology of Bacterial Bloodstream Infections, Persists Longer Than Conventional Blood Cultures, and Predicts Metastatic Infection in Staphylococcus aureus and Gram-Negative Bacteremia | Karius DC3; modified Omega BioTek Mag-Bind cfDNA (bead) | Karius DC3; modified Tecan Ovation Ultralow System V2 (ds) |
| **VAL-S** | Niles | 2022 | *Pediatr Infect Dis J* | Clinical Impact of Plasma Metagenomic Next-generation Sequencing in a Large Pediatric Cohort | Karius DC3; modified Omega BioTek Mag-Bind cfDNA (bead) | Karius DC3; modified Tecan Ovation Ultralow System V2 (ds) |
| **VAL-S** | Dworsky | 2022 | *Hosp Pediatr* | Impact of Cell-Free Next-Generation Sequencing on Management of Pediatric Complicated Pneumonia | Karius DC3; modified Omega BioTek Mag-Bind cfDNA (bead) | Karius DC3; modified Tecan Ovation Ultralow System V2 (ds) |
| **VAL-S** | Shishido | 2022 | *BMC Infectious Diseases* | Clinical impact of a metagenomic microbial plasma cell-free DNA next-generation sequencing assay on treatment decisions: a single-center retrospective study | Karius DC3; modified Omega BioTek Mag-Bind cfDNA (bead) | Karius DC3; modified Tecan Ovation Ultralow System V2 (ds) |
| **VAL-S** | Eichenberger | 2022 | *CID* | Microbial Cell-Free DNA Identifies the Causative Pathogen in Infective Endocarditis and Remains Detectable Longer Than Conventional Blood Culture in Patients with Prior Antibiotic Therapy | Karius DC3; modified Omega BioTek Mag-Bind cfDNA (bead) | Karius DC3; modified Tecan Ovation Ultralow System V2 (ds) |
| **VAL-S** | Niles | 2022 | *Diagn Microbiol Infect Dis* | Plasma Cell-Free Metagenomic Next Generation Sequencing in the Clinical Setting for the Diagnosis of Infectious Diseases: A Systematic Review and Meta-Analysis | Karius DC3; modified Omega BioTek Mag-Bind cfDNA (bead) | Karius DC3; modified Tecan Ovation Ultralow System V2 (ds) |
| **VAL-S** | Foong | 2022 | *OFID* | Clinical Impact of Non-invasive Plasma Microbial Cell-free Deoxyribonucleic Acid Sequencing for the Diagnosis and Management of Pneumocystis jirovecii Pneumonia: A Single-Center Retrospective Study | Karius DC3; modified Omega BioTek Mag-Bind cfDNA (bead) | Karius DC3; modified Tecan Ovation Ultralow System V2 (ds) |
| **VAL-S** | Vissichelli | 2023 | *Transpl Infect Dis.* | Cell-free next-generation sequencing impacts diagnosis and antimicrobial therapy in immunocompromised hosts: A retrospective study | Karius DC3; modified Omega BioTek Mag-Bind cfDNA (bead) | Karius DC3; modified Tecan Ovation Ultralow System V2 (ds) |
| **VAL-S** | Francisco | 2023 | *Antimicrobial Stewardship & Healthcare Epidemiology* | The effect of a plasma next-generation sequencing test on antimicrobial management in immunocompetent and immunocompromised patients - A single-center retrospective study | Karius DC3; modified Omega BioTek Mag-Bind cfDNA (bead) | Karius DC3; modified Tecan Ovation Ultralow System V2 (ds) |
| **VAL-S** | Hoenigl | 2023 | *JCM* | Metagenomic Next-Generation Sequencing of Plasma for Diagnosis of COVID-19-Associated Pulmonary Aspergillosis | Karius DC3; modified Omega BioTek Mag-Bind cfDNA (bead) | Karius DC3; modified Tecan Ovation Ultralow System V2 (ds) |
| **VAL-S** | Blair | 2023 | *Blood Adv* | Circulating microbial cell–free DNA is increased during neutropenia after hematopoietic stem cell transplantation | Karius DC3; modified Omega BioTek Mag-Bind cfDNA (bead) | Karius DC3; modified Tecan Ovation Ultralow System V2 (ds) |
| **VAL-S** | Heldman | 2023 | *J Infect Dis* | Serial quantitation of plasma microbial cell-free DNA before and after diagnosis of pulmonary invasive mold infections in hematopoietic cell transplant recipients | Karius DC3; modified Omega BioTek Mag-Bind cfDNA (bead) | Karius DC3; modified Tecan Ovation Ultralow System V2 (ds) |
| **VAL-S** | Park | 2023 | *ASM* | Plasma Microbial Cell-Free DNA Sequencing from over 15,000 Patients Identified a Broad Spectrum of Pathogens | Karius DC3; modified Omega BioTek Mag-Bind cfDNA (bead) | Karius DC3; modified Tecan Ovation Ultralow System V2 (ds) |
| **VAL-S** | Linder | 2023 | *OFID* | Impact of metagenomic next-generation sequencing of plasma cell-free DNA testing in the management of patients with suspected infectious diseases | Karius DC3; modified Omega BioTek Mag-Bind cfDNA (bead) | Karius DC3; modified Tecan Ovation Ultralow System V2 (ds) |
| **VAL-S** | Lisius | 2023 | *iScience* | Noninvasive diagnosis of secondary infections in COVID-19 by sequencing of plasma microbial cell-free DNA | Karius DC3; modified Omega BioTek Mag-Bind cfDNA (bead) | Karius DC3; modified Tecan Ovation Ultralow System V2 (ds) |
| **VAL-S** | Bergin | 2023 | *CID* | Plasma Microbial Cell-Free DNA Sequencing in Immunocompromised Patients with Pneumonia: A Prospective Observational Study | Karius DC3; modified Omega BioTek Mag-Bind cfDNA (bead) | Karius DC3; modified Tecan Ovation Ultralow System V2 (ds) |
| **VAL-S** | Park | 2023 | *JID* | Detection of Mpox Virus Using Microbial Cell-free DNA: the Potential of Pathogen-Agnostic Sequencing for Rapid Identification of Emerging Pathogen | Karius DC3; modified Omega BioTek Mag-Bind cfDNA (bead) | Karius DC3; modified Tecan Ovation Ultralow System V2 (ds) |
| **VAL-S** | Flurin | 2023 | *OFID* | Comparison of Blood-Based Shotgun and Targeted Metagenomic Sequencing for Microbiological Diagnosis of Infective Endocarditis | Karius DC3; modified Omega BioTek Mag-Bind cfDNA (bead) | Karius DC3; modified Tecan Ovation Ultralow System V2 (ds) |
| **VAL-S** | Duster | 2023 | *J Microbiological Methods* | Application of cell-free plama next-generation sequencing technology in the diagnosis and management of pediatric meningitis | Karius DC3; modified Omega BioTek Mag-Bind cfDNA (bead) | Karius DC3; modified Tecan Ovation Ultralow System V2 (ds) |
| **VAL-S** | Vinh  Dong | 2024 | *J Appl Lab Med.* | Elucidating the Clinical Interpretation and Impact of a Positive Plasma Cell-Free DNA Metagenomics Test Result-A Single Center Retrospective Study | Karius DC3; modified Omega BioTek Mag-Bind cfDNA (bead) | Karius DC3; modified Tecan Ovation Ultralow System V2 (ds) |
| **VAL-S** | Pizzuti | 2024 | *Infection* | Epidemiology and treatment of invasive Bartonella spp. infections in the United States | Karius DC3; modified Omega BioTek Mag-Bind cfDNA (bead) | Karius DC3; modified Tecan Ovation Ultralow System V2 (ds) |
| **VAL-S** | Wood | 2024 | *J Pediatric Infect Dis Soc* | Plasma Microbial Cell-Free DNA Sequencing for Pathogen Detection and Quantification in Children with Musculoskeletal Infections | Karius DC3; modified Omega BioTek Mag-Bind cfDNA (bead) | Karius DC3; modified Tecan Ovation Ultralow System V2 (ds) |
| **VAL-S** | Craver | 2024 | *Fetal Pediatr Pathol.* | Cell Free Microbial DNA Utilization at a Children’s Hospital | Karius DC3; modified Omega BioTek Mag-Bind cfDNA (bead) | Karius DC3; modified Tecan Ovation Ultralow System V2 (ds) |
| **VAL-S** | Williams | 2024 | *Therapeutic Advances in Infectious Disease* | Evaluation of the diagnostic utility of metagenomic next-generation sequencing testing for pathogen identification in infected hosts: a retrospective cohort study | Karius DC3; modified Omega BioTek Mag-Bind cfDNA (bead) | Karius DC3; modified Tecan Ovation Ultralow System V2 (ds) |
| **VAL-S** | Lehman | 2024 | *J Pediatric Infect Dis Soc* | Clinical Performance of Plasma Metagenomic Sequencing in Immunocompromised Pediatric Patients | Karius DC3; modified Omega BioTek Mag-Bind cfDNA (bead) | Karius DC3; modified Tecan Ovation Ultralow System V2 (ds) |
| **VAL-S** | Olthoff | 2024 | *Antimicrobial Stewardship & Healthcare Epidemiology* | Impact of metagenomic next-generation sequencing on clinical decision-making at an academic medical center, a retrospective study, Iowa, 2020–2022 | Karius DC3; modified Omega BioTek Mag-Bind cfDNA (bead) | Karius DC3; modified Tecan Ovation Ultralow System V2 (ds) |
| **VAL-S** | Daher | 2024 | *Ther Adv Infect Dis* | Clinical utility of metagenomic next-generation sequencing in fever of undetermined origin | Karius DC3; modified Omega BioTek Mag-Bind cfDNA (bead) | Karius DC3; modified Tecan Ovation Ultralow System V2 (ds) |
| **VAL-S** | Castejon-Ramirez | 2024 | *Journal of the Pediatric Infectious Diseases Society* | Plasma Metagenomic Sequencing in Immunocompromised Children: a Call for Caution in the Interpretation of Results | Karius DC3; modified Omega BioTek Mag-Bind cfDNA (bead) | Karius DC3; modified Tecan Ovation Ultralow System V2 (ds) |
| **VAL-S** | Aftandilian | 2024 | *Eur J Haematol* | Plasma microbial cell-free DNA following chimeric antigen receptor T cell therapy in pediatric patients with relapsed/refractory leukemia | Karius DC3; modified Omega BioTek Mag-Bind cfDNA (bead) | Karius DC3; modified Tecan Ovation Ultralow System V2 (ds) |
| **VAL-S** | Thompson | 2024 | *Diagnostic Microbiology & Infectious Disease* | Utility of Microbial Cell Free DNA Next-Generation Sequencing for Diagnosis and Management of Infectious Diseases | Karius DC3; modified Omega BioTek Mag-Bind cfDNA (bead) | Karius DC3; modified Tecan Ovation Ultralow System V2 (ds) |
| **VAL-S** | Huygens | 2024 | *OFID* | Diagnostic value of microbial cell-free DNA sequencing for suspected invasive fungal infections: a retrospective multicenter cohort study | Karius DC3; modified Omega BioTek Mag-Bind cfDNA (bead) | Karius DC3; modified Tecan Ovation Ultralow System V2 (ds) |
| **VAL-S** | Olthoff | 2024 | *Antimicrobial Stewardship & Healthcare Epidemiology* | Impact of metagenomic next-generation sequencing on clinical decision-making at an academic medical center, a retrospective study, Iowa, 2020–2022 | Karius DC3; modified Omega BioTek Mag-Bind cfDNA (bead) | Karius DC3; modified Tecan Ovation Ultralow System V2 (ds) |
| **VAL-S** | Daher | 2024 | *Ther Adv Infect Dis* | Clinical utility of metagenomic next-generation sequencing in fever of undetermined origin | Karius DC3; modified Omega BioTek Mag-Bind cfDNA (bead) | Karius DC3; modified Tecan Ovation Ultralow System V2 (ds) |
| **VAL-S** | Castejon-Ramirez | 2024 | *Journal of the Pediatric Infectious Diseases Society* | Plasma Metagenomic Sequencing in Immunocompromised Children: a Call for Caution in the Interpretation of Results | Karius DC3; modified Omega BioTek Mag-Bind cfDNA (bead) | Karius DC3; modified Tecan Ovation Ultralow System V2 (ds) |
| **VAL-S** | Aftandilian | 2024 | *Eur J Haematol* | Plasma microbial cell-free DNA following chimeric antigen receptor T cell therapy in pediatric patients with relapsed/refractory leukemia | Karius DC3; modified Omega BioTek Mag-Bind cfDNA (bead) | Karius DC3; modified Tecan Ovation Ultralow System V2 (ds) |
| **VAL-S** | Thompson | 2024 | *Diagnostic Microbiology & Infectious Disease* | Utility of Microbial Cell Free DNA Next-Generation Sequencing for Diagnosis and Management of Infectious Diseases | Karius DC3; modified Omega BioTek Mag-Bind cfDNA (bead) | Karius DC3; modified Tecan Ovation Ultralow System V2 (ds) |
| **VAL-S** | Huygens | 2024 | *OFID* | Diagnostic value of microbial cell-free DNA sequencing for suspected invasive fungal infections: a retrospective multicenter cohort study | Karius DC3; modified Omega BioTek Mag-Bind cfDNA (bead) | Karius DC3; modified Tecan Ovation Ultralow System V2 (ds) |
| **VAL-S** | El Zein | 2024 | *OFID* | Acute Coxiella burnetii Infection: A 10-Year Clinical Experience at a Tertiary Care Center in the United States | Karius DC3; modified Omega BioTek Mag-Bind cfDNA (bead) | Karius DC3; modified Tecan Ovation Ultralow System V2 (ds) |
| **VAL-S** | Petri | 2024 | *OFID* | Plasma Microbial Cell-free DNA Next-generation Sequencing can be a Useful Diagnostic Tool in Patients with Osteoarticular Infections | Karius DC3; modified Omega BioTek Mag-Bind cfDNA (bead) | Karius DC3; modified Tecan Ovation Ultralow System V2 (ds) |
| **VAL-S** | Thompson | 2024 | *PharmacoEconomics* | Cost-Effectiveness of Plasma Microbial Cell-Free DNA Sequencing When Added to Usual Care Diagnostic Testing for Immunocompromised Host Pneumonia | Karius DC3; modified Omega BioTek Mag-Bind cfDNA (bead) | Karius DC3; modified Tecan Ovation Ultralow System V2 (ds) |
| **VAL-S** | Berger | 2024 | *OFID* | Utility of serial microbial cell-free DNA sequencing for inpatient and outpatient pathogen surveillance among allogeneic hematopoietic stem cell transplant recipients | Karius DC3; modified Omega BioTek Mag-Bind cfDNA (bead) | Karius DC3; modified Tecan Ovation Ultralow System V2 (ds) |
| **VAL-S** | Vinnakota | 2024 | *Mayo Clinic Proceedings: Innovations, Quality & Outcomes* | Multimodal Imaging in Mycobacterium Chimaera Cardiovascular Infections: The Mayo Clinic Experience | Karius DC3; modified Omega BioTek Mag-Bind cfDNA (bead) | Karius DC3; modified Tecan Ovation Ultralow System V2 (ds) |
| **VAL-S** | Madut | 2024 | *OFID* | Clinical utility of plasma microbial cell-free DNA sequencing among immunocompromised patients with pneumonia | Karius DC3; modified Omega BioTek Mag-Bind cfDNA (bead) | Karius DC3; modified Tecan Ovation Ultralow System V2 (ds) |
| **VAL-S** | Rodriguez | 2024 | *Diagn Microbiol Infect Dis* | Pathogen Kinetics and Detection by Next-Generation Sequencing in Pediatric Complicated Pneumonia | Karius DC3; modified Omega BioTek Mag-Bind cfDNA (bead) | Karius DC3; modified Tecan Ovation Ultralow System V2 (ds) |
| **VAL-S** | Shean | 2024 | *JCM* | A retrospective observational study of mNGS test utilization to examine the role of diagnostic stewardship at two academic medical centers | Karius DC3; modified Omega BioTek Mag-Bind cfDNA (bead) | Karius DC3; modified Tecan Ovation Ultralow System V2 (ds) |
| **VAL-S** | Christians | 2024 | *JCM* | Analytical and clinical validation of direct detection of antimicrobial resistance markers by plasma microbial cell-free DNA sequencing | Karius DC3; modified Omega BioTek Mag-Bind cfDNA (bead) | Karius DC3; modified Tecan Ovation Ultralow System V2 (ds) |
| **VAL-S** | Jenkins | 2024 | *medRxiv* | Use of Metagenomic Microbial Plasma Cell-Free DNA Next-Generation Sequencing Assay in Outpatient Rheumatology Practice | Karius DC3; modified Omega BioTek Mag-Bind cfDNA (bead) | Karius DC3; modified Tecan Ovation Ultralow System V2 (ds) |
| **VAL-S** | Wasserman | 2024 | *Pediatric Pulmonology* | Plasma Microbial Cell‐Free DNA Metagenomic Next‐Generation Sequencing in People With Cystic Fibrosis | Karius DC3; modified Omega BioTek Mag-Bind cfDNA (bead) | Karius DC3; modified Tecan Ovation Ultralow System V2 (ds) |
| **VAL-S** | Drake | 2024 | *Antimicrob Steward Healthc Epidemiol.* | A one-year hospital system review of plasma next-generation sequencing in a mixed population | Karius DC3; modified Omega BioTek Mag-Bind cfDNA (bead) | Karius DC3; modified Tecan Ovation Ultralow System V2 (ds) |
| **VAL-S** | Shah | 2024 | *Ther Adv Infect Dis.* | Clinical utility of plasma microbial cell-free DNA sequencing in determining microbiologic etiology of infectious syndromes in solid organ transplant recipients | Karius DC3; modified Omega BioTek Mag-Bind cfDNA (bead) | Karius DC3; modified Tecan Ovation Ultralow System V2 (ds) |
| **VAL-S** | Handel | 2024 | *PIDJ* | Plasma Microbial Cell-Free DNA Sequencing for Diagnosis of Pediatric Lyme Disease | Karius DC3; modified Omega BioTek Mag-Bind cfDNA (bead) | Karius DC3; modified Tecan Ovation Ultralow System V2 (ds) |
| **VAL-S** | Graf | 2025 | *J Appl Lab Med.* | One Size Fits Small: The Narrow Utility for Plasma Metagenomics | Karius DC3; modified Omega BioTek Mag-Bind cfDNA (bead) | Karius DC3; modified Tecan Ovation Ultralow System V2 (ds) |
| **VAL-S** | Allen | 2025 | *J Appl Lab Med.* | A Committee-Based Diagnostic Stewardship Model for Pathogen Metagenomic Sequencing in Children | Karius DC3; modified Omega BioTek Mag-Bind cfDNA (bead) | Karius DC3; modified Tecan Ovation Ultralow System V2 (ds) |
| **VAL-S** | Xu | 2025 | *Pediatr Investig* | Efficacy of microbial cell-free DNA testing for detecting pathogens in pediatric patients with head and neck infections—An initial study | Karius DC3; modified Omega BioTek Mag-Bind cfDNA (bead) | Karius DC3; modified Tecan Ovation Ultralow System V2 (ds) |
| **VAL-S** | Andeen | 2025 | *Kidney International Reports* | Bartonella endocarditis-associated glomerulonephritis: a mimicker of autoimmunity and vasculitis | Karius DC3; modified Omega BioTek Mag-Bind cfDNA (bead) | Karius DC3; modified Tecan Ovation Ultralow System V2 (ds) |
| **VAL-S** | Gali | 2025 | *JCM* | Evaluating the clinical utility of Aspergillus, Mucorales, and Nocardia bronchoalveolar PCRs for the diagnosis of invasive pulmonary infections in patients with hematological malignancies | Karius DC3; modified Omega BioTek Mag-Bind cfDNA (bead) | Karius DC3; modified Tecan Ovation Ultralow System V2 (ds) |
| **VAL-S** | Berger | 2025 | *Ther Adv Infect Dis.* | The utilization of microbial cell-free DNA next-generation sequencing for the detection of human herpesvirus-8 in a quaternary care center | Karius DC3; modified Omega BioTek Mag-Bind cfDNA (bead) | Karius DC3; modified Tecan Ovation Ultralow System V2 (ds) |
| **VAL-S** | Kaur | 2025 | *Infect Control Hosp Epidemiol* | Real-world clinical impact of plasma cell-free DNA metagenomic next-generation sequencing assay | Karius DC3; modified Omega BioTek Mag-Bind cfDNA (bead) | Karius DC3; modified Tecan Ovation Ultralow System V2 (ds) |
| **VAL-S** | Ranganath | 2025 | *OFID* | From Chart Biopsy to Liquid Biopsy: Evaluating the Diagnostic Yield and Clinical Impact of Plasma Microbial Cell-Free DNA Next-Generation Sequencing in the Management of Fever of Unknown Origin | Karius DC3; modified Omega BioTek Mag-Bind cfDNA (bead) | Karius DC3; modified Tecan Ovation Ultralow System V2 (ds) |
| **VAL-S** | Park | 2025 | *Pathogens and Immunity* | Plasma mcfDNA Sequencing May Improve Usual Care Diagnostics to Detect HHV-8 Among Outpatient People with Advanced HIV | Karius DC3; modified Omega BioTek Mag-Bind cfDNA (bead) | Karius DC3; modified Tecan Ovation Ultralow System V2 (ds) |
| **VAL-S** | Kim | 2025 | *OFID* | Illuminating the Challenges and Diagnostic Utility of Plasma Microbial Cell-Free DNA Sequencing in Suspected Infective Endocarditis: A Retrospective Observational Cohort Study | Karius DC3; modified Omega BioTek Mag-Bind cfDNA (bead) | Karius DC3; modified Tecan Ovation Ultralow System V2 (ds) |
| **VAL-S** | Ahmed | 2025 | *PIDJ* | Plasma Microbial Cell-free DNA Sequencing for the Detection of Kingella kingae Pediatric Spinal Infections | Karius DC3; modified Omega BioTek Mag-Bind cfDNA (bead) | Karius DC3; modified Tecan Ovation Ultralow System V2 (ds) |
| **VAL-S** | Karchmer | 2025 | *CID* | Quantitative Microbial Cell-Free DNA Sequencing from Plasma: A Potential Biomarker for the Diagnosis of Staphylococcal Infection of Cardiac Implantable Electronic Devices | Karius DC3; modified Omega BioTek Mag-Bind cfDNA (bead) | Karius DC3; modified Tecan Ovation Ultralow System V2 (ds) |
| **VAL-S** | Sim | 2025 | *CID* | Plasma microbial cell-free DNA Metagenomic Sequencing for Diagnosis of Invasive Fungal Diseases Among High Risk Outpatient and Inpatient Immunocompromised Hosts | Karius DC3; modified Omega BioTek Mag-Bind cfDNA (bead) | Karius DC3; modified Tecan Ovation Ultralow System V2 (ds) |
| **VAL-S** | Jenkins | 2025 | *Journal of Rheumatology* | Use of Metagenomic Microbial Plasma Cell-Free DNA Next-Generation Sequencing Assay in Outpatient Rheumatology Practice | Karius DC3; modified Omega BioTek Mag-Bind cfDNA (bead) | Karius DC3; modified Tecan Ovation Ultralow System V2 (ds) |
| **VAL-S** | Goren | 2025 | *Access Microbiology* | Plasma Metagenomic Cell-Free DNA Sequencing Identifies Clinically Unsuspected Cases of Toxoplasma gondii Infection in Immunocompromised Patients | Karius DC3; modified Omega BioTek Mag-Bind cfDNA (bead) | Karius DC3; modified Tecan Ovation Ultralow System V2 (ds) |
| **VAL-S** | Centeno | 2025 | *Antimicrob Steward Healthc Epidemiol* | Utility of microbial cell-free DNA sequencing in the diagnosis of mycobacterial infections in a quaternary care center | Karius DC3; modified Omega BioTek Mag-Bind cfDNA (bead) | Karius DC3; modified Tecan Ovation Ultralow System V2 (ds) |
| **VAL-S** | Czech | 2025 | *OFID* | Usefulness and limitations of polymerase chain reaction (PCR) for the diagnosis and management of toxoplasmosis following allogeneic hematopoietic cell transplant: Single center experience with 31 patients over 16 years | Karius DC3; modified Omega BioTek Mag-Bind cfDNA (bead) | Karius DC3; modified Tecan Ovation Ultralow System V2 (ds) |
| **VAL-S** | Jung | 2025 | *Pathogens* | A Cell-Free DNA Plasma Next-Generation Sequencing Test-Is It Worth the Cost? | Karius DC3; modified Omega BioTek Mag-Bind cfDNA (bead) | Karius DC3; modified Tecan Ovation Ultralow System V2 (ds) |
| **VAL-R** | Parrish | 2022 | *Transpl Infect Dis.* | Metagenomics in infectious disease diagnostics: Toward best-use practices to optimize actionable results | Karius DC3; modified Omega BioTek Mag-Bind cfDNA (bead) | Karius DC3; modified Tecan Ovation Ultralow System V2 (ds) |
| **VAL-R** | Han | 2020 | *Theranostics* | Liquid biopsy for infectious diseases: a focus on microbial cell-free DNA sequencing. | Karius DC3; modified Omega BioTek Mag-Bind cfDNA (bead) | Karius DC3; modified Tecan Ovation Ultralow System V2 (ds) |
| **VAL-R** | Freeman  Weiss | 2021 | *J Fungi* | The Evolving Landscape of Fungal Diagnostics, Current and Emerging Microbiological Approaches | Karius DC3; modified Omega BioTek Mag-Bind cfDNA (bead) | Karius DC3; modified Tecan Ovation Ultralow System V2 (ds) |
| **VAL-R** | Morales | 2021 | *Clinical Microbiology Newsletter* | The Next Big Thing? Next-Generation Sequencing of Microbial Cell-Free DNA Using the Karius Test | Karius DC3; modified Omega BioTek Mag-Bind cfDNA (bead) | Karius DC3; modified Tecan Ovation Ultralow System V2 (ds) |
| **VAL-R** | Casto | 2021 | *Blood Reviews* | Diagnosis of infectious diseases in immunocompromised hosts using metagenomic next generation sequencing-based diagnostics | Karius DC3; modified Omega BioTek Mag-Bind cfDNA (bead) | Karius DC3; modified Tecan Ovation Ultralow System V2 (ds) |
| **VAL-R** | Burillo | 2022 | *Expert Review of Molecular Diagnostics* | Faster infection diagnostics for intensive care unit (ICU) patients | Karius DC3; modified Omega BioTek Mag-Bind cfDNA (bead) | Karius DC3; modified Tecan Ovation Ultralow System V2 (ds) |
| **VAL-R** | Tjandra | 2022 | *Antibiotics (Basel)* | Diagnosis of Bloodstream Infections: An Evolution of Technologies towards Accurate and Rapid Identification and Antibiotic Susceptibility Testing | Karius DC3; modified Omega BioTek Mag-Bind cfDNA (bead) | Karius DC3; modified Tecan Ovation Ultralow System V2 (ds) |
| **VAL-R** | Wieczorkiewicz | 2022 | *IDSE Review* | Implementing Rapid Diagnostic Tests to Augment Antimicrobial Stewardship Programs: The Time Is Now | Karius DC3; modified Omega BioTek Mag-Bind cfDNA (bead) | Karius DC3; modified Tecan Ovation Ultralow System V2 (ds) |
| **VAL-R** | Huang | 2022 | *Curr Heart Fail Rep* | Biomarker-Based Assessment for Infectious Risk Before and After Heart Transplantation | Karius DC3; modified Omega BioTek Mag-Bind cfDNA (bead) | Karius DC3; modified Tecan Ovation Ultralow System V2 (ds) |
| **VAL-R** | Hankins | 2022 | *Clinical Microbiology Newsletter* | A Little Goes a Long Way: Pediatric Bloodstream Infections and Blood Culture Practices | Karius DC3; modified Omega BioTek Mag-Bind cfDNA (bead) | Karius DC3; modified Tecan Ovation Ultralow System V2 (ds) |
| **VAL-R** | Hilt | 2022 | *Genes* | Next Generation and Other Sequencing Technologies in Diagnostic Microbiology and Infectious Diseases | Karius DC3; modified Omega BioTek Mag-Bind cfDNA (bead) | Karius DC3; modified Tecan Ovation Ultralow System V2 (ds) |
| **VAL-R** | Bell | 2023 | *COID* | Deciphering the potential of plasma cell-free metagenomic next-generation sequencing using the Karius test | Karius DC3; modified Omega BioTek Mag-Bind cfDNA (bead) | Karius DC3; modified Tecan Ovation Ultralow System V2 (ds) |
| **VAL-R** | Hill | 2023 | *OFID* | A Systematic Literature Review to Identify Diagnostic Gaps in Managing Immunocompromised Patients with Cancer and Suspected Infection | Karius DC3; modified Omega BioTek Mag-Bind cfDNA (bead) | Karius DC3; modified Tecan Ovation Ultralow System V2 (ds) |
| **VAL-R** | Park | 2023 | *Pediatr Blood Cancer* | Gaps in diagnosing suspected infection in immunocompromised children with cancer: A systematic review | Karius DC3; modified Omega BioTek Mag-Bind cfDNA (bead) | Karius DC3; modified Tecan Ovation Ultralow System V2 (ds) |
| **VAL-R** | Park | 2024 | *Clin Microbiol Infect* | Diagnostic Limitations and Challenges in Current Clinical Guidelines and Potential Application of Metagenomic Sequencing to Manage Pulmonary Invasive Fungal Infections in Patients with Haematological Malignancies | Karius DC3; modified Omega BioTek Mag-Bind cfDNA (bead) | Karius DC3; modified Tecan Ovation Ultralow System V2 (ds) |
| **VAL-R** | Marra | 2024 | *Diagn Microbiol Infect Dis* | Metagenomic next-generation sequencing in patients with fever of unknown origin: A comprehensive systematic literature review and meta-analysis | Karius DC3; modified Omega BioTek Mag-Bind cfDNA (bead) | Karius DC3; modified Tecan Ovation Ultralow System V2 (ds) |
| **VAL-R** | Park | 2025 | *OFID* | A Systematic Literature Review to Determine Gaps in Diagnosing Suspected Infection in Solid Organ Transplant Recipients | Karius DC3; modified Omega BioTek Mag-Bind cfDNA (bead) | Karius DC3; modified Tecan Ovation Ultralow System V2 (ds) |
| **VAL-R** | Khalil | 2025 | *CID* | The Next Step: Role of Metagenomic Next Generation Sequencing for Microbial Detection in Culture-negative Cardiovascular infections | Karius DC3; modified Omega BioTek Mag-Bind cfDNA (bead) | Karius DC3; modified Tecan Ovation Ultralow System V2 (ds) |
| **VAL-R** | Camargo | 2025 | *Curr Opin Infect Dis* | Microbial cell-free DNA for diagnosis of bacterial and fungal infection in the immunocompromised host - what do we know? | Karius DC3; modified Omega BioTek Mag-Bind cfDNA (bead) | Karius DC3; modified Tecan Ovation Ultralow System V2 (ds) |
| **VAL-R** | Chen | 2025 | *JCM* | Transforming tuberculosis diagnosis with clinical metagenomics: progress and roadblocks | Karius DC3; modified Omega BioTek Mag-Bind cfDNA (bead) | Karius DC3; modified Tecan Ovation Ultralow System V2 (ds) |
| **VAL-C** | Abril | 2016 | *Open Forum Infect Dis* | Diagnosis of Capnocytophaga canimorsus Sepsis by Whole-Genome Next-Generation Sequencing | Karius DC3; modified Omega BioTek Mag-Bind cfDNA (bead) | Karius DC3; modified Tecan Ovation Ultralow System V2 (ds) |
| **VAL-C** | Fung | 2018 | *Open Forum Infect Dis* | Plasma Cell-free DNA Next-generation Sequencing to Diagnose and Monitor Infections in Allogeneic Hematopoietic Stem Cell Transplant Patients | Karius DC3; modified Omega BioTek Mag-Bind cfDNA (bead) | Karius DC3; modified Tecan Ovation Ultralow System V2 (ds) |
| **VAL-C** | Zhou | 2019 | *Open AIDS* | Utility of Whole-Genome Next-Generation Sequencing of Plasma in Identifying Opportunistic Infections in HIV/AIDS | Karius DC3; modified Omega BioTek Mag-Bind cfDNA (bead) | Karius DC3; modified Tecan Ovation Ultralow System V2 (ds) |
| **VAL-C** | Vudatha | 2019 | *J Vasc Surg Cases Innov Tech* | Rapid detection of bacille Calmette-Guérin-associated mycotic aortic aneurysm using novel cell-free DNA assay | Karius DC3; modified Omega BioTek Mag-Bind cfDNA (bead) | Karius DC3; modified Tecan Ovation Ultralow System V2 (ds) |
| **VAL-C** | Kondo | 2019 | *Open Forum Infect Dis* | Diagnosis and Genotyping of Coxiella burnetii Endocarditis in a Patient with Prosthetic Pulmonary Valve Replacement Using Next-Generation Sequencing of Plasma Microbial Cell-Free DNA | Karius DC3; modified Omega BioTek Mag-Bind cfDNA (bead) | Karius DC3; modified Tecan Ovation Ultralow System V2 (ds) |
| **VAL-C** | Downey | 2020 | *J Pediatric Infect Dis Soc* | Identification of an Emergent Pathogen, Bartonella vinsonii, Using Next-Generation Sequencing in a Patient With Culture-Negative Endocarditis | Karius DC3; modified Omega BioTek Mag-Bind cfDNA (bead) | Karius DC3; modified Tecan Ovation Ultralow System V2 (ds) |
| **VAL-C** | Steinbrink | 2020 | *Med Mycol Case Rep* | The robust and rapid role of molecular testing in precision fungal diagnostics: A case report | Karius DC3; modified Omega BioTek Mag-Bind cfDNA (bead) | Karius DC3; modified Tecan Ovation Ultralow System V2 (ds) |
| **VAL-C** | Ramchandar | 2020 | *Pediatr Blood Cancer* | Identification of disseminated toxoplasmosis by plasma next-generation sequencing in a teenager with rapidly progressive multiorgan failure following haploidentical stem cell transplantation | Karius DC3; modified Omega BioTek Mag-Bind cfDNA (bead) | Karius DC3; modified Tecan Ovation Ultralow System V2 (ds) |
| **VAL-C** | Stafford | 2020 | *Case Rep Obstet Gynecol* | Successful Detection of Unrecognized Rickettsia typhi in Pregnancy Using Cell-Free Next-Generation Sequencing | Karius DC3; modified Omega BioTek Mag-Bind cfDNA (bead) | Karius DC3; modified Tecan Ovation Ultralow System V2 (ds) |
| **VAL-C** | Kalyatanda | 2020 | *Open Forum Infect Dis* | Rapid, non-invasive diagnosis of Balamuthia mandrillaris encephalitis by a plasma-based next generation sequencing test | Karius DC3; modified Omega BioTek Mag-Bind cfDNA (bead) | Karius DC3; modified Tecan Ovation Ultralow System V2 (ds) |
| **VAL-C** | Zafar | 2020 | *Cureus* | Multiloculated Liver Abscess Caused by Fusobacterium: Role of Karius Testing in Diagnosis | Karius DC3; modified Omega BioTek Mag-Bind cfDNA (bead) | Karius DC3; modified Tecan Ovation Ultralow System V2 (ds) |
| **VAL-C** | Asif | 2020 | *BMJ Case Rep* | Rare case of Prevotella pleuritidis lung abscess | Karius DC3; modified Omega BioTek Mag-Bind cfDNA (bead) | Karius DC3; modified Tecan Ovation Ultralow System V2 (ds) |
| **VAL-C** | Asif | 2020 | *BMJ Case Rep* | Rare case of Ureaplasma parvum septic arthritis in an immunocompetent patient | Karius DC3; modified Omega BioTek Mag-Bind cfDNA (bead) | Karius DC3; modified Tecan Ovation Ultralow System V2 (ds) |
| **VAL-C** | Tang  Girdwood | 2020 | *Hosp Pediatr* | Cell-Free DNA Sequencing, Pathogen Detection, and the Journey to Value | Karius DC3; modified Omega BioTek Mag-Bind cfDNA (bead) | Karius DC3; modified Tecan Ovation Ultralow System V2 (ds) |
| **VAL-C** | Zou | 2020 | *Journal of Infectious Diseases and Epidemiology* | Non-Invasive Diagnosis of Pulmonary Kaposi Sarcoma: A Case Report | Karius DC3; modified Omega BioTek Mag-Bind cfDNA (bead) | Karius DC3; modified Tecan Ovation Ultralow System V2 (ds) |
| **VAL-C** | Roy | 2020 | *BMJ Case Rep* | Rare Presentation of Toxoplasma Pneumonitis in the Absence of Neurological Symptoms in an AIDS Patient and Use of Next-Generation Sequencing for Diagnosis | Karius DC3; modified Omega BioTek Mag-Bind cfDNA (bead) | Karius DC3; modified Tecan Ovation Ultralow System V2 (ds) |
| **VAL-C** | Bansal | 2020 | *Am J Med* | Blastomycosis Detected by Microbial Cell-Free DNA in Renal Transplant Recipient | Karius DC3; modified Omega BioTek Mag-Bind cfDNA (bead) | Karius DC3; modified Tecan Ovation Ultralow System V2 (ds) |
| **VAL-C** | Roy | 2020 | *J Int Assoc Provid AIDS Care* | Rapid Diagnosis of Invasive Aspergillosis and Active Hepatitis-B Virus Co-Infection in a HIV-1 Infected Patient Using Cell Free DNA Sequencing | Karius DC3; modified Omega BioTek Mag-Bind cfDNA (bead) | Karius DC3; modified Tecan Ovation Ultralow System V2 (ds) |
| **VAL-C** | Lieberman | 2021 | *Front Pediatr* | Case Report: Comparison of Plasma Metagenomics to Bacterial PCR in a Case of Prosthetic Valve Endocarditis | Karius DC3; modified Omega BioTek Mag-Bind cfDNA (bead) | Karius DC3; modified Tecan Ovation Ultralow System V2 (ds) |
| **VAL-C** | Fettinger | 2021 | *Pediatric Infectious Disease Journal* | An 11-year-old Male With X-linked Agammaglobulinemia and Persistent Abdominal Pain | Karius DC3; modified Omega BioTek Mag-Bind cfDNA (bead) | Karius DC3; modified Tecan Ovation Ultralow System V2 (ds) |
| **VAL-C** | Pareek | 2021 | *J Neuroophthalmol* | Double Vision and Gait Ataxia in a Immunocompetent 9Year-Old Girl With Intracranial Phaeohyphomycosis | Karius DC3; modified Omega BioTek Mag-Bind cfDNA (bead) | Karius DC3; modified Tecan Ovation Ultralow System V2 (ds) |
| **VAL-C** | Garnica | 2021 | *Braz J Infect Dis* | Metagenomic next-generation sequencing (mNGS) for diagnostically challenging infectious diseases in patients with acute leukemia | Karius DC3; modified Omega BioTek Mag-Bind cfDNA (bead) | Karius DC3; modified Tecan Ovation Ultralow System V2 (ds) |
| **VAL-C** | Shishido | 2021 | *Transpl Infect Dis* | Diagnosis of central nervous system invasive aspergillosis in a liver transplant recipient using microbial cell-free next generation DNA sequencing | Karius DC3; modified Omega BioTek Mag-Bind cfDNA (bead) | Karius DC3; modified Tecan Ovation Ultralow System V2 (ds) |
| **VAL-C** | Centeno | 2021 | *Open Forum Infect Dis* | Characteristics of Rickettsia typhi Infections Detected with Next-Generation Sequencing of Microbial Cell-Free DNA in a Tertiary Care Hospital | Karius DC3; modified Omega BioTek Mag-Bind cfDNA (bead) | Karius DC3; modified Tecan Ovation Ultralow System V2 (ds) |
| **VAL-C** | Huang | 2021 | *Open Forum Infect Dis* | Infected Aneurysm of the Native Aorta due to Coccidioides posadasii | Karius DC3; modified Omega BioTek Mag-Bind cfDNA (bead) | Karius DC3; modified Tecan Ovation Ultralow System V2 (ds) |
| **VAL-C** | Dukkipati | 2021 | *Case Reports in Nephrology* | Bartonella-Associated Endocarditis with Severe Active Crescentic Glomerulonephritis and Acute Renal Failure | Karius DC3; modified Omega BioTek Mag-Bind cfDNA (bead) | Karius DC3; modified Tecan Ovation Ultralow System V2 (ds) |
| **VAL-C** | Zielke | 2021 | *J Neurosurg Case Lessons* | Hypertrophic cranial pachymeningitis and orbital apex syndrome secondary to infection of the eye: illustrative case | Karius DC3; modified Omega BioTek Mag-Bind cfDNA (bead) | Karius DC3; modified Tecan Ovation Ultralow System V2 (ds) |
| **VAL-C** | Martin-Blais | 2021 | *Transplant Infectious Disease* | Intestinal mucormycosis initially identified by next-generation sequencing of cell-free DNA | Karius DC3; modified Omega BioTek Mag-Bind cfDNA (bead) | Karius DC3; modified Tecan Ovation Ultralow System V2 (ds) |
| **VAL-C** | Hudson | 2021 | *Emerg Infect Dis* | Acute Chagas Disease in Central Texas Manifesting as Orbital Cellulitis | Karius DC3; modified Omega BioTek Mag-Bind cfDNA (bead) | Karius DC3; modified Tecan Ovation Ultralow System V2 (ds) |
| **VAL-C** | Obeid | 2021 | *Transpl Infect Dis* | Hemophagocytic lymphohistiocytosis induced by Toxoplasma gondii infection diagnosed by a bone marrow biopsy and DNA next-generation sequencing in an allogeneic hematopoietic stem cell transplant recipient | Karius DC3; modified Omega BioTek Mag-Bind cfDNA (bead) | Karius DC3; modified Tecan Ovation Ultralow System V2 (ds) |
| **VAL-C** | Radcliffe | 2021 | *Pathogens* | Pott Disease: A Tale of Two Cases | Karius DC3; modified Omega BioTek Mag-Bind cfDNA (bead) | Karius DC3; modified Tecan Ovation Ultralow System V2 (ds) |
| **VAL-C** | Brewer | 2021 | *BMC Infectious Diseases* | Detection and treatment of cerebral toxoplasmosis in an aplastic pediatric post‑allogeneic hematopoietic cell transplant patient: a case report | Karius DC3; modified Omega BioTek Mag-Bind cfDNA (bead) | Karius DC3; modified Tecan Ovation Ultralow System V2 (ds) |
| **VAL-C** | Munjal | 2021 | *Pediatrics* | Next-Generation Sequencing as an Auxiliary Tool in Pediatric Laryngeal Lymphoma Diagnosis | Karius DC3; modified Omega BioTek Mag-Bind cfDNA (bead) | Karius DC3; modified Tecan Ovation Ultralow System V2 (ds) |
| **VAL-C** | Narayanasamy | 2021 | *OFID* | Curvularia alcornii Aortic Pseudoaneurysm Following Aortic Valve Replacement: Case Report and Review of the Literature | Karius DC3; modified Omega BioTek Mag-Bind cfDNA (bead) | Karius DC3; modified Tecan Ovation Ultralow System V2 (ds) |
| **VAL-C** | Patel | 2021 | *IDCases* | A case of Bartonella henselae native valve endocarditis presenting with crescentic glomerulonephritis | Karius DC3; modified Omega BioTek Mag-Bind cfDNA (bead) | Karius DC3; modified Tecan Ovation Ultralow System V2 (ds) |
| **VAL-C** | Aquino | 2021 | *Catherization & Cardiovascular Interventions* | Gore Cardioform ASD device thrombus weeks after COVID-19 infection | Karius DC3; modified Omega BioTek Mag-Bind cfDNA (bead) | Karius DC3; modified Tecan Ovation Ultralow System V2 (ds) |
| **VAL-C** | Karim | 2022 | *Cureus* | Klebsiella Discitis in a 15-Year-Old Male Diagnosed With Plasma Microbial Cell-Free DNA Next-Generation Sequencing Test: A Case Report | Karius DC3; modified Omega BioTek Mag-Bind cfDNA (bead) | Karius DC3; modified Tecan Ovation Ultralow System V2 (ds) |
| **VAL-C** | Sabha | 2022 | *Clin Infect Dis* | A Case of Persistent Aortic Graft and Retroperitoneal Enhancement on FDG PET/CT | Karius DC3; modified Omega BioTek Mag-Bind cfDNA (bead) | Karius DC3; modified Tecan Ovation Ultralow System V2 (ds) |
| **VAL-C** | Parzen-Johnson | 2022 | *IDCases* | Plasma next-generation sequencing for diagnosis of amebic liver abscess in a non-endemic area | Karius DC3; modified Omega BioTek Mag-Bind cfDNA (bead) | Karius DC3; modified Tecan Ovation Ultralow System V2 (ds) |
| **VAL-C** | Arrighi-Allisan | 2022 | *J Neurosurgery* | Utility of liquid biopsy in diagnosing isolated cerebral phaeohyphomycosis: illustrative case | Karius DC3; modified Omega BioTek Mag-Bind cfDNA (bead) | Karius DC3; modified Tecan Ovation Ultralow System V2 (ds) |
| **VAL-C** | Shephard | 2022 | *Medical Mycology Case Reports* | Early diagnosis with sequencing and successful treatment of Bipolaris prosthetic valve endocarditis | Karius DC3; modified Omega BioTek Mag-Bind cfDNA (bead) | Karius DC3; modified Tecan Ovation Ultralow System V2 (ds) |
| **VAL-C** | Solanky | 2022 | *Frontiers Tropical Diseases* | Utility of Plasma Microbial Cell-Free DNA Decay Kinetics after Aortic Valve Replacement for Bartonella Endocarditis: Case Report | Karius DC3; modified Omega BioTek Mag-Bind cfDNA (bead) | Karius DC3; modified Tecan Ovation Ultralow System V2 (ds) |
| **VAL-C** | Vasishta | 2022 | *Transpl Infect Dis* | Pulmonary nocardiosis in a heart transplant recipient diagnosed with plasma next-generation sequencing | Karius DC3; modified Omega BioTek Mag-Bind cfDNA (bead) | Karius DC3; modified Tecan Ovation Ultralow System V2 (ds) |
| **VAL-C** | Paras | 2022 | *NEJM* | Case 13-2022: A 56-Year-Old Man with Myalgias, Fever, and Bradycardia | Karius DC3; modified Omega BioTek Mag-Bind cfDNA (bead) | Karius DC3; modified Tecan Ovation Ultralow System V2 (ds) |
| **VAL-C** | Ardura | 2022 | *OFID* | Multisystem Inflammatory Syndrome in Adults and Severe Toxoplasmosis: Similar Clinical Presentations, Potentially Severe Outcomes | Karius DC3; modified Omega BioTek Mag-Bind cfDNA (bead) | Karius DC3; modified Tecan Ovation Ultralow System V2 (ds) |
| **VAL-C** | Guo | 2022 | *BMC Nephrology* | Bartonella endocarditis and diffuse crescentic proliferative glomerulonephritis with a full-house pattern of immune complex deposition | Karius DC3; modified Omega BioTek Mag-Bind cfDNA (bead) | Karius DC3; modified Tecan Ovation Ultralow System V2 (ds) |
| **VAL-C** | Osman | 2022 | *Cureus* | Antisynthetase Syndrome Post Shingrix and Pneumovax Vaccinations, Possible Correlation | Karius DC3; modified Omega BioTek Mag-Bind cfDNA (bead) | Karius DC3; modified Tecan Ovation Ultralow System V2 (ds) |
| **VAL-C** | Xu | 2022 | *Proceedings of UCLA Health* | Curious Case of Cunninghamella Diagnosed Utilizing Metagenomic Next-Generation Sequencing | Karius DC3; modified Omega BioTek Mag-Bind cfDNA (bead) | Karius DC3; modified Tecan Ovation Ultralow System V2 (ds) |
| **VAL-C** | Luu | 2022 | *Cureus* | Disseminated Nocardia paucivorans Infection Resembling Metastatic Disease in a Kidney Transplant Recipient | Karius DC3; modified Omega BioTek Mag-Bind cfDNA (bead) | Karius DC3; modified Tecan Ovation Ultralow System V2 (ds) |
| **VAL-C** | Provenzano | 2022 | *Cureus* | An Unusual Case of Prosthetic Valve Endocarditis | Karius DC3; modified Omega BioTek Mag-Bind cfDNA (bead) | Karius DC3; modified Tecan Ovation Ultralow System V2 (ds) |
| **VAL-C** | Abe | 2022 | *J Pediatric Infect Dis Soc* | A Rare Etiology for Ascending Paralysis in an Infant | Karius DC3; modified Omega BioTek Mag-Bind cfDNA (bead) | Karius DC3; modified Tecan Ovation Ultralow System V2 (ds) |
| **VAL-C** | Jain | 2022 | *Neurohospitalist* | A 40-Year-Old Woman With COVID-19 and Bilateral Vision Loss | Karius DC3; modified Omega BioTek Mag-Bind cfDNA (bead) | Karius DC3; modified Tecan Ovation Ultralow System V2 (ds) |
| **VAL-C** | Vadhan | 2022 | *JECCM* | Fast and Fusariosis: a systematic review and case report of a rapidly fatal central nervous system infection | Karius DC3; modified Omega BioTek Mag-Bind cfDNA (bead) | Karius DC3; modified Tecan Ovation Ultralow System V2 (ds) |
| **VAL-C** | Srichawla | 2022 | *Cureus* | Plasma Microbial Cell-Free DNA (CF-DNA) Next-Generation Sequencing in Diagnosing Intracranial Abscesses: Pathophysiology and a Scoping Review of the Literature | Karius DC3; modified Omega BioTek Mag-Bind cfDNA (bead) | Karius DC3; modified Tecan Ovation Ultralow System V2 (ds) |
| **VAL-C** | Boguniewicz | 2022 | *Pediatr Infect Dis J* | Management of Extensive Central Nervous System Cladophialophora bantiana Infection in a 9-Year-Old Child | Karius DC3; modified Omega BioTek Mag-Bind cfDNA (bead) | Karius DC3; modified Tecan Ovation Ultralow System V2 (ds) |
| **VAL-C** | Chang | 2022 | *Front. Pediatr.* | Case report: Disseminated histoplasmosis in a renal transplant recipient from a non-endemic region | Karius DC3; modified Omega BioTek Mag-Bind cfDNA (bead) | Karius DC3; modified Tecan Ovation Ultralow System V2 (ds) |
| **VAL-C** | Naser | 2022 | *NEJM* | Hiding in Plain Sight | Karius DC3; modified Omega BioTek Mag-Bind cfDNA (bead) | Karius DC3; modified Tecan Ovation Ultralow System V2 (ds) |
| **VAL-C** | Rodriguez | 2022 | *Front Pediatr* | Plasma cell free next-generation sequencing detects an unusual pneumonia pathogen in an immunocompetent adolescent with acute respiratory distress syndrome | Karius DC3; modified Omega BioTek Mag-Bind cfDNA (bead) | Karius DC3; modified Tecan Ovation Ultralow System V2 (ds) |
| **VAL-C** | Bégué | 2022 | *Pediatr Infect Dis J* | An 11-Year-old Boy With Fever and Rash | Karius DC3; modified Omega BioTek Mag-Bind cfDNA (bead) | Karius DC3; modified Tecan Ovation Ultralow System V2 (ds) |
| **VAL-C** | Shaffer | 2023 | *J Neurosurgery* | Disseminated blastomycosis with an intracranial fungoma in an immunocompetent patient: illustrative case | Karius DC3; modified Omega BioTek Mag-Bind cfDNA (bead) | Karius DC3; modified Tecan Ovation Ultralow System V2 (ds) |
| **VAL-C** | Henien | 2023 | *Cureus* | Prosthetic Valve Infective Endocarditis Secondary to Neisseria elongata | Karius DC3; modified Omega BioTek Mag-Bind cfDNA (bead) | Karius DC3; modified Tecan Ovation Ultralow System V2 (ds) |
| **VAL-C** | Venigalla | 2023 | *Curr. Oncol.* | An Unusual Case of Hemophagocytic Lymphohistiocytosis Associated with Mycobacterium chimaera or Large-Cell Neuroendocrine Carcinoma | Karius DC3; modified Omega BioTek Mag-Bind cfDNA (bead) | Karius DC3; modified Tecan Ovation Ultralow System V2 (ds) |
| **VAL-C** | Rubio | 2023 | *Emerg Infect Dis* | Borrelia miyamotoi Infection in Immunocompromised Man, California, USA, 2021 | Karius DC3; modified Omega BioTek Mag-Bind cfDNA (bead) | Karius DC3; modified Tecan Ovation Ultralow System V2 (ds) |
| **VAL-C** | Im | 2023 | *Cureus* | Lemierre’s Syndrome Associated With Papillary Thyroid Cancer and Cerebellar Stroke | Karius DC3; modified Omega BioTek Mag-Bind cfDNA (bead) | Karius DC3; modified Tecan Ovation Ultralow System V2 (ds) |
| **VAL-C** | Smith | 2023 | *Pediatr Pulmonol* | Karius test as a noninvasive diagnostic tool in pediatric hematopoietic stem cell transplant patients with pneumonia: A case series | Karius DC3; modified Omega BioTek Mag-Bind cfDNA (bead) | Karius DC3; modified Tecan Ovation Ultralow System V2 (ds) |
| **VAL-C** | McBee | 2023 | *Cureus* | A Case of Severe, Difficult-to-Diagnose Legionnaires' Disease in a Young Welder | Karius DC3; modified Omega BioTek Mag-Bind cfDNA (bead) | Karius DC3; modified Tecan Ovation Ultralow System V2 (ds) |
| **VAL-C** | Bisarya | 2023 | *Kansas Journal of Medicine* | A Case of Catastrophic Aspergillus Endocarditis | Karius DC3; modified Omega BioTek Mag-Bind cfDNA (bead) | Karius DC3; modified Tecan Ovation Ultralow System V2 (ds) |
| **VAL-C** | Carlan | 2023 | *Jour Clin Med Res* | Next-generation sequencing of microbial cell-free DNA to rapidly detect Fluoribacter bozemanae pneunomia in an immunocompromised host | Karius DC3; modified Omega BioTek Mag-Bind cfDNA (bead) | Karius DC3; modified Tecan Ovation Ultralow System V2 (ds) |
| **VAL-C** | Sircar | 2023 | *BMJ Case Rep* | Disseminated histoplasmosis in an immunosuppressed patient successfully treated with isavuconazole | Karius DC3; modified Omega BioTek Mag-Bind cfDNA (bead) | Karius DC3; modified Tecan Ovation Ultralow System V2 (ds) |
| **VAL-C** | Butler | 2023 | *Cureus* | An Evasive Case of Gonococcal Endocarditis | Karius DC3; modified Omega BioTek Mag-Bind cfDNA (bead) | Karius DC3; modified Tecan Ovation Ultralow System V2 (ds) |
| **VAL-C** | Chatterjee | 2023 | *Germs* | Finding of Anaerococcus hydrogenalis in blood using cell-free DNA technique in a patient with infective endocarditis | Karius DC3; modified Omega BioTek Mag-Bind cfDNA (bead) | Karius DC3; modified Tecan Ovation Ultralow System V2 (ds) |
| **VAL-C** | Aldawsari | 2023 | *IDCases* | Infective intracardiac lesion in the setting of Mycobacterium franklinii bacteremia identified by cell-free DNA sequencing in a child with acute lymphoblastic leukemia: A potential new foe in intracardiac infections | Karius DC3; modified Omega BioTek Mag-Bind cfDNA (bead) | Karius DC3; modified Tecan Ovation Ultralow System V2 (ds) |
| **VAL-C** | Mondy | 2023 | *Infect Dis Clin Pract* | Rapid Diagnosis of Bartonella-Induced Hemophagocytic Lymphohistiocytosis Using Next-Generation Sequencing of Plasma | Karius DC3; modified Omega BioTek Mag-Bind cfDNA (bead) | Karius DC3; modified Tecan Ovation Ultralow System V2 (ds) |
| **VAL-C** | Nasir | 2023 | *BMJ Case Rep* | Metagenomic next-generation sequencing identifying a rare case of Mycobacterium xenopi discitis | Karius DC3; modified Omega BioTek Mag-Bind cfDNA (bead) | Karius DC3; modified Tecan Ovation Ultralow System V2 (ds) |
| **VAL-C** | Herskovitz | 2023 | *GMERJ* | Rare Streptococcus intermedius Central Nervous System Septic Emboli: A Case Report and Review of Literature | Karius DC3; modified Omega BioTek Mag-Bind cfDNA (bead) | Karius DC3; modified Tecan Ovation Ultralow System V2 (ds) |
| **VAL-C** | Goren | 2023 | *Cureus* | Possible Donor-Derived Infection in a Pediatric Liver Transplant Patient With Granulomatous Hepatitis | Karius DC3; modified Omega BioTek Mag-Bind cfDNA (bead) | Karius DC3; modified Tecan Ovation Ultralow System V2 (ds) |
| **VAL-C** | Dennis | 2023 | *J Pediatr Health Care* | Use of Metagenomic Next-Generation Sequencing in the Identification of Pneumocystis Jiroveci Pneumonia in a Previously Healthy Infant Diagnosed With X-Linked Hyper-IgM Syndrome | Karius DC3; modified Omega BioTek Mag-Bind cfDNA (bead) | Karius DC3; modified Tecan Ovation Ultralow System V2 (ds) |
| **VAL-C** | Olivo-Freites | 2023 | *Am J Trop Med Hyg* | Case Report: Cardiovascular Manifestations Due to Flea-Borne Typhus | Karius DC3; modified Omega BioTek Mag-Bind cfDNA (bead) | Karius DC3; modified Tecan Ovation Ultralow System V2 (ds) |
| **VAL-C** | Sahra | 2023 | *BMC Infectious Diseases* | The diagnostic dilemma for atypical presentation of progressive human Mpox | Karius DC3; modified Omega BioTek Mag-Bind cfDNA (bead) | Karius DC3; modified Tecan Ovation Ultralow System V2 (ds) |
| **VAL-C** | Traver | 2024 | *Emerg Infect Dis* | Severe Infective Endocarditis Caused by Bartonella rochalimae | Karius DC3; modified Omega BioTek Mag-Bind cfDNA (bead) | Karius DC3; modified Tecan Ovation Ultralow System V2 (ds) |
| **VAL-C** | Scheidt | 2024 | *JSES Reviews, Reports, and Techniques* | Culture Negative Septic Glenohumeral Arthritis Identified with Plasma Microbial Cell-Free DNA Sequencing: A Case Report | Karius DC3; modified Omega BioTek Mag-Bind cfDNA (bead) | Karius DC3; modified Tecan Ovation Ultralow System V2 (ds) |
| **VAL-C** | Rutenberg | 2024 | *NEJM* | The Meat of the Matter | Karius DC3; modified Omega BioTek Mag-Bind cfDNA (bead) | Karius DC3; modified Tecan Ovation Ultralow System V2 (ds) |
| **VAL-C** | Caldararo | 2022 | *Infect Dis Clin Pract* | Atypical Pathogens Presenting With Pulmonary Consolidations Detected by Cell-Free DNA Next-Generation Sequencing in Patients with Hematologic Malignancies | Karius DC3; modified Omega BioTek Mag-Bind cfDNA (bead) | Karius DC3; modified Tecan Ovation Ultralow System V2 (ds) |
| **VAL-C** | Ogunsiakan | 2022 | *Transplantation Direct* | Unusual Presentation of Disseminated Mycobacterium kansasii Infection in Renal Transplant Recipients and Rapid Diagnosis Using Plasma Microbial Cell-free DNA Next-generation Sequencing | Karius DC3; modified Omega BioTek Mag-Bind cfDNA (bead) | Karius DC3; modified Tecan Ovation Ultralow System V2 (ds) |
| **VAL-C** | Weiss | 2023 | *Diagn Microbiol Infect Dis* | The diagnostic and clinical utility of microbial cell-free DNA sequencing in a real-world setting | Karius DC3; modified Omega BioTek Mag-Bind cfDNA (bead) | Karius DC3; modified Tecan Ovation Ultralow System V2 (ds) |
| **VAL-C** | David | 2023 | *Epidemiol Infect* | Cell-free plasma next-generation sequencing assists in the evaluation of secondary pneumonia in patients with COVID-19: a case series | Karius DC3; modified Omega BioTek Mag-Bind cfDNA (bead) | Karius DC3; modified Tecan Ovation Ultralow System V2 (ds) |
| **VAL-C** | Nguyen-Tran | 2023 | *Pediatric Infectious Disease Journal* | Use of Advanced Diagnostics for Timely Identification of Travel-associated Leptospira santarosai Infection in Four Adolescents Through Plasma Microbial Cell-free DNA Sequencing With the Karius Test | Karius DC3; modified Omega BioTek Mag-Bind cfDNA (bead) | Karius DC3; modified Tecan Ovation Ultralow System V2 (ds) |
| **VAL-C** | Centeno | 2024 | *Antimicrobial Stewardship & Healthcare Epidemiology* | Detection of invasive Bartonella infections with next-generation sequencing of microbial cell-free DNA | Karius DC3; modified Omega BioTek Mag-Bind cfDNA (bead) | Karius DC3; modified Tecan Ovation Ultralow System V2 (ds) |
| **VAL-C** | Li | 2024 | *Journal of Clinical Tuberculosis and Other*  *Mycobacterial Diseases* | Cell-free DNA blood test for the diagnosis of pediatric tuberculous meningitis | Karius DC3; modified Omega BioTek Mag-Bind cfDNA (bead) | Karius DC3; modified Tecan Ovation Ultralow System V2 (ds) |
| **VAL-C** | Garcia | 2024 | *Pediatr Infect Dis J* | Emerging Role of Plasma Microbial Cell-free DNA in the Diagnosis of Pediatric Mucormycosis | Karius DC3; modified Omega BioTek Mag-Bind cfDNA (bead) | Karius DC3; modified Tecan Ovation Ultralow System V2 (ds) |
| **VAL-C** | Scheidt | 2024 | *JSES Reviews, Reports, and Techniques* | Culture Negative Septic Glenohumeral Arthritis Identified with Plasma Microbial Cell-Free DNA Sequencing: A Case Report | Karius DC3; modified Omega BioTek Mag-Bind cfDNA (bead) | Karius DC3; modified Tecan Ovation Ultralow System V2 (ds) |
| **VAL-C** | Rutenberg | 2024 | *NEJM* | The Meat of the Matter | Karius DC3; modified Omega BioTek Mag-Bind cfDNA (bead) | Karius DC3; modified Tecan Ovation Ultralow System V2 (ds) |
| **VAL-C** | Cash-Goldwasser | 2024 | *CDC MMWR* | Outbreak of Human Trichinellosis — Arizona, Minnesota, and South Dakota, 2022 | Karius DC3; modified Omega BioTek Mag-Bind cfDNA (bead) | Karius DC3; modified Tecan Ovation Ultralow System V2 (ds) |
| **VAL-C** | Blackbourn | 2024 | *IDCases* | Human Herpes Virus-6 (HHV-6) infectious encephalitis in an immunocompetent adult | Karius DC3; modified Omega BioTek Mag-Bind cfDNA (bead) | Karius DC3; modified Tecan Ovation Ultralow System V2 (ds) |
| **VAL-C** | Probert | 2024 | *EID* | Newly Recognized Spotted Fever Group Rickettsia as Cause of Severe Rocky Mountain Spotted Fever–Like Illness, Northern California, USA | Karius DC3; modified Omega BioTek Mag-Bind cfDNA (bead) | Karius DC3; modified Tecan Ovation Ultralow System V2 (ds) |
| **VAL-C** | Iyyani | 2024 | *American Journal of Case Reports* | Rare case of disseminated histoplasmosis mitral valve endocarditis in Florida | Karius DC3; modified Omega BioTek Mag-Bind cfDNA (bead) | Karius DC3; modified Tecan Ovation Ultralow System V2 (ds) |
| **VAL-C** | Onyishi | 2024 | *Cureus* | Invasive Community-Acquired Methicillin-Resistant Staphylococcus aureus With Aortic Aneurysm in a 10-Year-Old Patient: A Case Report | Karius DC3; modified Omega BioTek Mag-Bind cfDNA (bead) | Karius DC3; modified Tecan Ovation Ultralow System V2 (ds) |
| **VAL-C** | Ghoussaini | 2024 | *IDCases* | Graft versus histoplasma disease: A case of vascular graft infection | Karius DC3; modified Omega BioTek Mag-Bind cfDNA (bead) | Karius DC3; modified Tecan Ovation Ultralow System V2 (ds) |
| **VAL-C** | Phadke | 2024 | *Emerg Infect Dis* | Rickettsia parkeri Rickettsiosis in Kidney Transplant Recipient, North Carolina, USA, 2023 | Karius DC3; modified Omega BioTek Mag-Bind cfDNA (bead) | Karius DC3; modified Tecan Ovation Ultralow System V2 (ds) |
| **VAL-C** | Mann | 2024 | *Trans Infect Dis* | Emerging shadows: HHV-8-associated encephalitis unveiled in a solid organ transplant recipient | Karius DC3; modified Omega BioTek Mag-Bind cfDNA (bead) | Karius DC3; modified Tecan Ovation Ultralow System V2 (ds) |
| **VAL-C** | Bhatia-Lin | 2024 | *Proceedings of UCLA Health* | Rhabdomyolysis with Central Nervous System Lesions due to Legionnaire’s Disease: A Mystery Solved by Deep-Sequencing | Karius DC3; modified Omega BioTek Mag-Bind cfDNA (bead) | Karius DC3; modified Tecan Ovation Ultralow System V2 (ds) |
| **VAL-C** | Alzahrani | 2024 | *Pediatric Dermatology* | Cutaneous legionellosis in an immunocompromised neonate | Karius DC3; modified Omega BioTek Mag-Bind cfDNA (bead) | Karius DC3; modified Tecan Ovation Ultralow System V2 (ds) |
| **VAL-C** | Huang | 2024 | *Proceedings of UCLA Health* | Legionnaires’ Disease Presenting as Encephalopathy in a Returning Traveler: A Diagnostic Dilemma | Karius DC3; modified Omega BioTek Mag-Bind cfDNA (bead) | Karius DC3; modified Tecan Ovation Ultralow System V2 (ds) |
| **VAL-C** | Rentia | 2024 | *Surgical Neurology International* | A rare case of Streptobacillus moniliformis epidural abscess requiring neurosurgical decompression | Karius DC3; modified Omega BioTek Mag-Bind cfDNA (bead) | Karius DC3; modified Tecan Ovation Ultralow System V2 (ds) |
| **VAL-C** | Cummings | 2024 | *BMJ Case Rep* | Tricuspid valve infective endocarditis in disseminated gonococcal infection (DGI) | Karius DC3; modified Omega BioTek Mag-Bind cfDNA (bead) | Karius DC3; modified Tecan Ovation Ultralow System V2 (ds) |
| **VAL-C** | Jang | 2024 | *Diagn Microbiol Infect Dis* | Ehrlichiosis and Anaplasmosis in Solid Organ Transplantation: A Case Series and Review of the Literature | Karius DC3; modified Omega BioTek Mag-Bind cfDNA (bead) | Karius DC3; modified Tecan Ovation Ultralow System V2 (ds) |
| **VAL-C** | Hirth | 2024 | *BMJ Case Rep* | Usefulness of newer testing modalities for the accurate diagnosis of culture-negative endocarditis | Karius DC3; modified Omega BioTek Mag-Bind cfDNA (bead) | Karius DC3; modified Tecan Ovation Ultralow System V2 (ds) |
| **VAL-C** | Whitman | 2024 | *Case Rep Transplant* | A Rare Case of Cladophialophora bantiana Intracranial Infection: Highlighting the Utility of Next-Generation Sequencing in Diagnosis | Karius DC3; modified Omega BioTek Mag-Bind cfDNA (bead) | Karius DC3; modified Tecan Ovation Ultralow System V2 (ds) |
| **VAL-C** | Szatkowski | 2024 | *Proceedings of UCLA Health* | Lemierre’s Syndrome and Diagnostic Utility of Cell Free DNA Analysis | Karius DC3; modified Omega BioTek Mag-Bind cfDNA (bead) | Karius DC3; modified Tecan Ovation Ultralow System V2 (ds) |
| **VAL-C** | Nirmal | 2024 | *Cureus* | Culture-Negative Endocarditis Complicated by Cerebral Abscesses Due to Streptococcus gordonii: A Diagnostic Odyssey | Karius DC3; modified Omega BioTek Mag-Bind cfDNA (bead) | Karius DC3; modified Tecan Ovation Ultralow System V2 (ds) |
| **VAL-C** | Chiang | 2024 | *Emerg Infect Dis* | Rocky Mountain Spotted Fever in Children along the US‒Mexico Border, 2017–2023 | Karius DC3; modified Omega BioTek Mag-Bind cfDNA (bead) | Karius DC3; modified Tecan Ovation Ultralow System V2 (ds) |
| **VAL-C** | Atkin | 2024 | *Am Surg* | A Rare Case of Disseminated Strongyloidiasis Leading to Multisystem Organ Failure Following Deceased Donor Kidney Transplantation | Karius DC3; modified Omega BioTek Mag-Bind cfDNA (bead) | Karius DC3; modified Tecan Ovation Ultralow System V2 (ds) |
| **VAL-C** | Pearson | 2024 | *Cureus* | Concurrent Zoster Sine Herpete and Hepatosplenic Fungal Infection in a Cancer Patient: A Case Report | Karius DC3; modified Omega BioTek Mag-Bind cfDNA (bead) | Karius DC3; modified Tecan Ovation Ultralow System V2 (ds) |
| **VAL-C** | Hennis | 2024 | *Cureus* | Use of Cell-Free DNA Testing to Diagnose Infective Endocarditis in a Patient With Negative Blood Cultures | Karius DC3; modified Omega BioTek Mag-Bind cfDNA (bead) | Karius DC3; modified Tecan Ovation Ultralow System V2 (ds) |
| **VAL-C** | Smith | 2024 | *BMJ Case Rep* | Karius testing in the identification of Coxiella burnetii infective endocarditis presenting with acute ischaemic stroke | Karius DC3; modified Omega BioTek Mag-Bind cfDNA (bead) | Karius DC3; modified Tecan Ovation Ultralow System V2 (ds) |
| **VAL-C** | Rogers | 2024 | *CID* | Fatal borealpox in an immunosuppressed patient treated with antivirals and vaccinia immunoglobulin — Alaska, 2023 | Karius DC3; modified Omega BioTek Mag-Bind cfDNA (bead) | Karius DC3; modified Tecan Ovation Ultralow System V2 (ds) |
| **VAL-C** | Chetrit | 2024 | *Cureus* | The Use of Next-Generation Sequencing to Assist in the Diagnosis of Atypical Vasculitis | Karius DC3; modified Omega BioTek Mag-Bind cfDNA (bead) | Karius DC3; modified Tecan Ovation Ultralow System V2 (ds) |
| **VAL-C** | Chance | 2024 | *JPIDS* | Angiostrongylus cantonensis Meningoencephalitis in Three Pediatric Patients in Florida, USA | Karius DC3; modified Omega BioTek Mag-Bind cfDNA (bead) | Karius DC3; modified Tecan Ovation Ultralow System V2 (ds) |
| **VAL-C** | Vo | 2024 | *PIDJ* | MYOPERICARDITIS DUE TO COXIELLA BURNETII IN A TEENAGE MALE | Karius DC3; modified Omega BioTek Mag-Bind cfDNA (bead) | Karius DC3; modified Tecan Ovation Ultralow System V2 (ds) |
| **VAL-C** | Kjemtrup | 2024 | *MMWR Morb Mortal Wkly Rep* | Severe and Fatal Rocky Mountain Spotted Fever After Exposure in Tecate, Mexico - California, July 2023-January 2024 | Karius DC3; modified Omega BioTek Mag-Bind cfDNA (bead) | Karius DC3; modified Tecan Ovation Ultralow System V2 (ds) |
| **VAL-C** | Tang | 2024 | *Cureus* | Capnocytophaga Meningitis: A Rare Case of Bacterial Infection With Neurological Manifestation | Karius DC3; modified Omega BioTek Mag-Bind cfDNA (bead) | Karius DC3; modified Tecan Ovation Ultralow System V2 (ds) |
| **VAL-C** | Steinberger | 2024 | *Ophthalmic Plast Reconstr Surg* | An Atypical Mpox Presentation With a Large, Chronic Right Upper Eyelid Ulcerative Mass | Karius DC3; modified Omega BioTek Mag-Bind cfDNA (bead) | Karius DC3; modified Tecan Ovation Ultralow System V2 (ds) |
| **VAL-C** | Bergeron | 2024 | *IDCases* | Intracellular but not Undetectable: A Case of Francisella tularensis Pericarditis | Karius DC3; modified Omega BioTek Mag-Bind cfDNA (bead) | Karius DC3; modified Tecan Ovation Ultralow System V2 (ds) |
| **VAL-C** | Chambers | 2025 | *IDCP* | Non-Pneuomophila Legionnaires' Disease in Cancer Patients - A Case Series and Review of Literature | Karius DC3; modified Omega BioTek Mag-Bind cfDNA (bead) | Karius DC3; modified Tecan Ovation Ultralow System V2 (ds) |
| **VAL-C** | Chen | 2025 | *Cureus* | Mycobacterium immunogenum-Induced Thrombocytopenia | Karius DC3; modified Omega BioTek Mag-Bind cfDNA (bead) | Karius DC3; modified Tecan Ovation Ultralow System V2 (ds) |
| **VAL-C** | Kumar | 2025 | *Cureus* | Fever of Unknown Origin in a Young Woman With Multiple Comorbidities: A Diagnostic Challenge | Karius DC3; modified Omega BioTek Mag-Bind cfDNA (bead) | Karius DC3; modified Tecan Ovation Ultralow System V2 (ds) |
| **VAL-C** | Li | 2025 | *Cureus* | A Perplexing Case Highlighting the Diagnostic Conundrum of Miliary Tuberculosis Mimicking Sarcoidosis and Progressing Into Hemophagocytic Lymphohistiocytosis | Karius DC3; modified Omega BioTek Mag-Bind cfDNA (bead) | Karius DC3; modified Tecan Ovation Ultralow System V2 (ds) |
| **VAL-C** | Boopathiraj | 2025 | *Retinal Cases & Brief Reports* | Chorioretinitis following Bacillus Calmette-Guerin Treatment for Bladder Cancer | Karius DC3; modified Omega BioTek Mag-Bind cfDNA (bead) | Karius DC3; modified Tecan Ovation Ultralow System V2 (ds) |
| **VAL-C** | Vazquez | 2025 | *IDCases* | The cruciality of increasing index of suspicion for atypical Bartonella henselae in pediatric patients: A case series | Karius DC3; modified Omega BioTek Mag-Bind cfDNA (bead) | Karius DC3; modified Tecan Ovation Ultralow System V2 (ds) |
| **VAL-C** | Edminster | 2025 | *Acta Neuropathol Commun* | The role of plasma metagenomic sequencing in identification of Balamuthia mandrillaris encephalitis | Karius DC3; modified Omega BioTek Mag-Bind cfDNA (bead) | Karius DC3; modified Tecan Ovation Ultralow System V2 (ds) |
| **VAL-C** | Malaussena | 2025 | *Transplant Immunology* | Mycobacterium haemophilum diagnosed via Karius test in a heart transplant recipient: A case report | Karius DC3; modified Omega BioTek Mag-Bind cfDNA (bead) | Karius DC3; modified Tecan Ovation Ultralow System V2 (ds) |
| **VAL-C** | Sharma | 2025 | *Ann Vasc Surg* | Aortic Endograft infection with Mycobacterium bovis following intravesical BCG immunotherapy diagnosed via The Karius Test™- Case Report and review of literature | Karius DC3; modified Omega BioTek Mag-Bind cfDNA (bead) | Karius DC3; modified Tecan Ovation Ultralow System V2 (ds) |
| **VAL-C** | Yu | 2025 | *Emerg Infect Dis* | Bartonella quintana Endocarditis and Pauci-Immune Glomerulonephritis in Patient without Known Risk Factors, USA, 2024 | Karius DC3; modified Omega BioTek Mag-Bind cfDNA (bead) | Karius DC3; modified Tecan Ovation Ultralow System V2 (ds) |
| **VAL-C** | Hansen | 2025 | *Pediatr Transplant* | Management of Trichodysplasia Spinulosa in a Pediatric Kidney Transplant Patient With Topical Cidofovir and Oral Leflunomide | Karius DC3; modified Omega BioTek Mag-Bind cfDNA (bead) | Karius DC3; modified Tecan Ovation Ultralow System V2 (ds) |
| **VAL-C** | Xue | 2025 | *Infectious Medicine* | Culture-negative liver abscess identified with plasma microbial cell-free DNA sequencing: A case report | Karius DC3; modified Omega BioTek Mag-Bind cfDNA (bead) | Karius DC3; modified Tecan Ovation Ultralow System V2 (ds) |
| **VAL-C** | Patel | 2025 | *Cureus* | Histoplasma capsulatum Prosthetic Valve Endocarditis Diagnosed via Cell-Free DNA Sequencing | Karius DC3; modified Omega BioTek Mag-Bind cfDNA (bead) | Karius DC3; modified Tecan Ovation Ultralow System V2 (ds) |
| **VAL-C** | Ko | 2025 | *Cureus* | Purpura Fulminans Secondary to Haemophilusin fluenzae in an Infant | Karius DC3; modified Omega BioTek Mag-Bind cfDNA (bead) | Karius DC3; modified Tecan Ovation Ultralow System V2 (ds) |
| **VAL-C** | Iben | 2025 | *Cureus* | Disseminated Nocardiosis: Poorly Controlled Diabetes as an Immunocompromising State | Karius DC3; modified Omega BioTek Mag-Bind cfDNA (bead) | Karius DC3; modified Tecan Ovation Ultralow System V2 (ds) |
| **VAL-C** | Sorensen | 2025 | *Cureus* | Severe Capnocytophaga Canimorsus Purpura Fulminans After a Cat Bite in an Asplenic Patient: Diagnostic and Therapeutic Challenges | Karius DC3; modified Omega BioTek Mag-Bind cfDNA (bead) | Karius DC3; modified Tecan Ovation Ultralow System V2 (ds) |
| **VAL-C** | Begovic | 2025 | *Cureus* | Clinical Application of Metagenomic Next-Generation Sequencing of Microbial Cell-free DNA in Ruling Out Invasive Fungal Infection in a Patient with Thermal Burn Wounds: A Case Report | Karius DC3; modified Omega BioTek Mag-Bind cfDNA (bead) | Karius DC3; modified Tecan Ovation Ultralow System V2 (ds) |
| **VAL-C** | Ladines-Lim | 2025 | *ASM Case Rep* | Delayed diagnosis of disseminated Mycobacterium intracellulare subsp. chimaera infective endocarditis via cell-free metagenomic next-generation sequencing: a case report | Karius DC3; modified Omega BioTek Mag-Bind cfDNA (bead) | Karius DC3; modified Tecan Ovation Ultralow System V2 (ds) |
| **VAL-C** | Plazola | 2025 | *Case Reports Infez Med.* | Aspergillus fumigatus endocarditis in an immunocompetent host aided by metagenomic next-generation sequencing assay: case report and literature review | Karius DC3; modified Omega BioTek Mag-Bind cfDNA (bead) | Karius DC3; modified Tecan Ovation Ultralow System V2 (ds) |
| **VAL-C** | Franco | 2025 | *Cureus* | Legionella maceachernii Pneumonia Presenting as a Cavitary Lesion in an Immunocompromised Patient | Karius DC3; modified Omega BioTek Mag-Bind cfDNA (bead) | Karius DC3; modified Tecan Ovation Ultralow System V2 (ds) |
| **VAL-C** | Shah | 2025 | *Case Rep Images Infect Dis* | Implantable cardioverter-defibrillator infection by Streptococcus intermedius identified by next-generation sequencing | Karius DC3; modified Omega BioTek Mag-Bind cfDNA (bead) | Karius DC3; modified Tecan Ovation Ultralow System V2 (ds) |
| **VAL-C** | Fishman | 2025 | *American Journal of Transplantation* | Infectious disease surveillance and management in clinical xenotransplantation: Experience with the first human porcine kidney transplant | Karius DC3; modified Omega BioTek Mag-Bind cfDNA (bead) | Karius DC3; modified Tecan Ovation Ultralow System V2 (ds) |
| **VAL-C** | Vega | 2025 | *OFID* | Donor-Derived Tuberculosis In Three Solid Organ Transplant Recipients From The Same Donor | Karius DC3; modified Omega BioTek Mag-Bind cfDNA (bead) | Karius DC3; modified Tecan Ovation Ultralow System V2 (ds) |
| **VAL-C** | Thalner | 2025 | *Progress in Pediatric Cardiology* | Histoplasma endocarditis in a prosthetic pulmonary valve: A case report | Karius DC3; modified Omega BioTek Mag-Bind cfDNA (bead) | Karius DC3; modified Tecan Ovation Ultralow System V2 (ds) |
| **VAL-C** | Issa | 2025 | *IDCases* | Whipple’s endocarditis diagnosed non-invasively with plasma microbial cell-free DNA sequencing | Karius DC3; modified Omega BioTek Mag-Bind cfDNA (bead) | Karius DC3; modified Tecan Ovation Ultralow System V2 (ds) |
| **VAL-C** | Lampou | 2025 | *Diagnostics* | Clinical Utility of Plasma Microbial Cell-Free DNA Surveillance in Neutropenic Patients with Acute Myeloid Leukemia Undergoing Outpatient Chemotherapy: A Case Series | Karius DC3; modified Omega BioTek Mag-Bind cfDNA (bead) | Karius DC3; modified Tecan Ovation Ultralow System V2 (ds) |
| **VAL-C** | Brandon | 2025 | *Ther Adv Infect Dis* | Mycoplasma hominis prosthetic valve infective endocarditis and endophthalmitis in a renal transplant recipient: a case report | Karius DC3; modified Omega BioTek Mag-Bind cfDNA (bead) | Karius DC3; modified Tecan Ovation Ultralow System V2 (ds) |
| **VAL-C** | Ma | 2025 | *Cureus* | Brain Abscess Complicating Metastatic Scalp Basal Cell Carcinoma With a Malignant Fungating Wound: A Case Report and Literature Review | Karius DC3; modified Omega BioTek Mag-Bind cfDNA (bead) | Karius DC3; modified Tecan Ovation Ultralow System V2 (ds) |
| **VAL-C** | Hasbani | 2025 | *EJCRIM* | A case of pneumococcal periaortitis: periaortitis is not always autoimmune | Karius DC3; modified Omega BioTek Mag-Bind cfDNA (bead) | Karius DC3; modified Tecan Ovation Ultralow System V2 (ds) |
| **VAL-C** | Lee | 2025 | *Ophthalmic Plast Reconstr Surg* | Ophthalmic Vein Thrombosis, Abducens Nerve Palsy, and Horner Syndrome Secondary to Lemierre Synd | Karius DC3; modified Omega BioTek Mag-Bind cfDNA (bead) | Karius DC3; modified Tecan Ovation Ultralow System V2 (ds) |
| **VAL-C** | Tran | 2025 | *BMJ Neurol Open* | Plasma cell-free DNA testing in diagnosing Listeria rhombencephalitis in a CSF PCR-negative patient: a case report | Karius DC3; modified Omega BioTek Mag-Bind cfDNA (bead) | Karius DC3; modified Tecan Ovation Ultralow System V2 (ds) |
| **VAL-C** | Phillips | 2025 | *ASM Case Rep* | Trichosporon inkin meningitis in a pediatric patient diagnosed via metagenomic sequencing | Karius DC3; modified Omega BioTek Mag-Bind cfDNA (bead) | Karius DC3; modified Tecan Ovation Ultralow System V2 (ds) |
| **VAL-C** | Wells | 2025 | *Cureus* | Necrotizing Mediastinal Mass and Cavitary Pneumonia From Probable Co-infection With Epstein-Barr Virus and Mycoplasma pneumoniae | Karius DC3; modified Omega BioTek Mag-Bind cfDNA (bead) | Karius DC3; modified Tecan Ovation Ultralow System V2 (ds) |
| **VAL-C** | Kothiya | 2025 | *Infectious Diseases in Clinical Practice* | Hematogenous Dissemination of Mycobacterium abscessus Causing Pulmonary Nodules | Karius DC3; modified Omega BioTek Mag-Bind cfDNA (bead) | Karius DC3; modified Tecan Ovation Ultralow System V2 (ds) |
| **VAL-C** | Newstead | 2025 | *Ther Adv Infect Dis* | Disseminated Mycobacterium kansasii and Mycobacterium avium complex co-infection in GATA2 mutated myelodysplastic syndrome: a case report | Karius DC3; modified Omega BioTek Mag-Bind cfDNA (bead) | Karius DC3; modified Tecan Ovation Ultralow System V2 (ds) |
| **VAL-C** | Behinaein | 2025 | *JACC Case Rep* | Aortic Root Abscess Secondary to Coxiella burnetii Following TAVR: A Case Requiring the Hemi-Commando Procedure | Karius DC3; modified Omega BioTek Mag-Bind cfDNA (bead) | Karius DC3; modified Tecan Ovation Ultralow System V2 (ds) |
| **VAL-C** | Cohodes | 2025 | *Journal of Clinical Tuberculosis and Other Mycobacterial Diseases* | Curated cases from the TB expert network: Unplugged! Series: Use of plasma microbial cell-free DNA metagenomic sequencing to diagnose Mycobacterium tuberculosis | Karius DC3; modified Omega BioTek Mag-Bind cfDNA (bead) | Karius DC3; modified Tecan Ovation Ultralow System V2 (ds) |
| **VAL-C** | Jones | 2025 | *Emerg Infect Dis* | Organ Donor Transmission of Rickettsia typhi to Kidney Transplant Recipients, Texas, USA, 2024 | Karius DC3; modified Omega BioTek Mag-Bind cfDNA (bead) | Karius DC3; modified Tecan Ovation Ultralow System V2 (ds) |
| **VAL-C** | Allen | 2025 | *Cureus* | Atypical Presentations of Acyclovir-Resistant Herpes Simplex Virus in Immunocompromised Patients: A Case Series | Karius DC3; modified Omega BioTek Mag-Bind cfDNA (bead) | Karius DC3; modified Tecan Ovation Ultralow System V2 (ds) |

**Abbreviations:**

RUO: Research use only method / assay

VAL-C: Validated commercial method / assay (Case)

VAL-R: Validated commercial method / assay (Review)

VAL-S: Validated commercial method / assay (Study)

(col): Solid matrix column-based extraction

(bead): Magnetic bead-based extraction

(bead-S): Automated magnetic bead extraction system

(ds): Double-stranded DNA library preparation method

(ss/ds): Combined single-stranded and double-stranded library preparation method

**Supplementary Table 2.**

Sequencing output metrics of all sequencing runs performed for the four comparative library preparation chemistries.

| **Library Chemistry** | **Pools** | **Sequencing**  **Chemistry** | **Kit**  **Cycles** | **Read**  **Format** | **Yield**  **(Gbp)** | **Avg%**  **Q30** | **PF**  **Clusters** |
| --- | --- | --- | --- | --- | --- | --- | --- |
| **Comparative Runs** | | | | | | | |
| **Karius Helion-4** | 2 | NextSeq HO v2 | 150 | 76 x 76 PE | 175.3 | 90.4 | 1,071 M |
| **Karius DC3** | 2 | NextSeq HO v2 | 150 | 76 x 76 PE | 163.6 | 91.8 | 998 M |
| **Ext+ssDNA** | 2 | NextSeq HO v2 | 150 | 76 x 76 PE | 162.8 | 78.2 | 997 M |
| **Ext+dsDNA** | 2 | NextSeq HO v2 | 150 | 76 x 76 PE | 203.8 | 83.5 | 1,245 M |
| **Yield Calculation Runs (3% input, +5 cycles PCR)** | | | | | | | |
| **Karius Helion-4** | 2 | NextSeq HO v2 | 75 | 76 SE | 98.8 | 90.7 | 1,113 M |
| **Karius DC3** | 2 | NextSeq HO v2 | 75 | 76 SE | 92.0 | 92.1 | 1,036 M |
| **Ext+ssDNA** | 2 | NextSeq HO v2 | 75 | 76 SE | 110.7 | 89.5 | 1,249 M |
| **Ext+dsDNA** | 2 | NextSeq HO v2 | 75 | 76 SE | 94.6 | 94.0 | 1,062 M |

**Abbreviations:**

HO: High output

PF: Pass filtering (Illumina)

**Supplementary Table 3.**

Called microbes in 36 plasma samples processed with four comparator metagenomic sequencing approaches utilizing the mcfDNA analyte. Microbial species detected independently by at least two laboratory processes are highlighted in bold, while those identified by a single process are presented in regular font.

|  | **Called microbes** | | | | |
| --- | --- | --- | --- | --- | --- |
| **Sample ID** | **Karius Helion-4** | **Microbial Abundance (m/100nl)** | **Karius DC3** | **Ext+dsDNA** | **Ext+ssDNA** |
| plasma_001 | *Atopobium vaginae*  ***Bacteroides pyogenes***  *Escherichia coli*  ***Fusobacterium nucleatum***  ***Prevotella denticola***  ***Prevotella intermedia***  ***Prevotella oris*** | 259  **529**  3639  **2063**  **370**  **691**  **446** | ***Bacteroides pyogenes***  ***Fusobacterium nucleatum***  ***Prevotella denticola***  ***Prevotella intermedia***  ***Prevotella oris*** | No calls | No calls |
| plasma_002 | *Actinomyces graevenitzii*  *Actinomyces oris*  *Atopobium parvulum*  *Campylobacter concisus*  *Capnocytophaga ochracea*  *Corynebacterium matruchotii*  ***Escherichia coli***  *Fusobacterium nucleatum*  *Gemella sanguinis*  *Haemophilus parainfluenzae*  *Megasphaera micronuciformis*  ***Prevotella melaninogenica***  ***Rothia mucilaginosa***  *Streptococcus infantis*  *Streptococcus mitis*  *Streptococcus parasanguinis*  *Tannerella forsythia*  *Veillonella dispar*  *Veillonella parvula* | 145  161  88  119  54  325  **1049**  645  255  564  236  **2248**  **2746**  501  539  1708  348  764  269 | ***Escherichia coli***  ***Prevotella melaninogenica***  ***Rothia mucilaginosa*** | No calls | No calls |
| plasma_003 | ***Bacteroides fragilis***  ***Enterobacter cloacae complex***  ***Haemophilus parainfluenzae***  ***Neisseria flavescens***  ***Neisseria mucosa***  *Prevotella melaninogenica*  ***Rothia dentocariosa***  ***Rothia mucilaginosa***  ***Streptococcus oralis***  ***Veillonella dispar*** | **396**  **2139**  **1810**  **667**  **1957**  154  **258**  **153**  **1547**  467 | *Aeromonas caviae*  ***Bacteroides fragilis***  ***Enterobacter cloacae complex***  ***Haemophilus parainfluenzae***  ***Neisseria flavescens***  ***Neisseria mucosa***  ***Rothia dentocariosa***  ***Rothia mucilaginosa***  ***Streptococcus oralis***  ***Veillonella dispar*** | No calls | No calls |
| plasma_004 | No calls |  | *Anaeroglobus geminatus*  *Prevotella oris*  *[Enterobacter] aerogenes* | No calls | No calls |
| plasma_005 | ***Aspergillus nidulans***  ***Klebsiella pneumoniae***  ***Pseudomonas aeruginosa*** | **3263**  **808**  **262** | ***Aspergillus nidulans***  ***Klebsiella pneumoniae***  ***Pseudomonas aeruginosa*** | ***Aspergillus nidulans*** | ***Aspergillus nidulans*** |
| plasma_006 | ***Campylobacter concisus***  ***Cryptococcus neoformans***  ***Fusobacterium periodonticum***  ***Prevotella loescheii***  ***Prevotella melaninogenica***  ***Rothia mucilaginosa***  ***Veillonella dispar***  ***Veillonella parvula*** | **123**  **91**  **200**  **2772**  **2351**  **857**  **1426**  **2572** | ***Campylobacter concisus***  ***Cryptococcus neoformans***  ***Fusobacterium periodonticum***  ***Prevotella loescheii***  ***Prevotella melaninogenica***  ***Rothia mucilaginosa***  ***Veillonella dispar***  ***Veillonella parvula*** | No calls | ***Prevotella loescheii***  ***Prevotella melaninogenica*** |
| plasma_007 | ***Anaerococcus lactolyticus***  ***Atopobium rimae***  ***Bacteroides thetaiotaomicron***  ***Peptoniphilus harei***  ***Peptoniphilus lacrimalis***  ***Peptostreptococcus anaerobius***  *Peptostreptococcus stomatis*  ***Porphyromonas asaccharolytica***  ***Prevotella denticola***  *Prevotella nigrescens*  ***Pseudoramibacter alactolyticus*** | **13855**  **9377**  **4430**  **14424**  **11421**  **14838**  3738  **3777**  **90465**  25140  **11754** | ***Anaerococcus lactolyticus***  ***Atopobium rimae***  ***Bacteroides thetaiotaomicron***  ***Peptoniphilus harei***  ***Peptoniphilus lacrimalis***  ***Peptostreptococcus anaerobius***  ***Porphyromonas asaccharolytica***  ***Prevotella denticola***  ***Pseudoramibacter alactolyticus*** | No calls | ***Prevotella denticola*** |
| plasma_008 | No calls |  | No calls | No calls | No calls |
| plasma_009 | ***Actinomyces oris***  ***Enterococcus faecium***  ***Rothia dentocariosa***  ***Rothia mucilaginosa***  ***Streptococcus parasanguini***  ***Veillonella dispar***  ***Veillonella parvula*** | **278**  **482**  **152**  **89**  **713**  **406**  **158** | ***Actinomyces oris***  *Atopobium parvulum*  ***Enterococcus faecium***  ***Rothia dentocariosa***  ***Rothia mucilaginosa***  ***Streptococcus parasanguini***  ***Veillonella dispar***  ***Veillonella parvula*** | No calls | No calls |
| plasma_010 | *Escherichia coli*  *Human herpesvirus 4* | 322  116 | No calls | No calls | No calls |
| plasma_011 | ***Citrobacter freundii***  ***Human herpesvirus 4***  ***Serratia marcescens*** | **238**  **202**  **116** | ***Citrobacter freundii***  ***Human herpesvirus 4***  *Pseudomonas putida*  ***Serratia marcescens*** | No calls | No calls |
| plasma_012 | ***Candida parapsilosis***  ***Fusarium solani***  ***Fusarium sp. FSSC_6***  ***Nectria haematococca*** | **1189**  **4550**  **10450**  **8429** | ***Candida parapsilosis***  ***Fusarium solani***  ***Nectria haematococca*** | ***Fusarium solani***  ***Fusarium sp. FSSC_6***  ***Nectria haematococca*** | ***Candida parapsilosis***  ***Fusarium solani***  ***Fusarium sp. FSSC_6***  ***Nectria haematococca*** |
| plasma_013 | No calls |  | No calls | No calls | No calls |
| plasma_014 | ***Candida albicans***  ***Enterococcus faecalis***  ***Helicobacter pylori***  ***Veillonella dispar***  ***Veillonella parvula*** | **1031**  **3761**  **850**  **181**  **192** | *Actinomyces odontolyticus*  *Actinomyces oris*  ***Candida albicans***  ***Enterococcus faecalis***  ***Helicobacter pylori***  *Rothia dentocariosa*  ***Veillonella dispar***  ***Veillonella parvula*** | No calls | No calls |
| plasma_015 | ***Gemella haemolysans***  ***Human herpesvirus 4***  ***Klebsiella pneumoniae***  ***Rothia dentocariosa***  ***Staphylococcus aureus***  ***Streptococcus oralis***  ***Streptococcus parasanguinis***  ***Streptococcus vestibularis*** | **1257**  **300**  **4313**  **11179**  **20307**  **4130**  **9521**  **7008** | *Actinomyces odontolyticus*  ***Gemella haemolysans***  ***Human herpesvirus 4***  ***Klebsiella pneumoniae***  ***Rothia dentocariosa***  ***Staphylococcus aureus***  ***Streptococcus oralis***  ***Streptococcus parasanguinis***  ***Streptococcus vestibularis*** | No calls | No calls |
| plasma_016 | ***Human herpesvirus 1*** | **5513** | ***Human herpesvirus 1*** | ***Human herpesvirus 1*** | ***Human herpesvirus 1*** |
| plasma_017 | *Aggregatibacter segnis*  *Bacteroides vulgatus*  *Neisseria elongata*  ***Neisseria sicca***  *Prevotella melaninogenica*  *Staphylococcus haemolyticus* | 488  635  1569  **4454**  611  1034 | ***Neisseria sicca*** | No calls | No calls |
| plasma_018 | ***Anaerococcus tetradius***  ***Haemophilus parainfluenzae***  ***Neisseria cinerea***  *Neisseria elongata*  ***Neisseria flavescens***  *Neisseria sicca*  ***Rothia mucilaginosa*** | **762**  **771**  **901**  190  **293**  215  **131** | ***Anaerococcus tetradius***  *Fusobacterium periodonticum*  ***Haemophilus parainfluenzae***  ***Neisseria cinerea***  ***Neisseria flavescens***  ***Rothia mucilaginosa*** | No calls | No calls |
| plasma_019 | ***Bacteroides vulgatus***  ***Candida albicans***  *Enterococcus faecalis*  ***Klebsiella pneumoniae***  ***Prevotella denticola***  ***Prevotella oris***  ***Pseudomonas aeruginosa*** | **134**  **77**  430  **2667**  **455**  **1122**  **378** | ***Bacteroides vulgatus***  ***Candida albicans***  *Human herpesvirus 4*  ***Klebsiella pneumoniae***  ***Prevotella denticola***  ***Prevotella oris***  ***Pseudomonas aeruginosa*** | No calls | No calls |
| plasma_020 | ***Human herpesvirus 5***  ***Human herpesvirus 6B***  ***Rothia dentocariosa***  ***Rothia mucilaginosa***  ***Streptococcus parasanguinis*** | **428**  **1135**  **408**  **492**  **295** | *Actinomyces oris*  ***Human herpesvirus 5***  ***Human herpesvirus 6B***  ***Rothia dentocariosa***  ***Rothia mucilaginosa***  ***Streptococcus parasanguinis*** | ***Human herpesvirus 6B*** | ***Human herpesvirus 6B*** |
| plasma_021 | No calls |  | No calls | No calls | No calls |
| plasma_022 | ***Actinomyces graevenitzii***  ***Prevotella melaninogenica***  ***Rothia dentocariosa*** | **394**  **336**  **281** | ***Actinomyces graevenitzii***  *Actinomyces oris*  *Corynebacterium matruchotii*  *Kytococcus sedentarius*  *Megasphaera micronuciformis*  ***Prevotella melaninogenica***  *Propionibacterium propionicum*  ***Rothia dentocariosa***  *Rothia mucilaginosa*  *Veillonella dispar*  *Veillonella parvula* | No calls | No calls |
| plasma_023 | ***Bacteroides fragilis***  *Escherichia coli*  ***Human herpesvirus 7***  ***Pseudomonas aeruginosa*** | **499**  640  **13**  **64** | ***Bacteroides fragilis***  ***Human herpesvirus 7***  ***Pseudomonas aeruginosa*** | No calls | No calls |
| plasma_024 | ***Fusobacterium periodonticum***  ***Haemophilus parainfluenzae***  ***Neisseria mucosa***  ***Prevotella melaninogenica***  ***Rothia mucilaginosa***  ***Streptococcus mitis***  ***Veillonella dispar*** | **259**  **774**  **355**  **1890**  **761**  **171**  **342** | *Actinomyces odontolyticus*  *Capnocytophaga gingivalis*  ***Fusobacterium periodonticum***  *Gemella sanguinis*  ***Haemophilus parainfluenzae***  *Neisseria flavescens*  ***Neisseria mucosa***  ***Prevotella melaninogenica***  ***Rothia mucilaginosa***  ***Streptococcus mitis***  *Streptococcus oralis*  *Streptococcus parasanguinis*  ***Veillonella dispar*** | No calls | No calls |
| plasma_025 | ***Peptoniphilus harei***  ***Peptoniphilus lacrimalis***  ***Peptostreptococcus anaerobius*** | **501**  **805**  **958** | *Escherichia coli*  ***Peptoniphilus harei***  ***Peptoniphilus lacrimalis***  ***Peptostreptococcus anaerobius***  *Propionimicrobium lymphophilum* | No calls | No calls |
| plasma_026 | *Helicobacter pylori* | 260 | No calls** | No calls | No calls* |
| plasma_027 | *Actinomyces turicensis*  ***Candida tropicalis***  ***Clostridium perfringens***  ***Fusobacterium nucleatum***  ***Klebsiella variicola***  ***Parvimonas micra***  *Prevotella buccae*  ***Proteus vulgaris***  *Solobacterium moorei*  *Veillonella parvula* | 105  **444**  **739**  **1750**  **1892**  **359**  375  **492**  174  105 | ***Candida tropicalis***  ***Clostridium perfringens***  ***Fusobacterium nucleatum***  ***Klebsiella variicola***  ***Parvimonas micra***  ***Proteus vulgaris*** | No calls | No calls |
| plasma_028 | No calls |  | No calls | No calls | No calls |
| plasma_029 | No calls |  | No calls | No calls | No calls |
| plasma_030 | *Prevotella melaninogenica* | 113 | No calls | No calls | No calls |
| plasma_031 | *Streptococcus infantarius* | 663 | No calls | No calls | No calls |
| plasma_032 | No calls |  | No calls | No calls | No calls |
| plasma_033 | No calls |  | No calls | No calls | No calls |
| plasma_034 | *Candida albicans*  ***Prevotella melaninogenica***  ***Pseudomonas aeruginosa***  *Staphylococcus epidermidis*  ***Streptococcus mitis***  ***Veillonella dispar*** | 37  **1107**  **13985**  1047  **453**  **357** | *Actinomyces odontolyticus*  *Haemophilus parainfluenza*  ***Prevotella melaninogenica***  ***Pseudomonas aeruginosa***  *Rothia mucilaginosa*  ***Streptococcus mitis***  ***Veillonella dispar*** | No calls | ***Pseudomonas aeruginosa*** |
| plasma_035 | ***Candida albicans***  ***Human herpesvirus 1***  ***Human herpesvirus 4***  ***Human herpesvirus 5***  ***Staphylococcus epidermidis***  *Toxoplasma gondii*  ***Veillonella parvula*** | **3300**  **1523**  **9773**  **4061**  **53179**  63  **5049** | ***Candida albicans***  *Capnocytophaga gingivalis*  ***Human herpesvirus 1***  ***Human herpesvirus 4***  ***Human herpesvirus 5***  ***Staphylococcus epidermidis***  ***Veillonella parvula*** | No calls | ***Human herpesvirus 5*** |
| plasma_036 | ***Candida dubliniensis***  ***Enterococcus avium***  ***Enterococcus faecalis***  ***Prevotella bivia***  ***Prevotella buccae***  ***Prevotella denticola***  ***Prevotella loescheii***  ***Staphylococcus aureus***  ***Veillonella parvula*** | **208**  **346**  **1272**  **738**  **502**  **364**  **885**  **622**  **1188** | ***Candida dubliniensis***  ***Enterococcus avium***  ***Enterococcus faecalis***  ***Prevotella bivia***  ***Prevotella buccae***  ***Prevotella denticola***  ***Prevotella loescheii***  ***Staphylococcus aureus***  ***Veillonella parvula*** | No calls | No calls |
|  |  |  |  |  |  |
| **Samples with called microbes:** | **28** |  | **25** | **4** | **8** |
| **Samples with no calls:** | **8** |  | **11** | **32** | **28** |
| **Total called microbes:** | **165** |  | **155** | **5** | **11** |
| **Total unique taxa:** | **85** |  | **80** | **5** | **11** |

* *Bacillus pumilus* was preliminarily detected in plasma_026 using the Ext+ssDNA approach, but follow-up dilution series testing revealed it to be sporadic environmental and/or reagent contamination (see **Supplementary Text A**).

** *Kytococcus sedendatrius* and *Micrococcus lylae* were originally detected in plasma_026 using the DC3 platform, but comparison to the original DC3 commercial sample revealed it to be a sporadic contamination (see **Supplementary Text A**).

**Supplementary Table 4.**

Panel of 13 representative microbes (“P13 mix”) used for contrived specimens.

| **Representative Microbial DNA** | **Pathogen Class** | **%-GC** | **Genome Size** | **Common Commensal** | **Common EC** | **Source** |
| --- | --- | --- | --- | --- | --- | --- |
| *Aspergillus fumigatus* | Eukaryote Mold | 50 | 29 Mb | - | - | ATCC |
| *Bordetella pertussis* | Gram Neg Bacteria | 68 | 4.1 Mb | - | - | ATCC |
| *Cryptosporidium parvum* | Eukaryotic Parasite | 30 | 9.1 Mb | - | - | ATCC |
| *Escherichia coli* | Gram Neg Bacteria | 51 | 5.62 Mb | Yes, gut | Yes | ATCC |
| *Human adenovirus* | dsDNA Virus | 51 | 35 kb | - | - | IDT |
| *Leishmania major* | Eukaryotic Parasite | 63 | 33 Mb | - | - | ATCC |
| *Mycobacterium tuberculosis* | Acid Fast Bacteria | 66 | 4.41 Mb | - | - | ATCC |
| *Plasmodium falciparum* | Eukaryotic Parasite | 19 | 22.9 Mb | - | - | ATCC |
| *Pseudomonas aeruginosa* | Gram Neg Bacteria | 66 | 6.3 Mb | Yes, skin | Yes | NIST |
| *Salmonella enterica* | Gram Neg Bacteria | 52 | 4.77 Mb | - | - | NIST |
| *Shigella flexneri* | Gram Neg Bacteria | 51 | 4.60 Mb | - | - | ATCC |
| *Staphylococcus aureus* | Gram Pos Bacteria | 32 | 2.8 Mb | Yes, nose | - | NIST |
| *Staphylococcus epidermidis* | Gram Pos Bacteria | 32 | 2.56 Mb | Yes, skin | Yes | ATCC |

**Supplementary Table 5.**

Endogenous interfering substances tested for the devices with FDA 510(k) clearance that utilize plasma as the input sample type and PCR/sequencing-based DNA detection in cancer and infectious disease diagnostics.

| **Premarket Approval application (PMA) number** | **P210040** | **P200010** | **P190032** | **P190004** |
| --- | --- | --- | --- | --- |
| **Year** | 2022 | 2020 | 2020 | 2019 |
| **Data resource** | SSED | SSED | SSED | SSED |
| **Appliant** | Resolution Bioscience | Guardant Health | FoundationOne® Liquid CDx | Qiagen GmbH |
| **Device trade name** | Agilent Resolution ctDx FIRST | Guardant360® CDx | FoundationOne® Liquid CDx | *therascreen* PIK3CA RGQ PCR Kit |
| **Sample type** | Plasma | Plasma | Plasma | Plasma |
| **DNA** | cfDNA | cfDNA | cfDNA | ctDNA |
| **detection method** | Sequencing | Sequencing | Sequencing | Real time PCR |
| **DNA extraction** | Y | Y | Y | Y |
| **Library** | Library and hybridization | Library and hybridization | Library and hybridization | No |
| **Purpose** | Cancer | Cancer | Cancer | Cancer |
| **Type** | Non-small cell lung cancer (NSCLC) | Non-small cell lung cancer (NSCLC) | Non-small cell lung cancer (NSCLC); Prostate cancer | Breast cancer |
| **Endogenous interferents** | **6 targets in total:**  Hemoglobin  Albumin  Unconjugated bilirubin  Conjugated bilirubin  Glyceryl trioleate (Triglycerides)  High molecular weight gDNA | **6 targets in total**  Hemoglobin  Albumin  Unconjugated bilirubin  Conjugated bilirubin  Triglycerides  Staphylococcus epidermidis | **6 targets in total**  Hemoglobin  Albumin  Unconjugated bilirubin  Conjugated bilirubin  Glyceryl trioleate (Triglycerides)  Cholesterol | **6 targets in total**  Hemoglobin  Albumin  Unconjugated bilirubin  Conjugated bilirubin  EDTA  Caffeine |
|  |  |  |  |  |
|  |  |  |  |  |
|  |  |  |  |  |
|  |  |  |  |  |
|  |  |  |  |  |
|  |  |  |  |  |
|  |  |  |  |  |
|  |  |  |  |  |

**Supplementary Table 6.**

Results of interference testing for all endogenous and exogenous substances included in the study. The tested concentration levels were either *low* (3x LoD) or *high* (30x LoD); CI is defined as 95% confidence interval on the mean relative difference between the Control and Test samples, according to the CLSI guideline for paired-difference testing of potential interferents. The acceptance criterion for CI was set to be fully contained within the range (-63.3, 211).

| **Substance tested for interference** | **Microbial species** | **Concentration level (low/high)** | **CI (% relative difference in mcfDNA concentration)** | **Acceptance criteria**  **(pass/fail)** |
| --- | --- | --- | --- | --- |
| **Plasma proteins** | Pseudomonas aeruginosa | low | ( 11.3838 , 75.849 ) | pass |
|  | Bordetella pertussis | low | ( 12.5085 , 69.499 ) | pass |
|  | Escherichia coli | low | ( -4.0412 , 51.839 ) | pass |
|  | Shigella flexneri | low | ( -4.5206 , 59.741 ) | pass |
|  | Staphylococcus aureus | low | ( -16.7344 , 21.417 ) | pass |
|  | Staphylococcus epidermidis | low | ( -11.6939 , 34.290 ) | pass |
|  | Mycobacterium tuberculosis | low | ( -0.0499 , 66.179 ) | pass |
|  | Leishmania major | low | ( 0.8375 , 65.838 ) | pass |
|  | Cryptosporidium parvum | low | ( -18.2605 , 19.953 ) | pass |
|  | Plasmodium falciparum | low | ( -25.5901 , 10.464 ) | pass |
|  | Salmonella enterica | low | ( -3.8916 , 47.951 ) | pass |
|  | Human mastadenovirus B | low | ( 10.9359 , 64.523 ) | pass |
|  | Aspergillus fumigatus | low | ( 4.3236 , 57.054 ) | pass |
|  | Pseudomonas aeruginosa | high | ( 2.2411 , 33.359 ) | pass |
|  | Bordetella pertussis | high | ( 4.5812 , 34.560 ) | pass |
|  | Escherichia coli | high | ( -2.4464 , 25.775 ) | pass |
|  | Shigella flexneri | high | ( -3.9476 , 26.671 ) | pass |
|  | Staphylococcus aureus | high | ( -18.4853 , 8.411 ) | pass |
|  | Staphylococcus epidermidis | high | ( -18.6761 , 8.191 ) | pass |
|  | Mycobacterium tuberculosis | high | ( 3.4492 , 34.486 ) | pass |
|  | Leishmania major | high | ( -3.8724 , 27.176 ) | pass |
|  | Cryptosporidium parvum | high | ( -21.2488 , 5.086 ) | pass |
|  | Plasmodium falciparum | high | ( -27.1920 , -0.327 ) | pass |
|  | Salmonella enterica | high | ( -5.1473 , 24.460 ) | pass |
|  | Human mastadenovirus B | high | ( 2.0282 , 30.934 ) | pass |
|  | Aspergillus fumigatus | high | ( -3.6643 , 26.027 ) | pass |
| **Hemolysate** | Pseudomonas aeruginosa | low | ( -1.07 , 58.0 ) | pass |
|  | Bordetella pertussis | low | ( -4.75 , 44.8 ) | pass |
|  | Escherichia coli | low | ( 14.10 , 62.0 ) | pass |
|  | Shigella flexneri | low | ( 11.13 , 61.1 ) | pass |
|  | Staphylococcus aureus | low | ( 11.79 , 62.0 ) | pass |
|  | Staphylococcus epidermidis | low | ( 0.15 , 50.1 ) | pass |
|  | Mycobacterium tuberculosis | low | ( -14.66 , 25.0 ) | pass |
|  | Leishmania major | low | ( 13.67 , 61.3 ) | pass |
|  | Cryptosporidium parvum | low | ( 11.61 , 56.9 ) | pass |
|  | Plasmodium falciparum | low | ( 4.42 , 59.1 ) | pass |
|  | Salmonella enterica | low | ( 13.65 , 59.6 ) | pass |
|  | Human mastadenovirus B | low | ( 11.52 , 58.3 ) | pass |
|  | Aspergillus fumigatus | low | ( 12.80 , 71.8 ) | pass |
|  | Pseudomonas aeruginosa | high | ( 15.26 , 41.7 ) | pass |
|  | Bordetella pertussis | high | ( 28.82 , 53.4 ) | pass |
|  | Escherichia coli | high | ( 32.98 , 58.2 ) | pass |
|  | Shigella flexneri | high | ( 37.94 , 64.7 ) | pass |
|  | Staphylococcus aureus | high | ( 34.43 , 60.6 ) | pass |
|  | Staphylococcus epidermidis | high | ( 32.01 , 56.7 ) | pass |
|  | Mycobacterium tuberculosis | high | ( 24.57 , 46.6 ) | pass |
|  | Leishmania major | high | ( 37.31 , 61.3 ) | pass |
|  | Cryptosporidium parvum | high | ( 29.68 , 58.1 ) | pass |
|  | Plasmodium falciparum | high | ( 35.66 , 58.3 ) | pass |
|  | Salmonella enterica | high | ( 35.52 , 61.1 ) | pass |
|  | Human mastadenovirus B | high | ( 37.51 , 63.0 ) | pass |
|  | Aspergillus fumigatus | high | ( 34.60 , 59.8 ) | pass |
| **Lipids** | Bordetella pertussis | low | ( -11.51 , 2.682 ) | pass |
|  | Shigella flexneri | low | ( -60.08 , -47.519 ) | pass |
|  | Staphylococcus aureus | low | ( -10.03 , 3.925 ) | pass |
|  | Staphylococcus epidermidis | low | ( -6.89 , 5.192 ) | pass |
|  | Mycobacterium tuberculosis | low | ( -7.48 , 8.798 ) | pass |
|  | Leishmania major | low | ( -10.26 , 0.801 ) | pass |
|  | Cryptosporidium parvum | low | ( -11.25 , 0.561 ) | pass |
|  | Plasmodium falciparum | low | ( -5.15 , 4.299 ) | pass |
|  | Salmonella enterica | low | ( -14.93 , -0.975 ) | pass |
|  | Human mastadenovirus B | low | ( -19.40 , -1.419 ) | pass |
|  | Aspergillus fumigatus | low | ( -12.56 , 7.752 ) | pass |
|  | Bordetella pertussis | high | ( -16.26 , 14.353 ) | pass |
|  | Shigella flexneri | high | ( -27.60 , 1.707 ) | pass |
|  | Staphylococcus aureus | high | ( -12.30 , 17.940 ) | pass |
|  | Staphylococcus epidermidis | high | ( -12.84 , 17.579 ) | pass |
|  | Mycobacterium tuberculosis | high | ( -15.60 , 15.197 ) | pass |
|  | Leishmania major | high | ( -14.33 , 15.316 ) | pass |
|  | Cryptosporidium parvum | high | ( -14.18 , 16.706 ) | pass |
|  | Plasmodium falciparum | high | ( -12.81 , 17.253 ) | pass |
|  | Salmonella enterica | high | ( -17.68 , 12.552 ) | pass |
|  | Human mastadenovirus B | high | ( -15.30 , 13.944 ) | pass |
|  | Aspergillus fumigatus | high | ( -14.64 , 15.892 ) | pass |
| **Conjugated bilirubin** | Pseudomonas aeruginosa | low | ( -21.95 , -4.381 ) | pass |
|  | Bordetella pertussis | low | ( -18.68 , 0.283 ) | pass |
|  | Escherichia coli | low | ( -18.08 , 1.420 ) | pass |
|  | Shigella flexneri | low | ( -20.28 , 0.924 ) | pass |
|  | Staphylococcus aureus | low | ( -13.96 , 1.409 ) | pass |
|  | Staphylococcus epidermidis | low | ( -14.84 , -1.170 ) | pass |
|  | Mycobacterium tuberculosis | low | ( -22.33 , -3.472 ) | pass |
|  | Leishmania major | low | ( -23.01 , 0.342 ) | pass |
|  | Cryptosporidium parvum | low | ( -11.25 , 2.686 ) | pass |
|  | Plasmodium falciparum | low | ( -8.84 , 7.769 ) | pass |
|  | Salmonella enterica | low | ( -20.88 , -2.407 ) | pass |
|  | Human mastadenovirus B | low | ( -23.39 , -4.628 ) | pass |
|  | Aspergillus fumigatus | low | ( -23.39 , -4.000 ) | pass |
|  | Pseudomonas aeruginosa | high | ( -25.01 , -2.482 ) | pass |
|  | Bordetella pertussis | high | ( -22.87 , -1.778 ) | pass |
|  | Escherichia coli | high | ( -16.12 , -0.918 ) | pass |
|  | Shigella flexneri | high | ( -18.35 , 0.660 ) | pass |
|  | Staphylococcus aureus | high | ( -9.71 , 3.859 ) | pass |
|  | Staphylococcus epidermidis | high | ( -10.51 , 2.998 ) | pass |
|  | Mycobacterium tuberculosis | high | ( -23.77 , -1.337 ) | pass |
|  | Leishmania major | high | ( -22.11 , -0.579 ) | pass |
|  | Cryptosporidium parvum | high | ( -9.59 , 4.266 ) | pass |
|  | Plasmodium falciparum | high | ( -7.11 , 8.514 ) | pass |
|  | Salmonella enterica | high | ( -19.24 , -0.745 ) | pass |
|  | Human mastadenovirus B | high | ( -16.89 , -3.902 ) | pass |
|  | Aspergillus fumigatus | high | ( -17.84 , -2.624 ) | pass |
| **Unconjugated bilirubin** | Pseudomonas aeruginosa | low | ( -14.91 , 6.436 ) | pass |
|  | Bordetella pertussis | low | ( -12.39 , 0.173 ) | pass |
|  | Staphylococcus aureus | low | ( -11.28 , 8.671 ) | pass |
|  | Staphylococcus epidermidis | low | ( -3.22 , 14.270 ) | pass |
|  | Mycobacterium tuberculosis | low | ( -15.45 , 17.487 ) | pass |
|  | Leishmania major | low | ( -14.62 , 6.601 ) | pass |
|  | Cryptosporidium parvum | low | ( -5.40 , 12.040 ) | pass |
|  | Plasmodium falciparum | low | ( -4.80 , 10.840 ) | pass |
|  | Human mastadenovirus B | low | ( -19.37 , 0.390 ) | pass |
|  | Pseudomonas aeruginosa | high | ( -15.88 , -1.059 ) | pass |
|  | Bordetella pertussis | high | ( -15.18 , -1.634 ) | pass |
|  | Staphylococcus aureus | high | ( -10.04 , 0.883 ) | pass |
|  | Staphylococcus epidermidis | high | ( -8.02 , 3.274 ) | pass |
|  | Mycobacterium tuberculosis | high | ( -11.58 , 1.885 ) | pass |
|  | Leishmania major | high | ( -10.39 , 2.910 ) | pass |
|  | Cryptosporidium parvum | high | ( -9.44 , 3.516 ) | pass |
|  | Plasmodium falciparum | high | ( -9.44 , 5.249 ) | pass |
|  | Human mastadenovirus B | high | ( -17.70 , -5.322 ) | pass |
| **K_2_EDTA** | Pseudomonas aeruginosa | low | ( -32.8 , -3.71 ) | pass |
|  | Bordetella pertussis | low | ( -33.4 , 1.43 ) | pass |
|  | Shigella flexneri | low | ( -35.8 , -2.60 ) | pass |
|  | Staphylococcus aureus | low | ( -36.2 , -7.70 ) | pass |
|  | Staphylococcus epidermidis | low | ( -35.0 , -8.07 ) | pass |
|  | Mycobacterium tuberculosis | low | ( -33.2 , -2.07 ) | pass |
|  | Leishmania major | low | ( -37.3 , -11.17 ) | pass |
|  | Cryptosporidium parvum | low | ( -35.8 , -7.83 ) | pass |
|  | Plasmodium falciparum | low | ( -38.1 , -6.68 ) | pass |
|  | Salmonella enterica | low | ( -36.6 , -3.79 ) | pass |
|  | Human mastadenovirus B | low | ( -35.3 , -11.90 ) | pass |
|  | Aspergillus fumigatus | low | ( -38.5 , -10.05 ) | pass |
|  | Pseudomonas aeruginosa | high | ( -37.8 , 12.97 ) | pass |
|  | Bordetella pertussis | high | ( -37.3 , 11.17 ) | pass |
|  | Shigella flexneri | high | ( -38.5 , 8.68 ) | pass |
|  | Staphylococcus aureus | high | ( -35.9 , 6.33 ) | pass |
|  | Staphylococcus epidermidis | high | ( -37.0 , 6.37 ) | pass |
|  | Mycobacterium tuberculosis | high | ( -39.3 , 13.57 ) | pass |
|  | Leishmania major | high | ( -39.6 , 7.89 ) | pass |
|  | Cryptosporidium parvum | high | ( -36.4 , 6.00 ) | pass |
|  | Plasmodium falciparum | high | ( -35.0 , 7.36 ) | pass |
|  | Salmonella enterica | high | ( -38.0 , 8.85 ) | pass |
|  | Human mastadenovirus B | high | ( -36.7 , 10.22 ) | pass |
|  | Aspergillus fumigatus | high | ( -36.6 , 8.48 ) | pass |

**Supplementary Table 7.**

Microbial taxa detected in the interfering substance standards and removed from the analysis.

| **Interfering Substance** | **Taxa removed** |
| --- | --- |
| Plasma proteins | None detected |
| Hemolysate | None detected |
| Lipids | *Escherichia coli, Pseudomonas aeruginosa* |
| Conjugated bilirubin | None detected |
| Unconjugated bilirubin | *Aspergillus fumigatus, Escherichia coli, Salmonella enterica, Staphylococcus aureus* |
| K_2_EDTA | *Escherichia coli* |

#

# Supplementary Figures


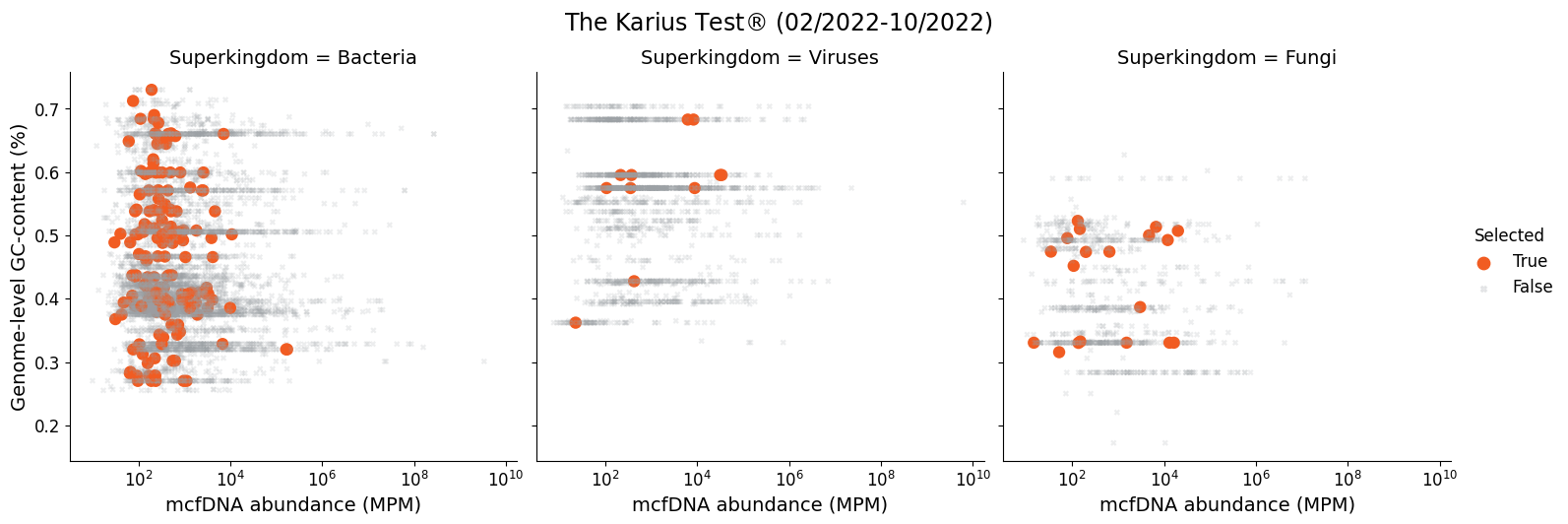


**Supplementary Figure 1. Samples selected for study reflect diversity of mcfDNA species in human plasma.** A subset of these samples (28) featured called microbes (shown in orange) with a wide range of phylogenetic diversity, GC-content, and mcfDNA concentrations in plasma as compared to the background of production samples previously tested by The Karius Test^®^ from February to October of 2022 (shown in gray). In addition, 8 samples without previously called microbes were also included for processing in this study.

**A**


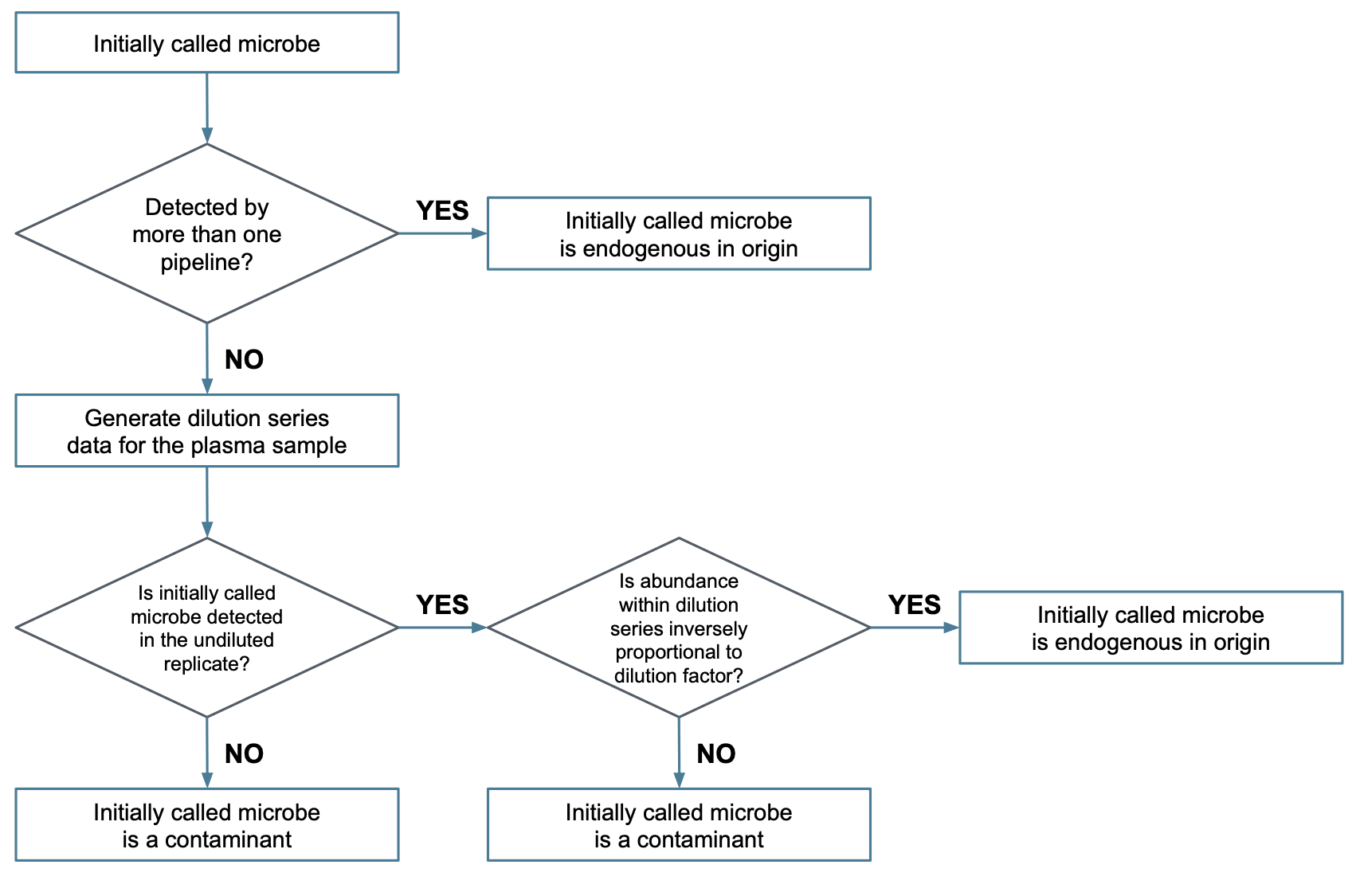


**B**
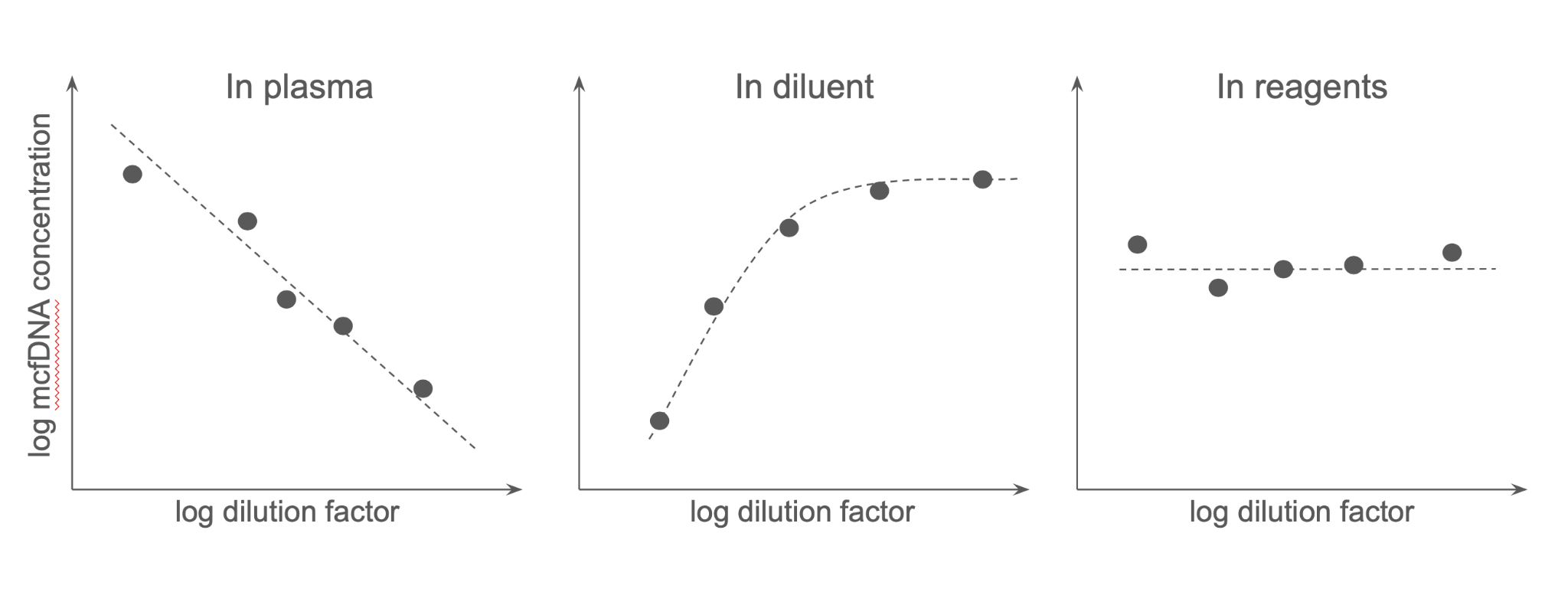


**Supplementary Figure 2.** (**A**) Schematic of the algorithm for determining the origin of the initially *called microbes*. (**B**) Schematic of expected relationship between the dilution factor and microbial concentration in the dilution series, illustrating three possible microbial origins (plasma, diluent, reagents) using a simulation of real life data for the points. If the microbe originates from the plasma, we expect a negative relationship between microbial concentration and the dilution factor. For microbes present in the diluent, we would observe an increase in microbial concentration as the dilution factor increases. For microbes originating from reagent background, we would expect a constant abundance across all dilution factors. The dilution factor is defined as (V_plasma_ + V_diluent_) / V_plasma_.

**A**
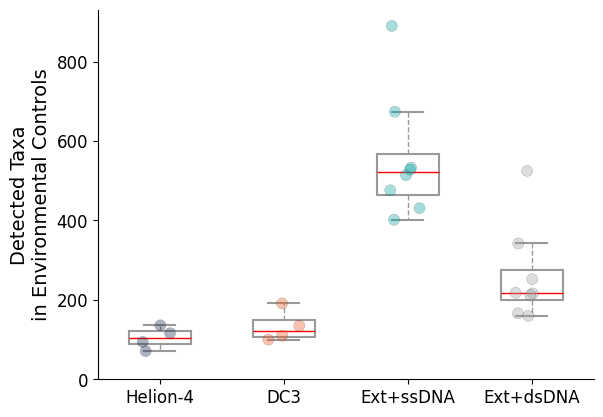


**B**
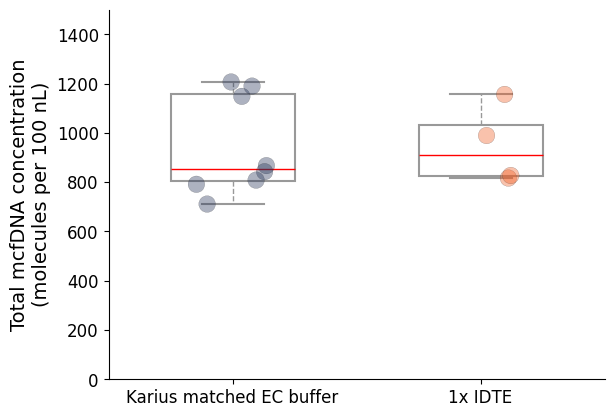


**Supplementary Figure 3. Comparison of number of detected taxa in the 1X IDTE buffer**. 1X IDTE buffer as an Environmental Control was tested in each of the four methods (N=4 for Karius Helion-4 and Karius DC3 and N=8 for Ext+dsDNA and Ext+ssDNA). (**A**) Comparison of taxa detected in each method with 1X IDTE buffer. Median taxa detected was 105 in Helion-4 (N=4), 122.5 in Karius DC3 (N=4, *p*=0.47), 217 in Ext+dsDNA (N=8, *p*=0.042), and 521 in Ext+ssDNA (N=8, *p*=0.042). (**B**) Comparison of total mcfDNA concentration detected in Helion-4 between the Karius matched EC buffer and 1X IDTE. Median mcfDNA concentration is 854 (N=8) and 908 (N=4, *p*=0.93) molecules per 100 nL, respectively.

**A**


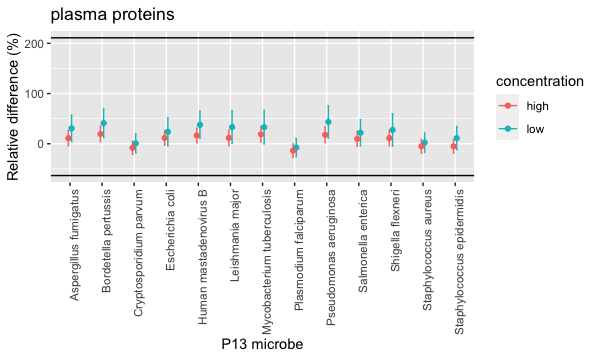


**B**


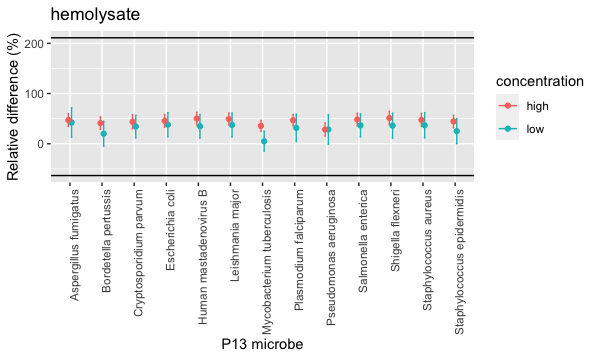


**C**


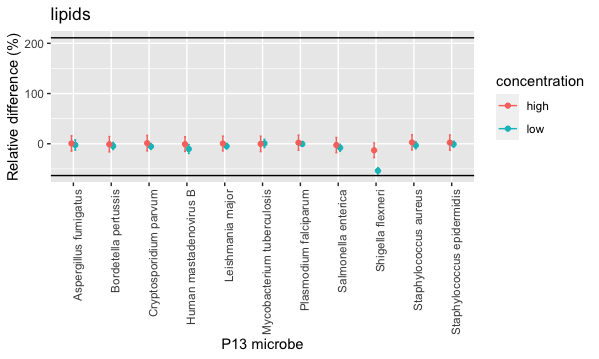


**D**


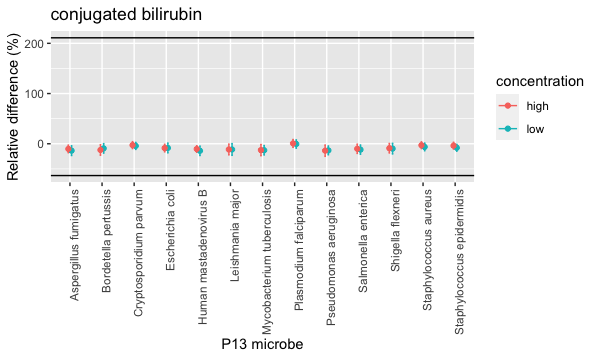


**E**


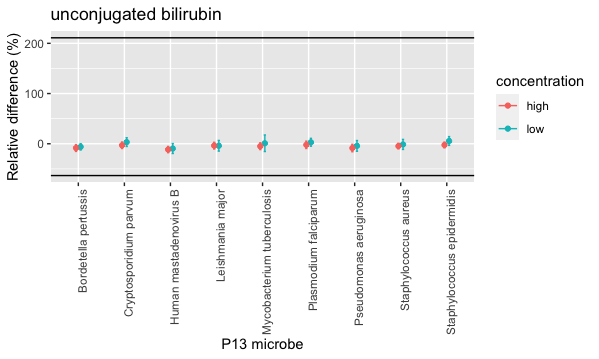


**F**


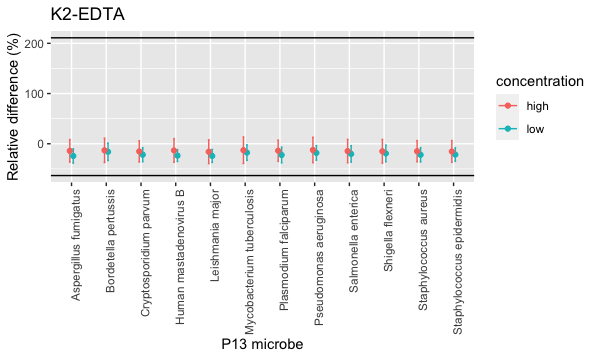


**Supplementary Figure 4. Concentration of mcfDNA standards in the presence of potential interfering substances.** The relative difference (in %) between mcfDNA concentrations for the microbial species included in mcfDNA standard at test concentration and background (control) concentration of (**A**) total plasma protein, (**B**) hemolysate, (**C**) lipids, (**D**) conjugated bilirubin, (**E**) unconjugated bilirubin, and (**F**) K_2_EDTA. The black lines in each plot indicate the acceptance criteria range of (-63.3, 211).

**A**


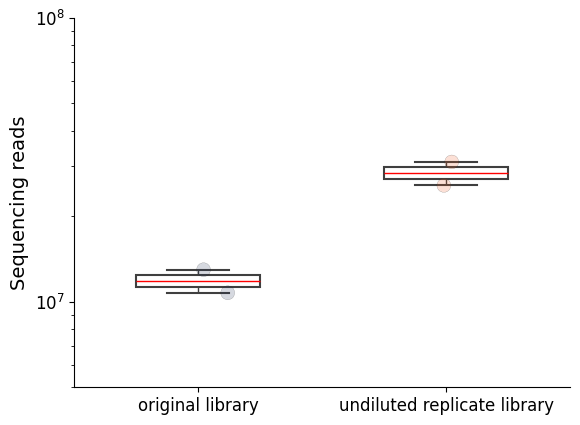


**B**


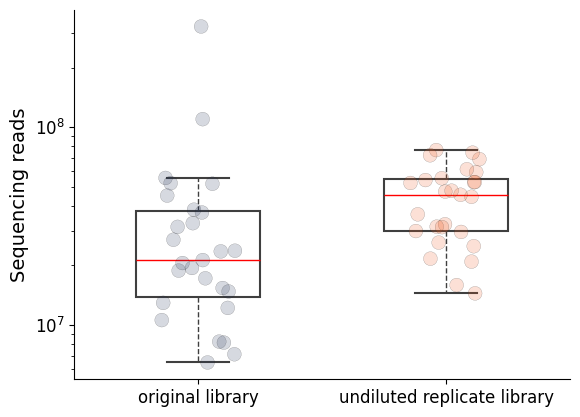


**C**


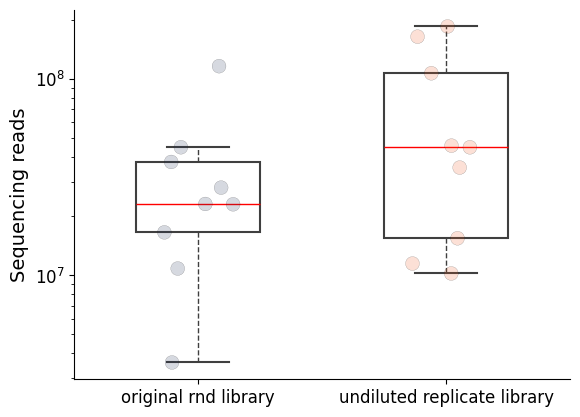


**Supplementary Figure 5. Sequencing depth of the libraries derived from the undiluted plasma as part of a dilution series compared to the original libraries.** (**A**) Ext+ssDNA dilution series, (**B**) Karius Helion-4 dilution series, and (**C**) Karius DC3 dilution series.

**A**


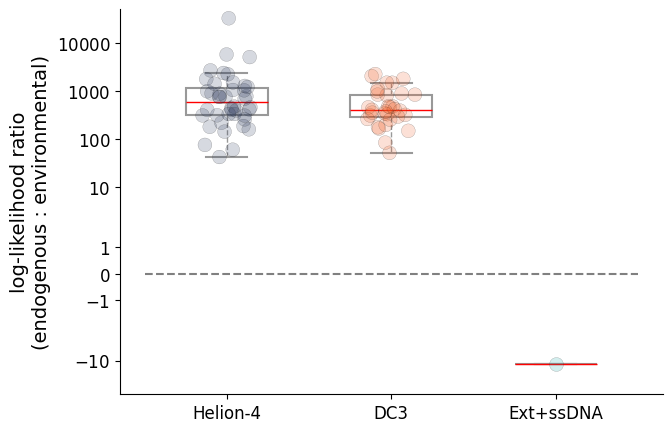


**B**


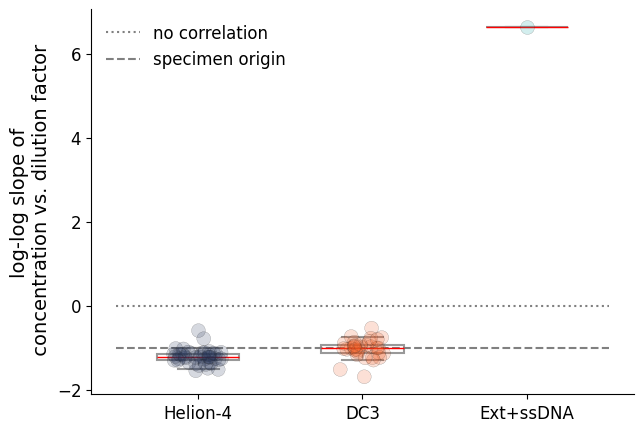


**C**


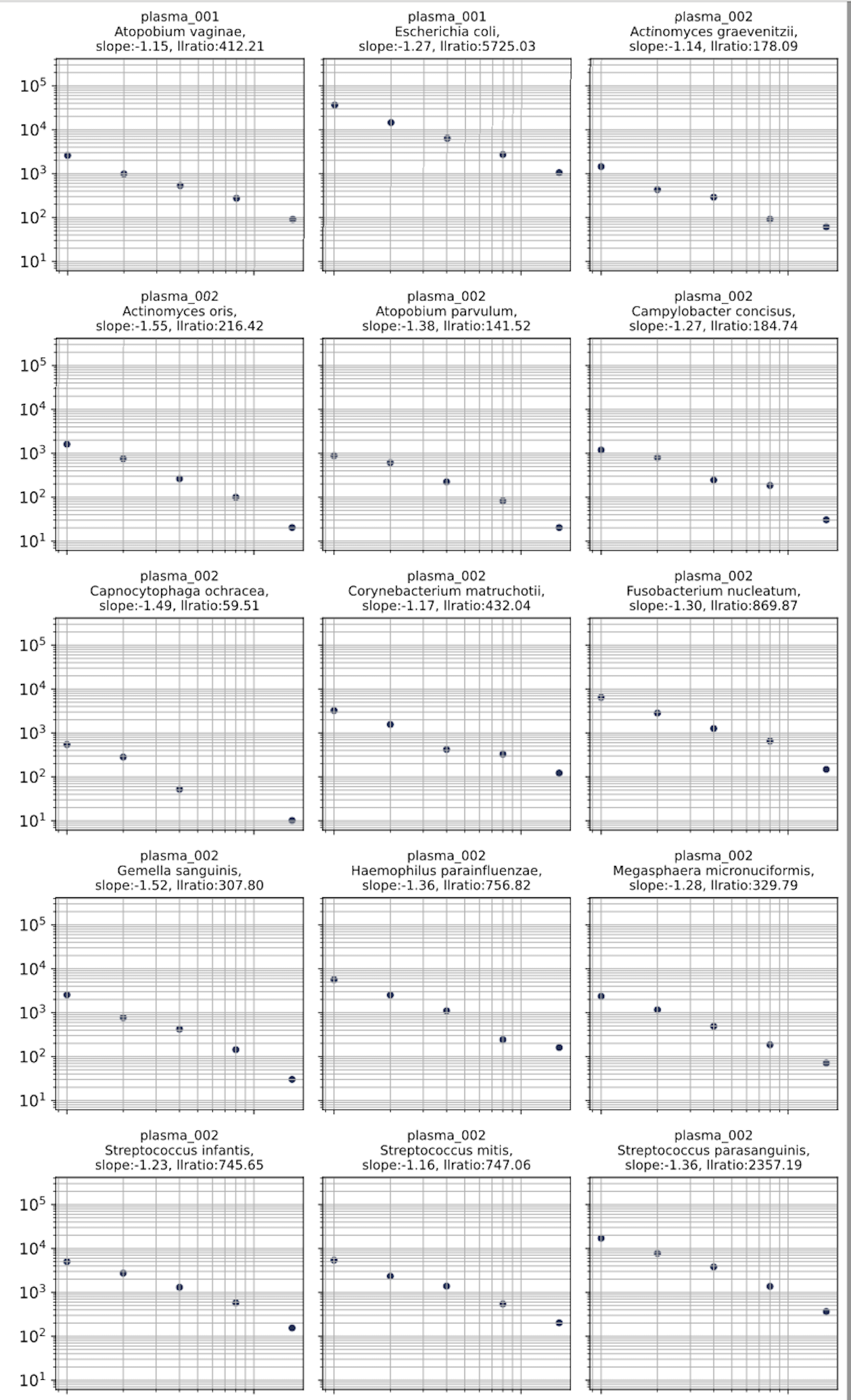


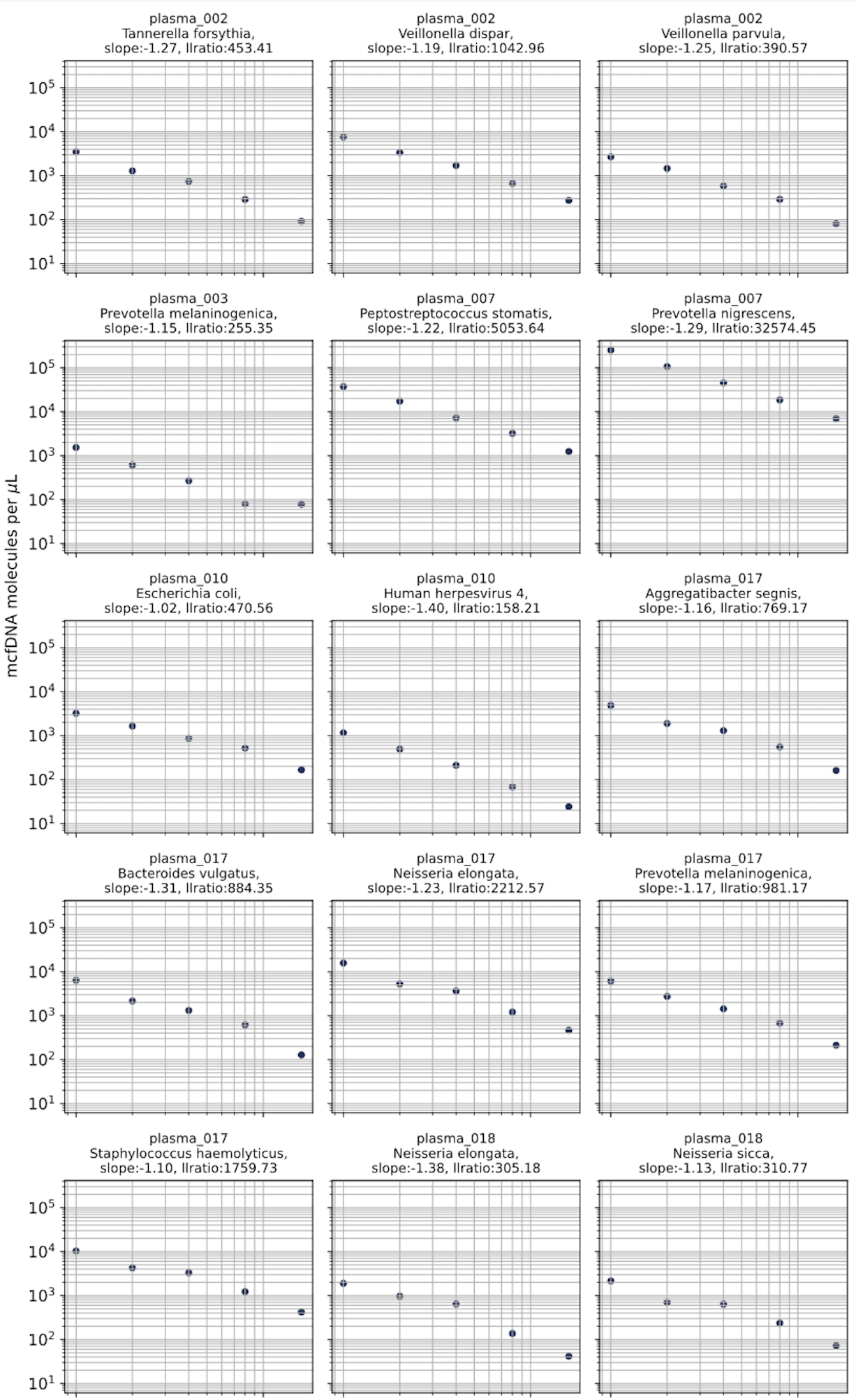


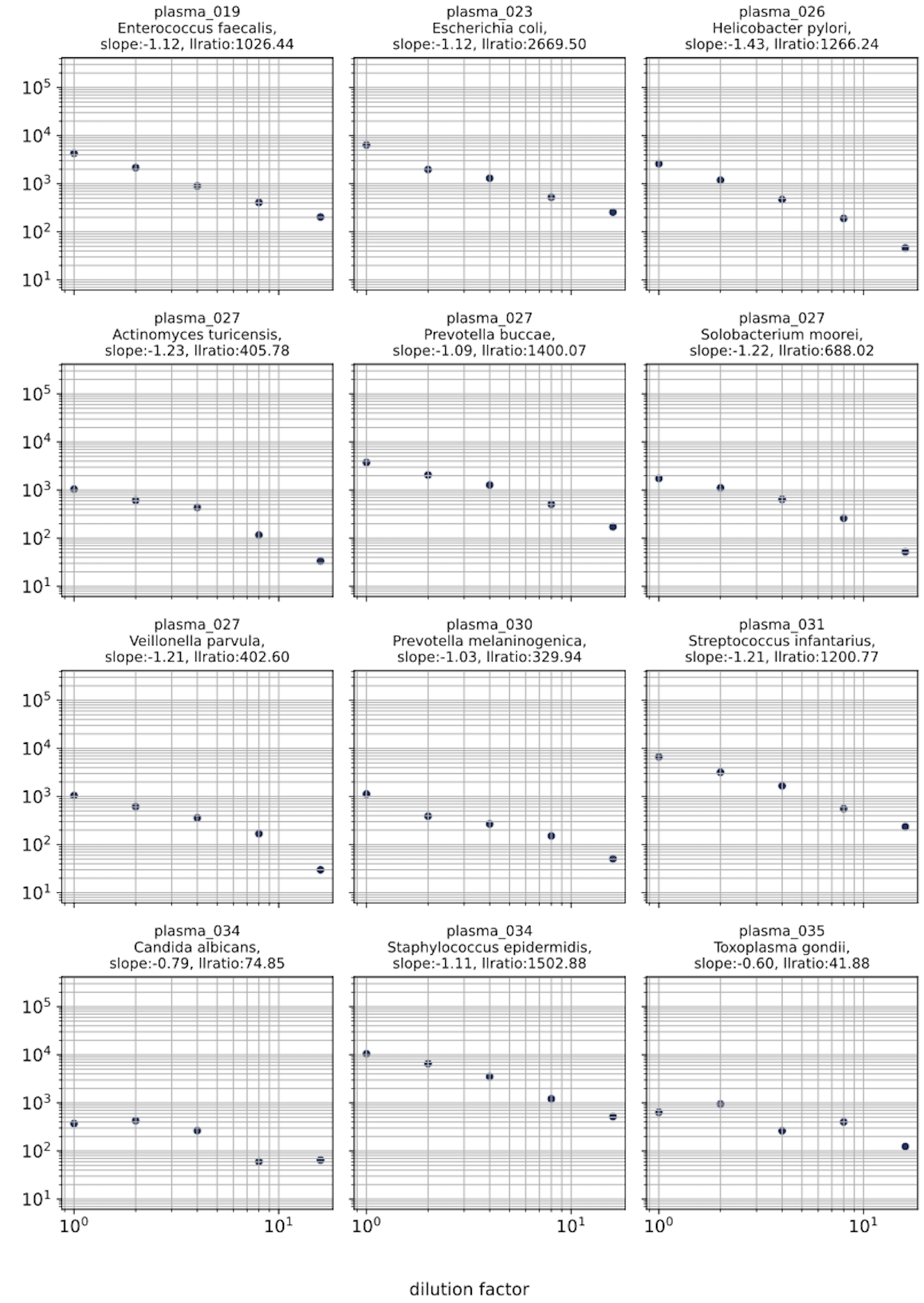


**D**


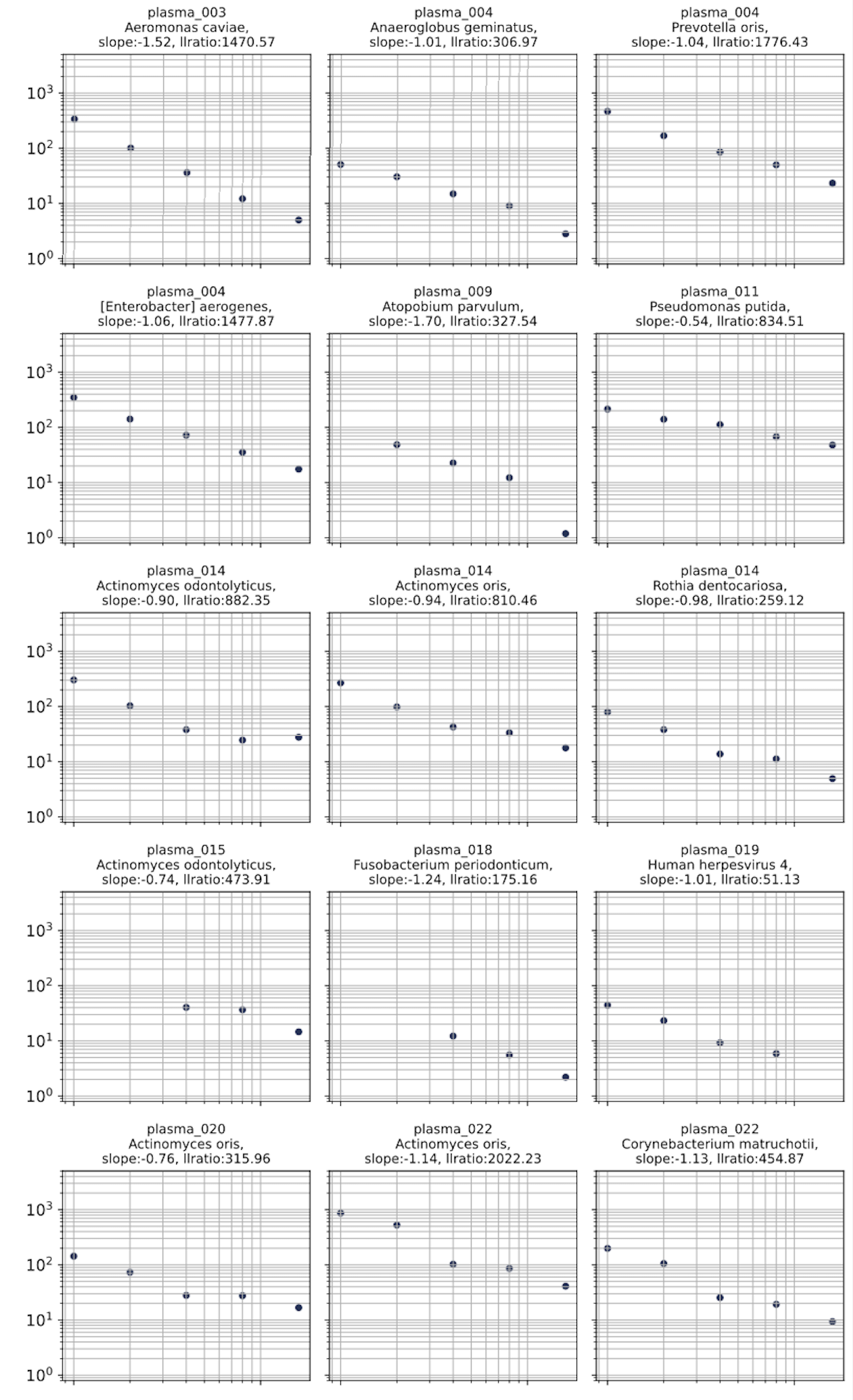


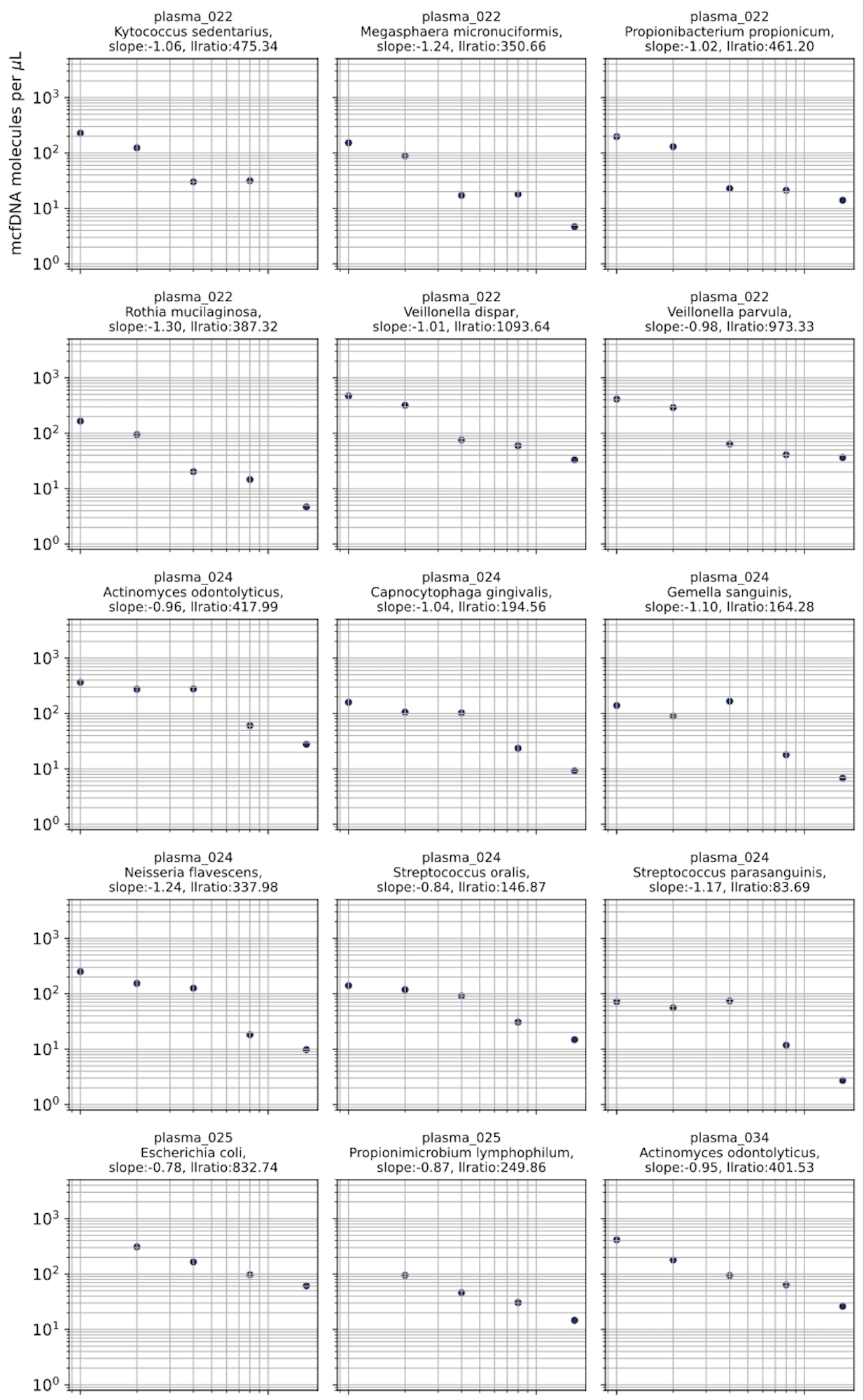


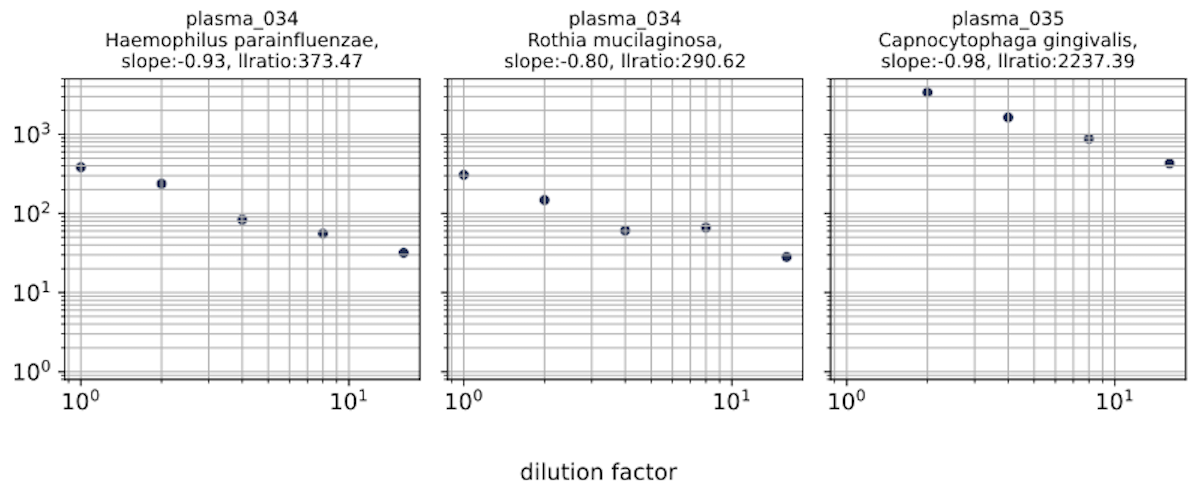


**E**


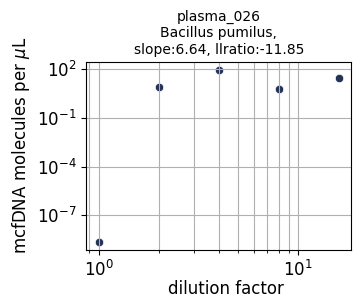


**Supplementary Figure 6. Examples of microbial signals in the context of diluted background samples.** Distribution of the (**A**) log-likelihood ratios and (**B**) log-log slopes of concentration vs. dilution factor for the microbes unique to each chemistry. The dashed line in **(A)** shows the log-likelihood ratio value at which there is equal likelihood microbe came from plasma vs. environment. The dashed and dotted lines in **(B)** shows the log-log slope expected if the microbe is endogenous to the plasma specimen and if there is no relationship between microbial abundance and dilution factor, respectively. Scatterplots showing the relationship between the microbial concentration and dilution factor for all of the unique microbes that underwent a dilution series for (**C**) Helion-4, (**D**) DC3, and (**E**) Ext+ssDNA. The plots are annotated with the log-log slope and the log-likelihood ratio, with positive log-likelihood ratio values indicating a higher likelihood of the microbe being endogenous to the plasma sample.
